# Supplementary material for: Establishment of centromere identity is dependent on nuclear spatial organization
Source: Curr Biol. 2022 Jul 25;32(14):3121–3136.e6. doi: 10.1016/j.cub.2022.06.048 (PMC9616734; doi:10.1016/j.cub.2022.06.048)
Supplement: Document S1. Figures S1–S7 and Tables S1 and S2 [file mmc1.pdf]

Current Biology, Volume *32*

Supplemental Information

Establishment of centromere identity

is dependent on nuclear spatial organization

Weifang Wu, Toni McHugh, David A. Kelly, Alison L. Pidoux, and Robin C. Allshire

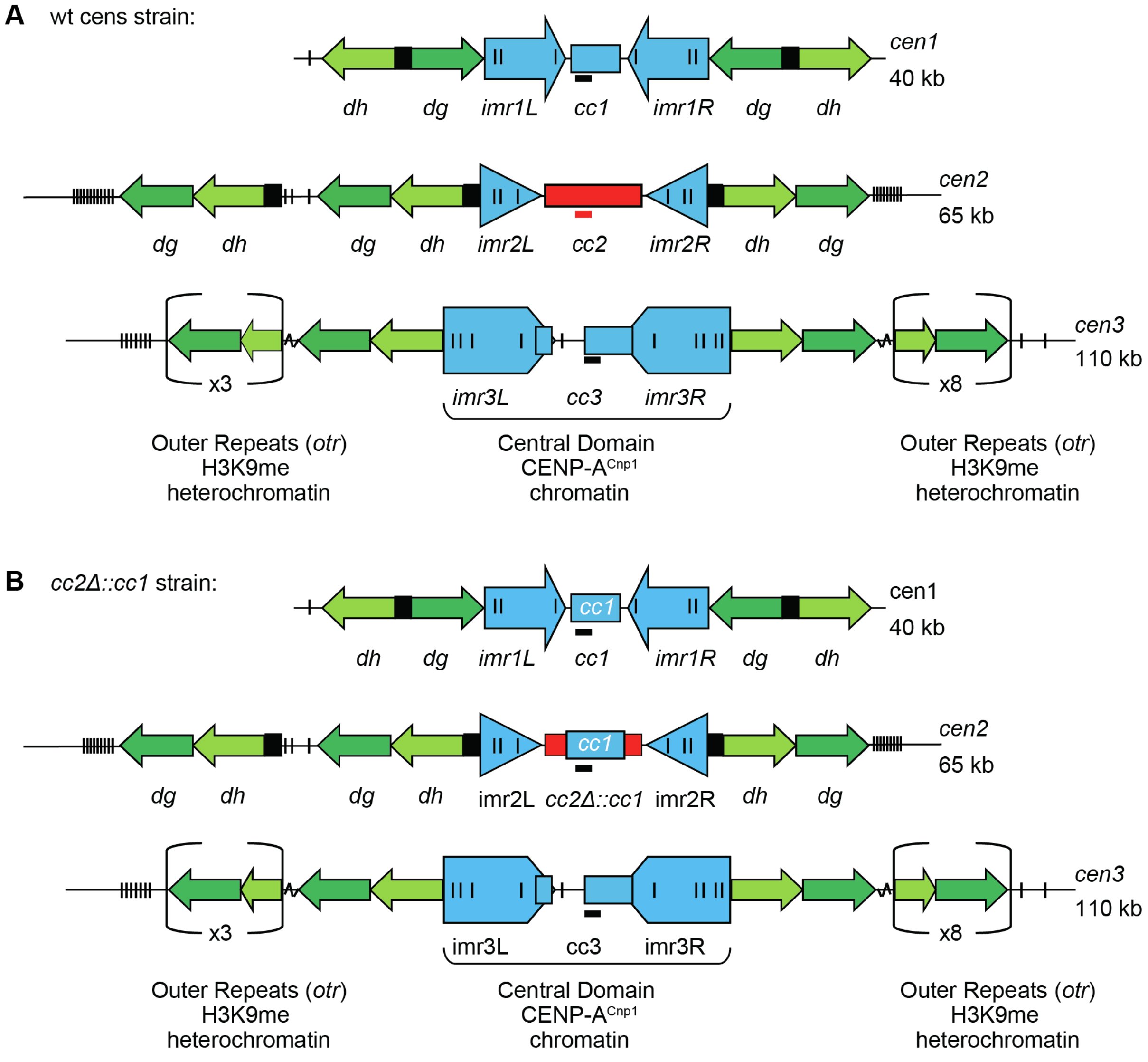

Figure S1. Domain organization of centromeres in fission yeast. Related to Figures 1-to-7.

(A, B) Schematic representation of the three endogenous wild-type centromeres - wt-cens (A) or *cc2Δ::cc1* strains (B). Each centromere consists of two distinct domains: a central domain assembled inCENP-A^{Cnp1} chromatin harbouring a central core (*cc*) DNA and flanking innermost repeats (*imr*), which are surrounded by various repetitive DNA elements known as the outer repeats (*otr-dg/dh*) assembled in H3K9me-dependent heterochromatin.[S1] In *cc2Δ::cc1* cells,[S2] 6 kb of *cc2* DNA was replaced by 5.5 kb of *cc1* sequence, allowing the specific analysis of unique *cc2* DNA present in ectopic *cc2* insertions and minichromosomes pcc2 and pHcc2. Black, red bars represent qChIP primer sites on *cc1/3* (the homologous region between *cc1* and *cc3*) and *cc2* DNA, respectively.

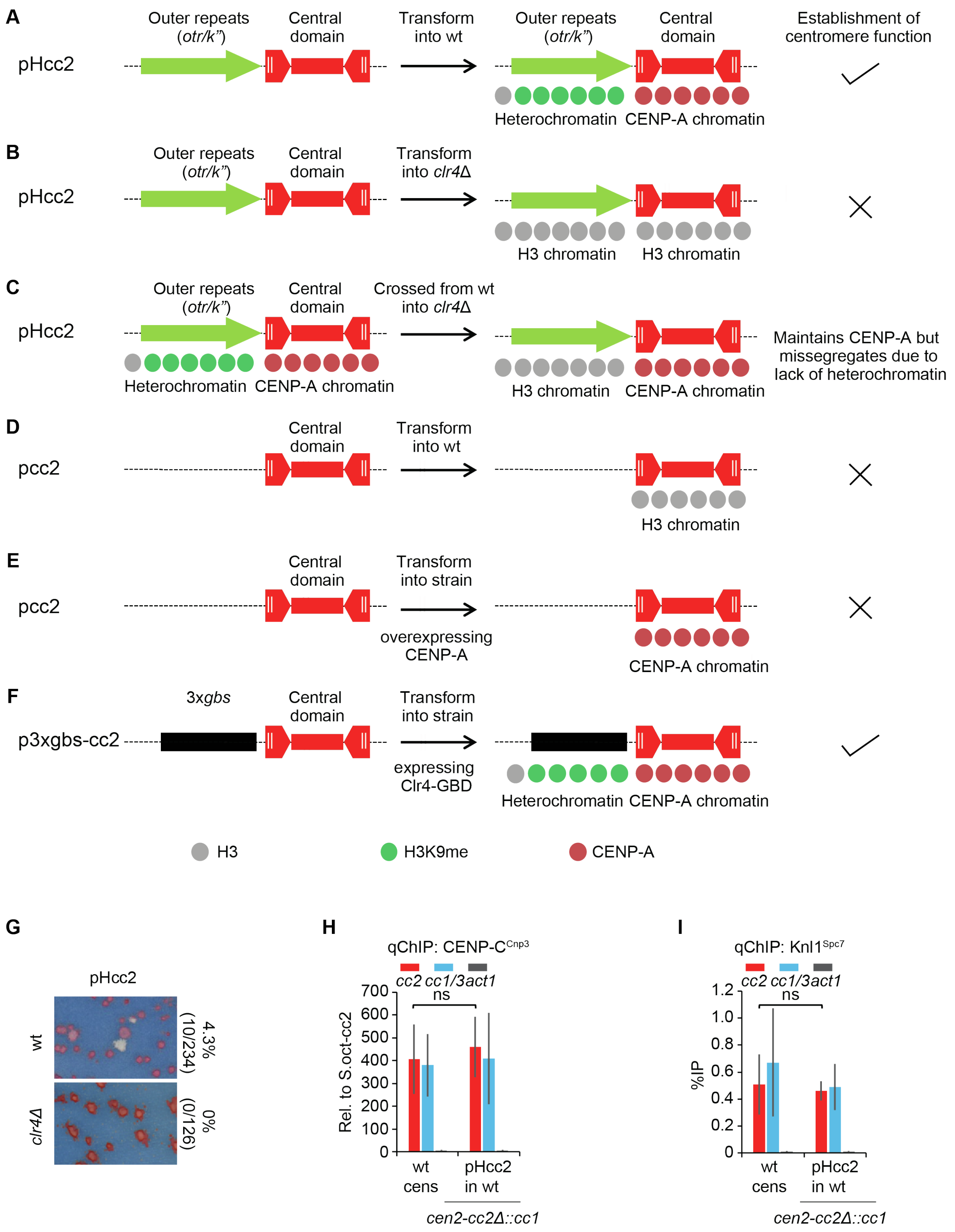

A
Outer repeats (otr/k")
Central domain
Transform into wt
Outer repeats (otr/k")
Central domain
Establishment of centromere function
pHcc2
Heterochromatin
CENP-A chromatin
B
Outer repeats (otr/k")
Central domain
Transform into clr4Δ
pHcc2
H3 chromatin
H3 chromatin
C
Outer repeats (otr/k")
Central domain
Crossed from wt into clr4Δ
pHcc2
Heterochromatin
CENP-A chromatin
H3 chromatin
CENP-A chromatin
Maintains CENP-A but missegregates due to lack of heterochromatin
D
Central domain
Transform into wt
pcc2
H3 chromatin
E
Central domain
Transform into strain overexpressing CENP-A
pcc2
CENP-A chromatin
F
3xgbs
Central domain
Transform into strain expressing Clr4-GBD
p3xgbs-cc2
Heterochromatin
CENP-A chromatin
H3
H3K9me
CENP-A
G
pHcc2
wt
4.3% (10/234)
clr4Δ
0% (0/126)
H
qChIP: CENP-C^Cnp3
cc2 cc1/3act1
ns
Rel. to S.oct-cc2
700
600
500
400
300
200
100
0
wt cens
pHcc2 in wt
cen2-cc2Δ::cc1
I
qChIP: Knl1^Spc7
cc2 cc1/3act1
ns
%IP
1.2
1.0
0.8
0.6
0.4
0.2
0
wt cens
pHcc2 in wt
cen2-cc2Δ::cc1

Figure S2. Heterochromatin is required to establish CENP-A^{Cnp1} chromatin, establish functional centromeres and recruit kinetochore proteins. Related to Figure 1.

(A, B) pHcc2 assembles heterochromatin on outer repeats (*otr/K"*) which promotes CENP-A^{Cnp1} chromatin establishment on its central domain DNA in wild-type - wt (A) cells but not in *clr4*Δ (B) cells following transformation.[S3] (C) Heterochromatin is not required to maintain CENP-A^{Cnp1} chromatin on central domain of pHcc2 in *clr4*Δ crossed from wild-type.[S3] Although pHcc2 maintains CENP-A^{Cnp1} in *clr4*Δ, it does not segregate properly due to lack of heterochromatin, cohesion and biorientation function. (D, E) pcc2 assembles CENP-A^{Cnp1} chromatin on its central domain in CENP-A^{Cnp1} overexpressed cells (E) but not in wild-type CENP-A^{Cnp1} cells (D) upon transformation.[S2] (F) p3xgbs-cc2 assembles heterochromatin on *3xgbs* (three Gal4-binding sites) that permits CENP-A^{Cnp1} chromatin establishment on its *cc2* central domain in cells expressing Clr4-GBD (the DNA binding domain of the *S. cerevisiae* Gal4 protein) fusions following transformation. Artificial association of Clr4 with *3xgbs* bypasses the requirement for heterochromatic outer repeats (*otr/K"*) in heterochromatin assembly.[S4] "✓" indicates functional centromere can be established on plasmid-based minichromosome, whereas "×" indicates no centromere is established. Both heterochromatin and CENP-A^{Cnp1} are required to form a functional centromere. (G) Functional centromere establishment assay on pHcc2 transformed into wt and *clr4Δ* strains carrying *cc2Δ::cc1*. The centromere establishment assay provides a measure of immediate ability to establish functional centromeres. pHcc2 plasmid can establish heterochromatin via the outer repeat region. Heterochromatin promotes establishment of CENP-A^{Cnp1} chromatin on the central core domain (*cc2*). CENP-A^{Cnp1} chromatin is the platform for kinetochore assembly.[S3] Heterochromatin also ensures proper cohesion, meaning that the plasmid can biorient on the spindle and segregate efficiently.[S5,S6] Both heterochromatin and CENP-A are required to form a functional centromere. For the centromere establishment assay pHcc2 was transformed into wt and *clr4Δ* strains and plated on selective plates. Transformants were promptly replica plated to low adenine-containing plates. Pale pink/white colonies indicate the presence of functional centromere since *sup3e* tRNA on pHcc2 suppresses ade6-704 mutation within strains. Establishment frequency was calculated as the percentage of pale pink/white colonies divided by the total number of transformants (minus pure white colonies) (See also STAR Methods), thus 4.3%, 0% of colonies established functional centromeres on pHcc2 transformed into wt and *clr4Δ* cells, respectively. (H, I) qChIP analyses for CENP-C^{Cnp3} (H), Knl1^{Spc7} (I) levels at *cc2, cc1/3* and *act1* in wild-type strains carrying endogenous *cen2-cc2* or *cen2-cc2*Δ*::cc1* strain transformed with pHcc2. For ChIP analyses transformants were picked randomly from selective plates. Replica plating to low adenine plates was not performed. During growth to log phase for ChIP further establishment of centromeres occurs – in up to 100% of wild-type pHcc2 cultures. %IP levels in *S. pombe* were normalized to %IP of central core from spiked-in *S. octosporus* chromatin in (H). qChIP results in (I) were reported as %IP. Data are mean ± SD (n=3). ns, no significance (Unpaired t-test).

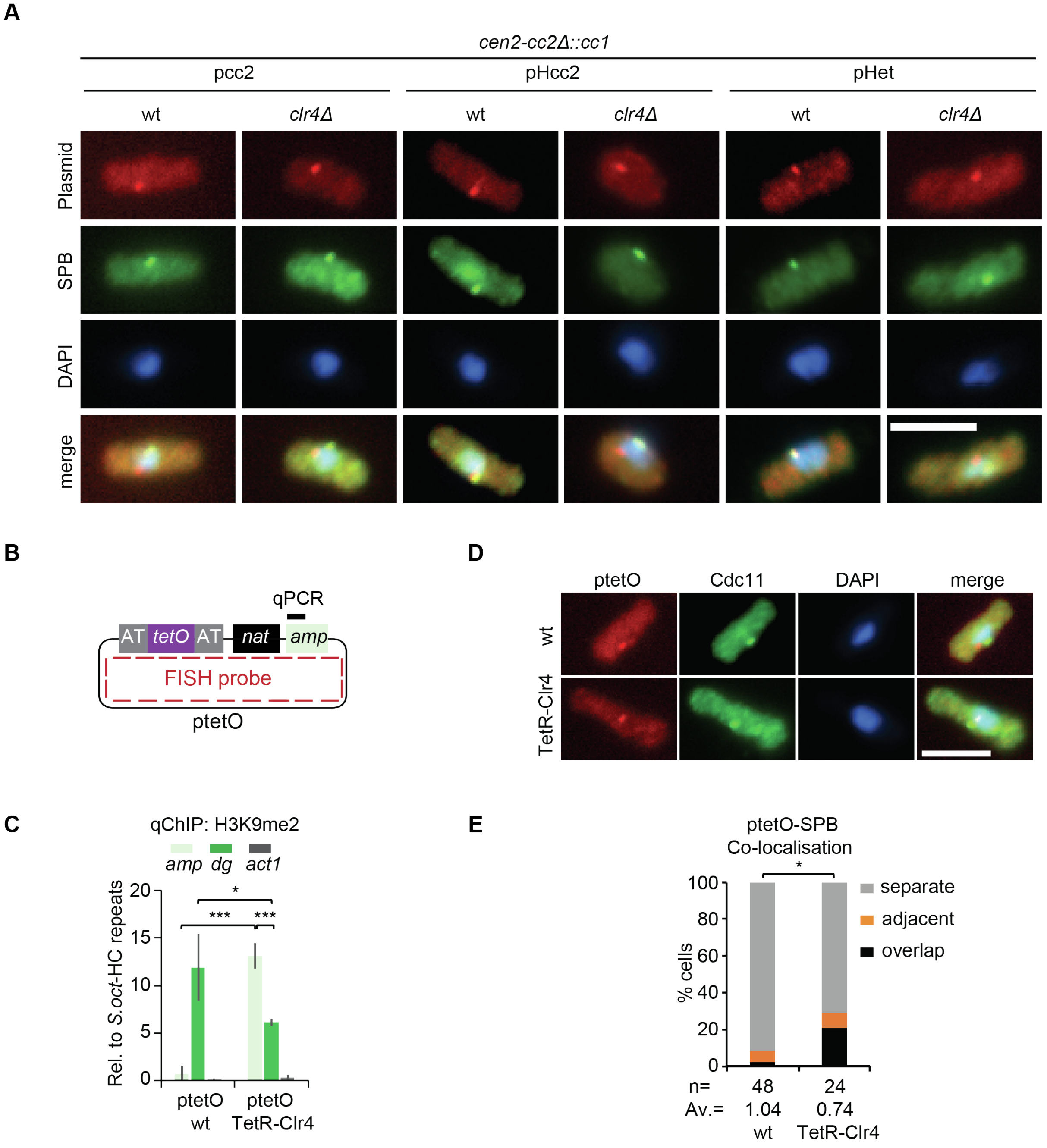

A
cen2-cc2Δ::cc1
pcc2
pHcc2
pHet
wt
clr4Δ
Plasmid
SPB
DAPI
merge
B
qPCR
AT
tetO
AT
nat
amp
FISH probe
ptetO
C
qChIP: H3K9me2
amp
dg
act1
Rel. to S.oct-HC repeats
20
15
10
5
0
*
***
***
ptetO wt
ptetO TetR-Clr4
D
ptetO
Cdc11
DAPI
merge
wt
TetR-Clr4
E
ptetO-SPB Co-localisation
*
100
80
60
40
20
0
% cells
separate
adjacent
overlap
n=
48
24
Av.=
1.04
0.74
wt
TetR-Clr4

Figure S3. The assembly of centromeric or synthetic heterochromatin increases the frequency of pHcc2, pHet and ptetO colocalization with SPBs. Related to Figure 1.

(A) Representative images of plasmid DNA FISH (red; probe as indicated in Figure 1B), SPB location (green; anti-Cdc11) and DNA staining (blue, DAPI) in wild-type (*wt*) and *clr4*Δ cells transformed with pcc2, pHcc2 or pHet. Images were scaled relative to the maximum values of histogram. Scale bar, 5 μm. (B) Diagram of ptetO minichromosome. Black bar above plasmid map represents qChIP primer site on ampicillin gene (*amp*). Dashed red line on plasmid indicates position of FISH probe. (C) qChIP analyses for H3K9me2 levels on *amp* gene of ptetO, *dg* repeats of centromeric heterochromatin and *act1* gene. (D) Representative images of ptetO plasmid DNA FISH (red; probe as indicated in A), SPB location (green; anti-Cdc11) and DNA staining (blue, DAPI) in wild-type (wt) and TetR-Clr4 cells transformed with ptetO. Images were scaled relative to the maximum values of histogram. Scale bar, 5 μm. (E) Cells were classified into three groups according to the 3D distances between ptetO and SPB (Cdc11): overlap (≤0.3 μm), adjacent (0.3-0.5 μm) or separate (0.5-3 μm). Percentage of interphase cells (n, number analyzed from 3 independent experiments) in each category. AV., average distance. ns, no significance; *, $p<0.01$ (Mann-Whitney U test).

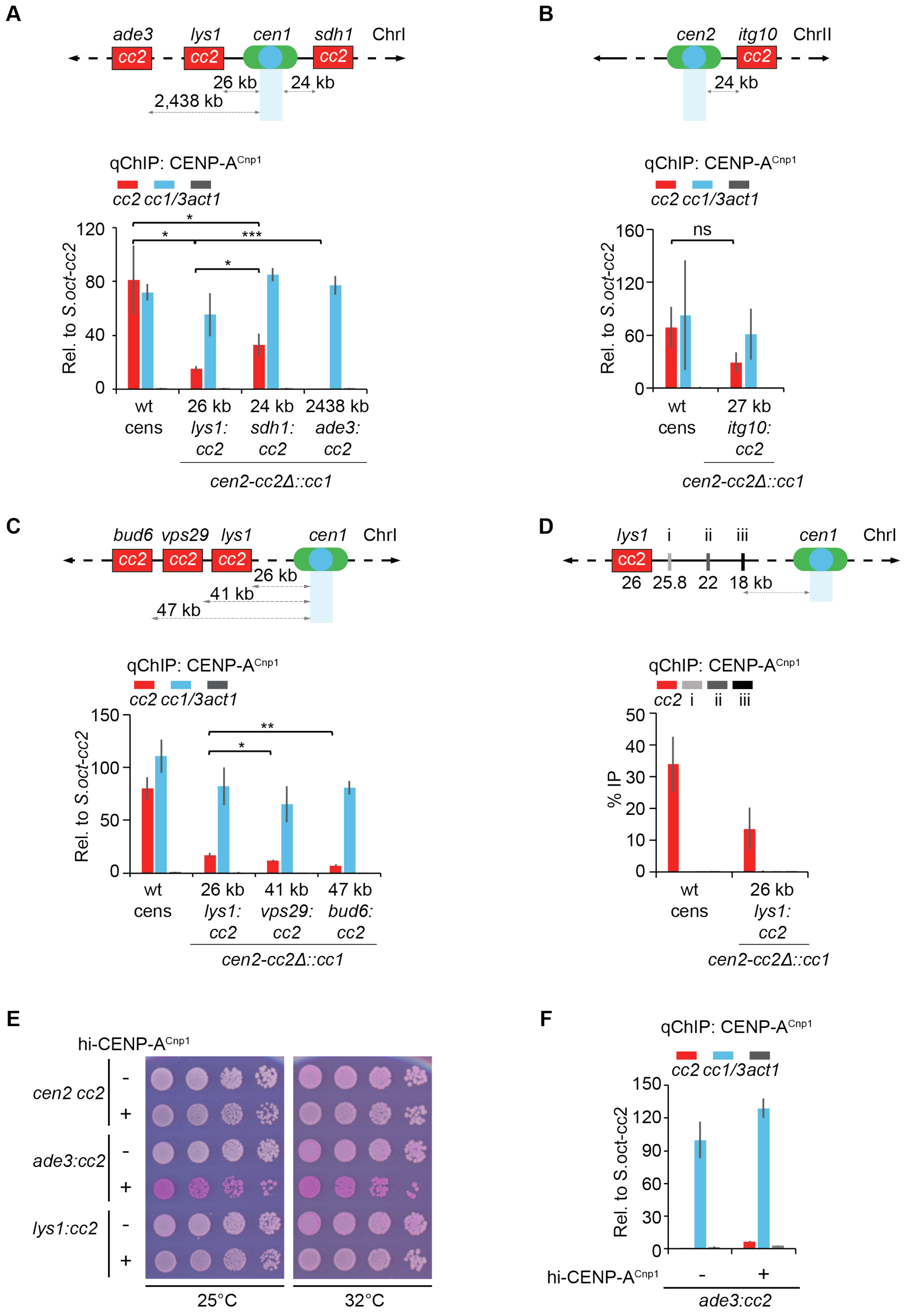

A
ade3 lys1 cen1 sdh1 ChrI
cc2 cc2 cc2
26 kb 24 kb
2,438 kb
qChIP: CENP-A Cnp1
cc2 cc1/3act1
Rel. to S.oct-cc2
120 80 40 0
wt cens
26 kb lys1: cc2
24 kb sdh1: cc2
2438 kb ade3: cc2
cen2-cc2Δ::cc1
B
cen2 itg10 ChrII
cc2
24 kb
qChIP: CENP-A Cnp1
cc2 cc1/3act1
ns
Rel. to S.oct-cc2
160 120 60 0
wt cens
27 kb itg10: cc2
cen2-cc2Δ::cc1
C
bud6 vps29 lys1 cen1 ChrI
cc2 cc2 cc2
26 kb
41 kb
47 kb
qChIP: CENP-A Cnp1
cc2 cc1/3act1
Rel. to S.oct-cc2
150 100 50 0
wt cens
26 kb lys1: cc2
41 kb vps29: cc2
47 kb bud6: cc2
cen2-cc2Δ::cc1
D
lys1 i ii iii cen1 ChrI
cc2
26 25.8 22 18 kb
qChIP: CENP-A Cnp1
cc2 i ii iii
% IP
50 40 30 20 10 0
wt cens
26 kb lys1: cc2
cen2-cc2Δ::cc1
E
hi-CENP-A Cnp1
cen2 cc2
ade3:cc2
lys1:cc2
25°C
32°C
F
qChIP: CENP-A Cnp1
cc2 cc1/3act1
Rel. to S.oct-cc2
150 120 90 60 30 0
hi-CENP-A Cnp1
ade3:cc2

Figure S4. CENP-A^{Cnp1} chromatin is established on *cc2* inserted closed to centromeres and CENP-A^{Cnp1} overexpression results in defect growth of cells containing a centromere-distal *ade3:cc2* insertion. Related to Figure 2.

(A-C) qChIP analyses for CENP-A^{Cnp1} levels at *cc2, cc1/3* and *act1* in wt-cens strain with *cen2-cc2* or *cc2Δ::cc1* strain with *lys1:cc2, sdh1:cc2*, *ade3:cc2* (A) or *itg10:cc2* (*itg10*; ChrII: 1,645,855-1,655,523; B) or *vps29:cc2* or *bud6:cc2* (C) insertion. %IP levels in *S. pombe* were normalized to %IP of central core from spiked-in *S. octosporus* chromatin. (D) qChIP analyses for CENP-A^{Cnp1} levels at three euchromatic locus between *lys1* and *cen1*: sites i, ii, iii, 25.8, 22, 18 kb from *cc1* respectively in wt-cens or *cc2Δ::cc1* strain with *lys1:cc2.* qChIP results were reported as %IP. All qChIP data are mean ± SD (n=3). ns, no significance; *, $p<0.05$; **, $p<0.005$; ***, $p<0.0005$ (Unpaired t-test). (E) Overexpression of CENP-A^{Cnp1} leads to growth defect in cells containing centromere-distant *ade3:cc2* insertion**.** Growth of indicated strains carrying endogenous *cen2 cc2* or *ade3:cc2* or *lys1:cc2* expressing wild-type levels (-; wt-CENP-A^{Cnp1}) or high levels of CENP-A^{Cnp1} (+; hi-CENP-A^{Cnp1}) on plates containing the vital dye phoxine B. Colonies with a higher proportion of dead cells stain darker pink. All strains were grown at 25°C or 32°C for 3-5 days and then photographed. (F) qChIP analyses for CENP-A^{Cnp1} levels at *cc2, cc1/3* and *act1* in *cc2Δ::cc1*, *nmt41*-CENP-A^{Cnp1} strain with *ade3:cc2* insertion expressing wild-type levels (-; wt-CENP-A^{Cnp1}) or high levels of CENP-A^{Cnp1} (+; hi-CENP-A^{Cnp1}). %IP levels in *S. pombe* in were normalized to %IP of central core from spiked-in *S. octosporus* chromatin. All qChIP data are mean ± SD (n=3). hi-CENP-A^{Cnp1} expression in A and B is provided by the nmt41 promoter (nmt41-CENP-A^{Cnp1} integrated in genome) under no thiamine conditions.

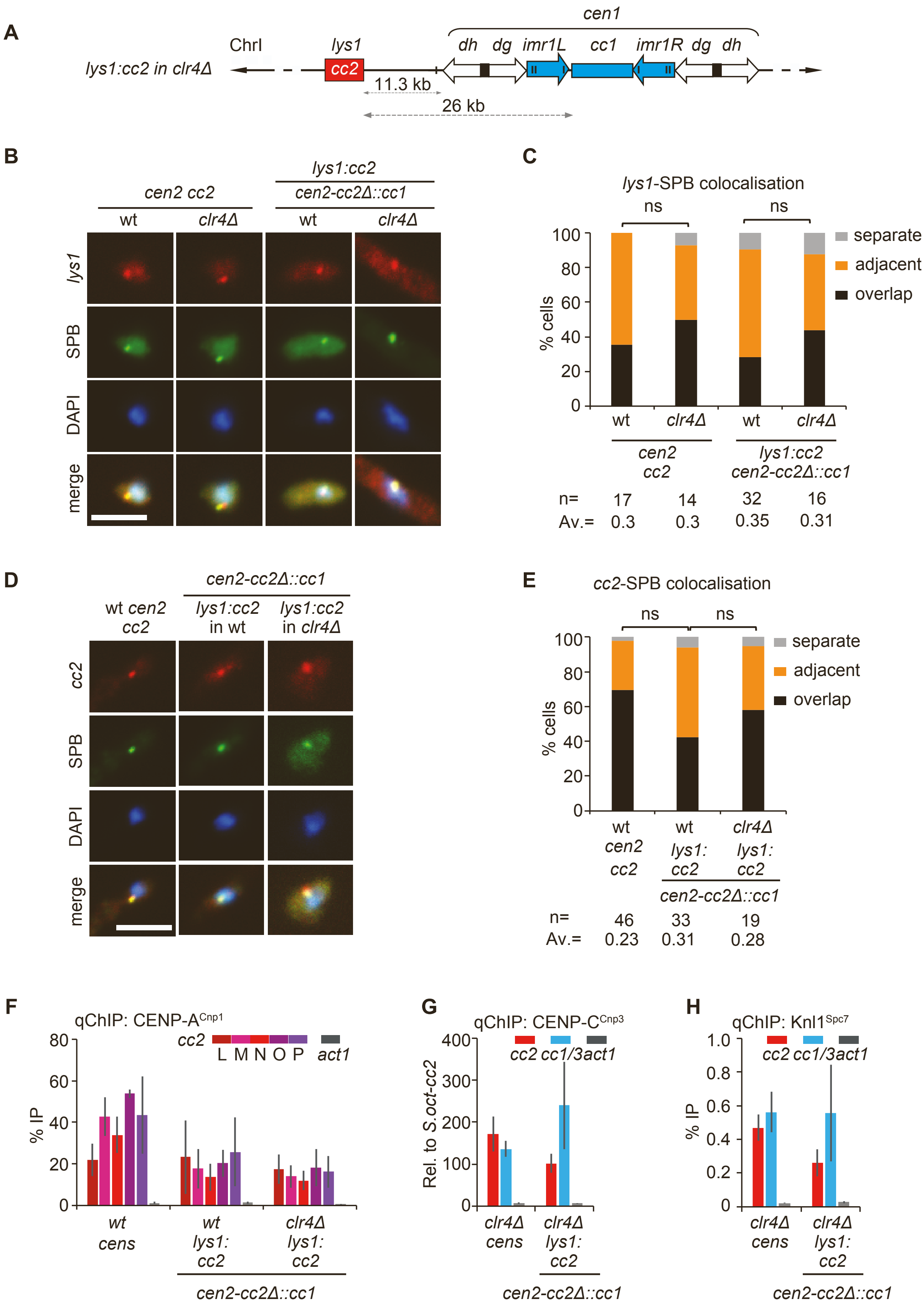

A
lys1:cc2 in clr4Δ
ChrI
lys1
cc2
cen1
dh dg imr1L cc1 imr1R dg dh
11.3 kb
26 kb
B
cen2 cc2
lys1:cc2
cen2-cc2Δ::cc1
wt clr4Δ wt clr4Δ
lys1
SPB
DAPI
merge
C
lys1-SPB colocalisation
ns ns
separate
adjacent
overlap
% cells
wt clr4Δ wt clr4Δ
cen2 cc2
lys1:cc2 cen2-cc2Δ::cc1
n= 17 14 32 16
Av.= 0.3 0.3 0.35 0.31
D
cen2-cc2Δ::cc1
wt cen2 cc2
lys1:cc2 in wt
lys1:cc2 in clr4Δ
cc2
SPB
DAPI
merge
E
cc2-SPB colocalisation
ns ns
separate
adjacent
overlap
% cells
wt cen2 cc2
wt lys1: cc2
clr4Δ lys1: cc2
cen2-cc2Δ::cc1
n= 46 33 19
Av.= 0.23 0.31 0.28
F
qChIP: CENP-A^Cnp1
cc2 L M N O P act1
% IP
wt cens
wt lys1: cc2
clr4Δ lys1: cc2
cen2-cc2Δ::cc1
G
qChIP: CENP-C^Cnp3
cc2 cc1/3 act1
Rel. to S.oct-cc2
clr4Δ cens
clr4Δ lys1: cc2
cen2-cc2Δ::cc1
H
qChIP: Knl1^Spc7
cc2 cc1/3 act1
% IP
clr4Δ cens
clr4Δ lys1: cc2
cen2-cc2Δ::cc1

Figure S5. Centromeric heterochromatin is not required to establish CENP-A^{Cnp1} chromatin on *cc2* inserted close to *cen1*. Related to Figure 2.

(A) Diagram represents *lys1:cc2* insertion, 26 kb or 11.3 kb from *cc1*, *cen1 dh* repeat in heterochromatin-deficient *clr4*Δ cells following transformation, respectively. (B, D) Representative images of *lys1* (B) or *cc2* (D) DNA FISH (red), SPB location (green; anti-Cdc11) and DNA staining (blue, DAPI) in wild-type (wt) or *clr4*Δ strain with endogenous *cen2-cc2* or *cen2-cc2*Δ*::cc1* and *lys1:cc2*. Images were scaled as in Figure 1. Scale bar, 5 μm. (C, E) Cells were classified into three groups according to the 3D distances between *lys1* (C) or *cc2* (E) and SPB (Cdc11): overlap (≤0.3 μm), adjacent (0.3-0.5 μm) or separate (0.5-3 μm). Percentage of interphase cells (n, number analyzed from 3 independent experiments) in each category. AV., average distance. ns, no significance (Mann-Whitney U test). (F-H) qChIP analyses for CENP-A^{Cnp1} (F), CENP-C^{Cnp3} (G), Knl1^{Spc7} (H) levels at *cc2*, *cc1/3* and *act1* wt or *clr4*Δ strain with endogenous *cen2-cc2* or *cen2-cc2*Δ*::cc1* and *lys1:cc2*. %IP levels in *S. pombe* were normalized to %IP of central core from spiked-in *S. octosporus* chromatin in (G). qChIP results in (F, H) were reported as %IP. Data are mean ± SD (n=3).

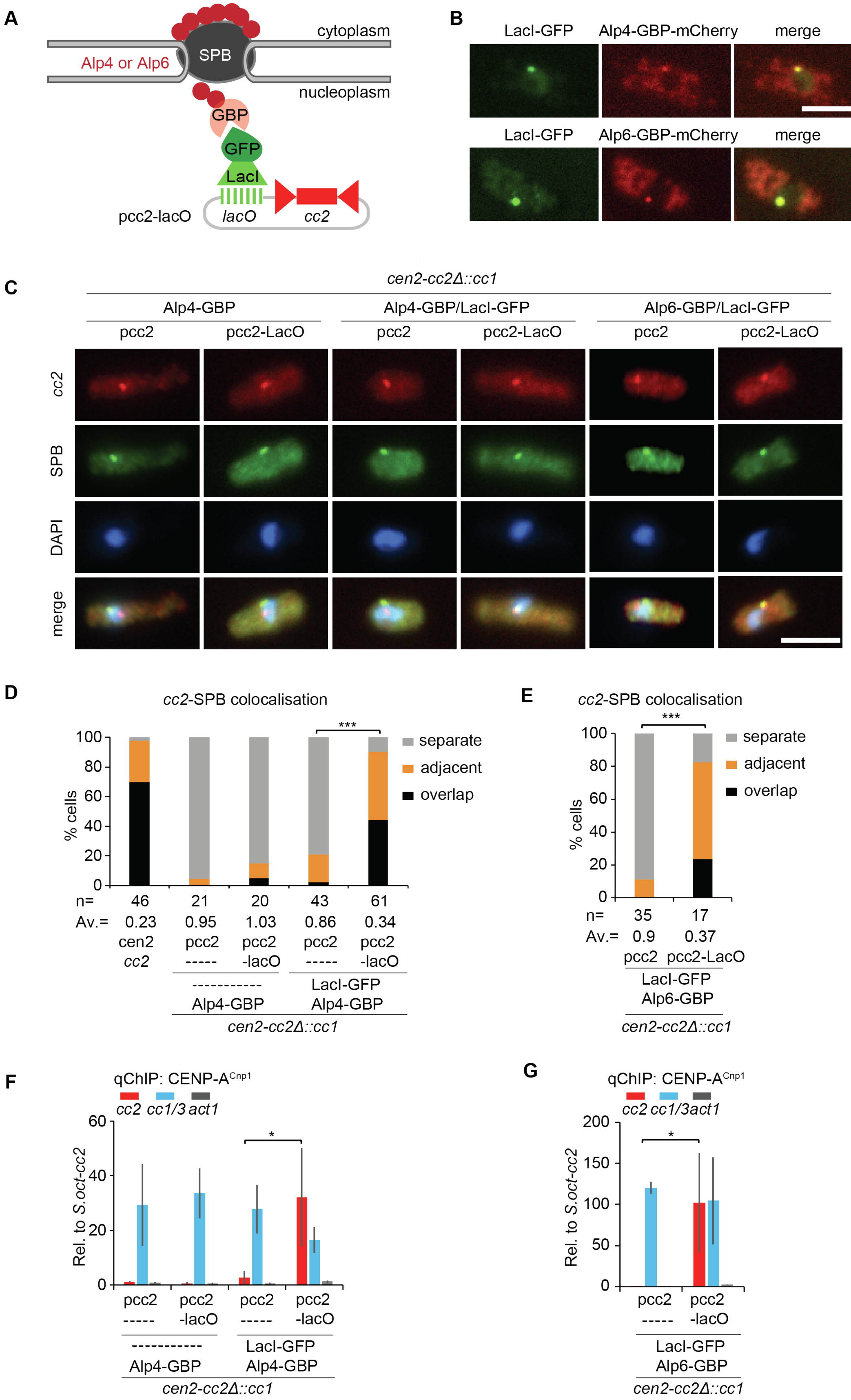
A
cytoplasm
Alp4 or Alp6
SPB
nucleoplasm
GBP
GFP
LacI
pcc2-lacO
lacO
cc2
B
LacI-GFP
Alp4-GBP-mCherry
merge
LacI-GFP
Alp6-GBP-mCherry
merge
C
cen2-cc2Δ::cc1
Alp4-GBP
Alp4-GBP/LacI-GFP
Alp6-GBP/LacI-GFP
pcc2
pcc2-LacO
pcc2
pcc2-LacO
pcc2
pcc2-LacO
cc2
SPB
DAPI
merge
D
cc2-SPB colocalisation
separate
adjacent
overlap
% cells
***
n= 46 21 20 43 61
Av.= 0.23 0.95 1.03 0.86 0.34
cen2 cc2
pcc2 -----
pcc2 -lacO
pcc2 -----
pcc2 -lacO

Alp4-GBP
LacI-GFP
Alp4-GBP
cen2-cc2Δ::cc1
E
cc2-SPB colocalisation
separate
adjacent
overlap
% cells
***
n= 35 17
Av.= 0.9 0.37
pcc2 pcc2-LacO
LacI-GFP
Alp6-GBP
cen2-cc2Δ::cc1
F
qChIP: CENP-A^Cnp1
cc2 cc1/3 act1
Rel. to S.oct-cc2
*
pcc2 -----
pcc2 -lacO
pcc2 -----
pcc2 -lacO

Alp4-GBP
LacI-GFP
Alp4-GBP
cen2-cc2Δ::cc1
G
qChIP: CENP-A^Cnp1
cc2 cc1/3 act1
Rel. to S.oct-cc2
*
pcc2 -----
pcc2 -lacO
LacI-GFP
Alp6-GBP
cen2-cc2Δ::cc1

Figure S6. Tethering *cc2* DNA to Alp4 or Alp6 allows CENP-A^{Cnp1} incorporation. Related to Figure 5.

(A) Forced association of pcc2-lacO with Alp4 or Alp6-GBP-mCherry at SPB using same tethering system as in Figure 5. A small subset of Alp4 or Alp6 molecules (red circles) are localized to the nucleoplasmic side of SPB during interphase.[S7] (B) Representative images of live cells expressing LacI-GFP and Alp4 or Alp6-GBP-mCherry. Images were scaled as in Figure 2. Scale bar, 5 μm. (C) Representative images of *cc2* DNA FISH (red), SPB location (green; anti-Cdc11) and DNA staining (blue, DAPI) in indicated strains. Images were scaled as in Figure 1. Scale bar, 5 μm. (D, E) Cells were classified into three groups according to the 3D distances between *cc2* and SPB (Cdc11): overlap (≤0.3 μm), adjacent (0.3-0.5 μm) or separate (0.5-3 μm). Percentage of interphase cells (n, number analyzed from 3 independent experiments) in each category. AV., average distance. ***, $p < 0.0001$ (Mann-Whitney U test). (F, G) qChIP analyses for CENP-A^{Cnp1} levels at *cc2*, *cc1/3* and *act1* in indicated strains. %IP levels in *S. pombe* were normalized to %IP of central core from spiked-in *S. octosporus* chromatin. Data are mean ± SD (n=3-4). *, $p<0.05$ (Unpaired t-test).

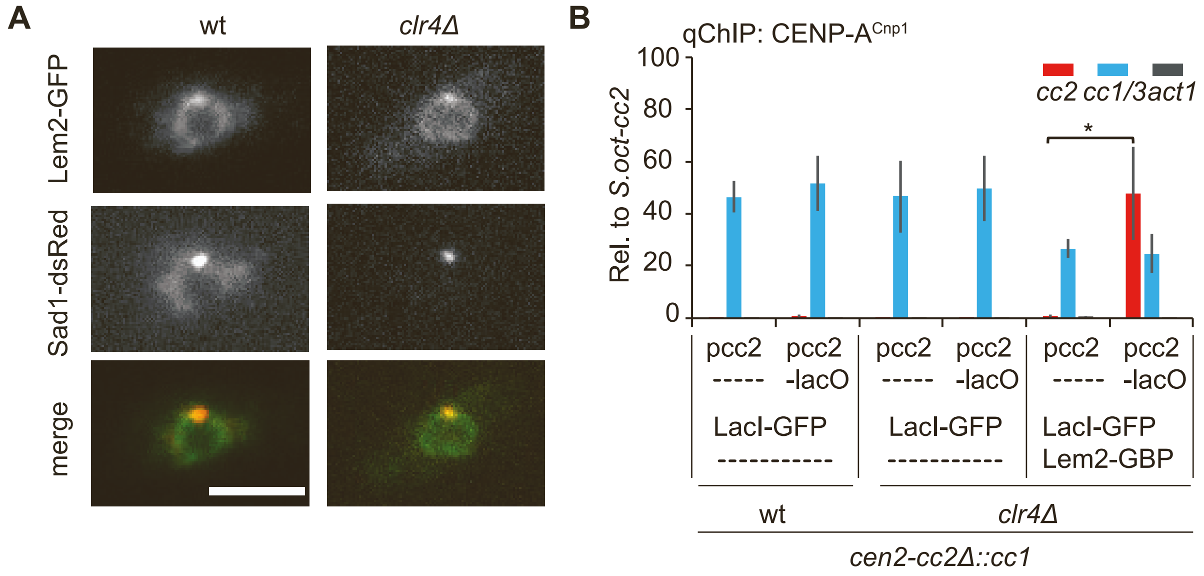

Figure S7. Tethering *cc2* DNA to Lem2 allows CENP-A^{Cnp1} incorporation independently of heterochromatin. Related to Figure 5.

(A) Representative images of live wild-type (wt) or *clr4*Δ cells expressing Lem2-GFP and Sad1-dsRed. Images were scaled as in Figure 2. Scale bar, 5 μm. (B) qChIP analyses for CENP-A^{Cnp1} levels at *cc2*, *cc1/3* and *act1* in wt or *clr4*Δ strains carrying *cen2-cc2Δ::cc1* and expressing LacI-GFP or both LacI-GFP and Lem2-GBP-mCherry transformed with pcc2 or pcc2-lacO. %IP levels in *S. pombe* were normalized to %IP of central core from spiked-in *S. octosporus* chromatin. Data are mean ± SD (n=3). *, $p<0.05$ (Unpaired t-test).

Strain number	Genotype	abbreviated as
A7374	*h+ ade6-704-HYGMX6 his3-D1 leu1-32 ura4-DSE/D18? arg3? cc2D6kb:cc1*	*wt cc2Δ::cc1*
B6596	*h+ clr4Δ ade6-704-HYGMX6 his3-D1 leu1-32 ura4-DSE/D18? arg3? cc2D6kb:cc1*	*clr4Δ*
B3665	*h+ lys1(=cen1):lacO his7+:lacIGFP, Sad1-dsRed-LEU2+*	*lys1:lacO*
B3672	*h? Sad1-dsRed-LEU2+ his7+::lacI-GFP ade3[::kanR-ura4+-lacOp] ade6-704-hygMX6 ura4-DSE/D18 leu1-32? his3-D1? arg3-D4? Lys1-131?#1*	*ade3:lacO*
A1741	*h- ade6-704-HYGMX6 his3-D1 leu1-32 ura4-DSE*	*wt cen2*
B2950	*h- lys1Δ500::cc2-KANMX6 ade6-704-HYGMX6 his3-D1 leu1-32 ura4-DSE/D18? arg3? cc2D6kb:cc1#1*	*lys1:cc2 #1* or *lys1:cc2* in wt
B2951	*h- lys1Δ500::cc2-KANMX6 ade6-704-HYGMX6 his3-D1 leu1-32 ura4-DSE/D18? arg3? cc2D6kb:cc1#2*	*lys1:cc2 #2*
B2953	*h- ade3Δ500::cc2-KANMX6 ade6-704-HYGMX6 his3-D1 leu1-32 ura4-DSE/D18? arg3? cc2D6kb:cc1#1*	*ade3:cc2 #1*
B2954	*h- ade3Δ500::cc2-KANMX6 ade6-704-HYGMX6 his3-D1 leu1-32 ura4-DSE/D18? arg3? cc2D6kb:cc1#2*	*ade3:cc2 #2*
A7373	*h- ade6-704-HYGMX6 his3-D1 leu1-32 ura4-DSE/D18? arg3? cc2D6kb:cc1*	*wt cen1* or *wt*
A9792	*h- leu1 ura4 ∆cen1::pADH1-loxP-KanR::ura4+ cd60 (neo1R)*	*cen1Δ neo1R*
B3269	*h? lys1Δ500::cc2-KANMX6 ade6-704-HYGMX6 cc2D6kb:cc1 cen1∆::pADH1-loxP-KanR::ura4+ cd60 (neo1R) his3-D1/His+? leu1-32 ura4?/ura4-DSE/D18? arg3?*	*lys1:cc2* in *neo1R*
B3260	*h- ChrI 5,435,010-5,435,237Δ226bp::cc2-KANMX6 cen1∆::pADH1-loxP-KanR::ura4+ cd60 (neo1R) ade6-704-HYGMX6 cc2D6kb:cc1 his3-D1 leu1-32 ura4-DSE/D18? arg3?*	*itg6:cc2* in *neo1R*
B3265	*h- ChrI5,447,816-5,448,235Δ400::cc2-KANMX6 cen1∆::pADH1-loxP-KanR::ura4+ cd60 (neo1R) ade6-704-HYGMX6 cc2D6kb:cc1 his3-D1 leu1-32 ura4-DSE/D18? arg3?*	*itg7:cc2* in *neo1R*
B3565	*h- ChrI5,501,647-5,502,134Δ528::cc2-KANMX6 cen1∆::pADH1-loxP-KanR::ura4+ cd60 (neo1R) ade6-704-HYGMX6 cc2D6kb:cc1 his3-D1 leu1-32 ura4-DSE/D18? arg3?*	*itg8:cc2* in *neo1R*
B3561	*h- ChrI5,501,647-5,502,134Δ528::cc2-KANMX6 ade6-704-HYGMX6 his3-D1 leu1-32 ura4-DSE/D18? arg3? cc2D6kb:cc1*	*itg8:cc2* in *wt cen1*
B4295	*h+ Lem2-GFP-Nat, Sad1-dsRed-LEU2+ ade6-210 ura4-D18*	Lem2-GFP

B3437	*h+ his7+:lacI-GFP cc2D6kb:cc1 ura4-DSE/D18? leu1-32 ade6-DNN arg3? his3+*	LacI-GFP
B4246	*h+ Lem2-GBP-mcherry-Hyg Sad1-dsRed-LEU2+ cc2D6kb:cc1 ade6-210 his3-D1? leu1-32? ura4-DSE/D18? arg3?*	Lem2-GBP
B4216	*h+ Lem2-GBP-mcherry-Hyg his7+:lacI-GFP cc2D6kb:cc1 ura4-DSE/D18? leu1-32 ade6-DNN arg3? his3+*	LacI-GFP<br>Lem2-GBP
B4339	*h- Lem2-GFP-Nat Sad1-dsRed-LEU2+ Csi1::Kan cc2∆6kb:cc1 ade6-210 ura4-D18 his3-D1? leu1-32*	Lem2-GFP<br>*csi1Δ*
B4866	*h? Lem2-GBP-mcherry-Hyg, his7+:lacI-GFP csi1∆::KanMX cc2D6kb:cc1 ade6-DNN his3-D1/ his3+? leu1-32 ura4-DSE/D18? arg3?*	LacI-GFP<br>Lem2-GBP<br>*csi1Δ*
B4865	*h? Lem2-GBP-mcherry-Hyg csi1∆::KanMX cc2D6kb:cc1 ade6-DNN his3-D1/his3+? leu1-32 ura4-DSE/D18? arg3?*	Lem2-GBP<br>*csi1Δ*
B4906	*h? Sad1-dsRed-LEU2+ his7+::lacI-GFP Csi1::Kan cc2∆6kb:cc1 ade6-704-hygMX6 ura4-DSE/D18 leu1-32 his3-D1? arg3-D4?*	LacI-GFP<br>*csi1Δ*
B3633	*h+ csi1∆::KanMX cc2∆6kb:cc1 ade6-704-HYGMX6 his3-D1 leu1-32 ura4-DSE/D18? arg3?*	*csi1Δ*
B3367	*h- sdh1Δ3344::cc2-KANMX6 ade6-704-HYGMX6 his3-D1 leu1-32 ura4-DSE/D18? arg3? cc2D6kb:cc1*	*sdh1:cc2*
B3639	*h- SPBC21B10.09-10.08cΔ650::cc2-KANMX6 ade6-704-HYGMX6 his3-D1 leu1-32 ura4-DSE/D18? arg3? cc2D6kb:cc1*	*itg10:cc2*
B3211	*h- SPAC15E1.02cΔ158bp::cc2-KANMX6 ade6-704-HYGMX6 his3-D1 leu1-32 ura4-DSE/D18? arg3? cc2D6kb:cc1*	*bud6:cc2*
B3297	*h- vps29Δ154::cc2-KANMX6 ade6-704-HYGMX6 his3-D1 leu1-32 ura4-DSE/D18? arg3? cc2D6kb:cc1*	*vps29:cc2*
A7255	*h+ clr4::NAT ade6-210 his3-D1 leu1-32 ura4-D18 arg3-D4 cc2D6kb:cc1*	*clr4Δ*<br>*cc2Δ::cc1*
B3257	*h+ clr4::NAT lys1Δ500::cc2-KANMX6 ade6-210 his3-D1 leu1-32 ura4-D18 arg3-D4 cc2D6kb:cc1*	*lys1:cc2* in *clr4Δ*
8946	*h- clr4:NAT ade6-210 leu1-32 ura4-D18 arg3-D his3D*	*clr4Δ*<br>*cen2 cc2*
B5560	*h? cc2∆6kb:cc1 Alp4-GBP-mcherry-Hyg his7+:lacI-GFP leu1-32 ura4-D18/DS-E ade6-704::hphMX/ade6-DNN? his3? arg3?*	LacI-GFP<br>Alp4-GBP
B5564	*h? cc2∆6kb:cc1 Alp4-GBP-mcherry-Hyg leu1-32 ura4-D18/DS-E ade6-704::hphMX/ade6-DNN? his3? arg3?*	Alp4-GBP
B5572	*h? cc2∆6kb:cc1 Alp6-GBP-mcherry-Hyg his7+:lacI-GFP leu1-32 ura4-D18/DS-E ade6-704::hphMX/ade6-DNN? his3? arg3?*	LacI-GFP<br>Alp6-GBP
B4349	*h- lem2-GBP-mcherry-Hyg, his7+:lacI-GFP, clr4::NAT, cc2D6kb:cc1 ura4-DSE/D18? his3+/his3-D1? arg3? leu1-32 ade6-DNN/ade6?*	LacI-GFP<br>Lem2-GBP<br>*clr4Δ*
B4351	*h? his7+:lacI-GFP clr4::NAT cc2D6kb:cc1 ura4-DSE/D18? his3+/his3-D1? arg3? leu1-32 ade6-DNN/ade6?*	LacI-GFP<br>*clr4Δ*

B4343	*h- lem2-GFP-Nat Sad1-dsRed-LEU2+ clr4::hph+ ade6-210 ura4-D18 his3-D1? leu1-32*	Lem2-GFP *clr4Δ*
1646	*h- ade6-210 arg3-D4 his3-D1 leu1-32 ura4-D18*	wt
B3797	*h+ leu1+adh21-TetROFF-2xFLAG-Clr4-cdd*	TetR-Clr4
B3416	*h- ars1(MluI)::pREP41XCnp1-leu2Sc ade6-704-HYGMX6 leu1-32 arg3-D4? his3-D1 ura4-DSE/D18? cc2D6kb:cc1*	*cen2 cc2*, hi CENP-A^{Cnp1}
B6527	*h? ars1(MluI)::pREP41XCnp1-leu2Sc lys1Δ500::cc2-KANMX6, cc2D6kb:cc1 ade6-704-HYGMX6 leu1-32 arg3-D4? his3-D1 ura4-DSE/D18?*	*lys1:cc2*, hi CENP-A^{Cnp1}
B3539	*h? ade3Δ500::cc2-KANMX6 ars1(MluI)::pREP41XCnp1-leu2Sc ade6-704-HYGMX6? leu1-32 arg3-D4? his3-D1 ura4-DSE/D18? cc2D6kb:cc1*	*ade3:cc2*, hi CENP-A^{Cnp1}

Table S1. All strains used in this study. Related to STAR Methods.

Name	Plasmid features							Notes	Reference
	marker 1	marker 2	marker 3	cc2	*K"*	*lacO or tetO*	Misc.		
pMC52			*kan*					Used for ectopic *cc2* insertion and *cc2* FISH probe	This study
pMC2 (pcc2)	*ura4*	*sup3e*	*kan*	8.6 kb				Used for tethering assay	This study
pMC12 (pcc2-lacO)	*ura4*	*sup3e*	*kan*	8.6 kb		2.8 kb; ~90 *lacO* sites		Used for tethering assay	This study
pHcc2	*ura4*	*sup3e*		8.6 kb	5.6 kb			Used for mini-chromosome establishment assay	This study
pMC183 (pHet)			*nat*		2 kb			Used to check centromeric heterochromatin nuclear localization	This study
pMC1	*ura4*	*sup3e*	*kan*					Used for plasmid backbone FISH probe	This study
pLSB-Kan			*kan*				Cas9	Used to make *clr4*Δ mutant	From Torres-Garcia.[S8]
clr4-pLSB-Kan			*kan*				Cas9 & *clr4* sgRNA	Used to make *clr4*Δ mutant	This study
pFA6a-GBP-mCherry-hygMX6			*hyg*				GBP & mCherry	Used to make Lem2/Alp4/Alp6-GBP-mCherry-Hgy fusion protein	Gift from Julia Promisel Cooper.[S9]
pFA6a-GFP-NatMX6			*nat*				GFP	Used to make Lem2-GFP fusion protein	This study
pMC171 (ptetO)			*nat*			4xtetO within 2 kb of random-ized AT-rich DNA		Used for synthetic heterochromatin assembly	This study

Table S2. All plasmids used in this study, Related to STAR Methods

Supplemental references

S1. Allshire, R.C., and Ekwall, K. (2015). Epigenetic Regulation of Chromatin States in *Schizosaccharomyces pombe*. Cold Spring Harb. Perspect. Biol. *7*, a018770.

S2. Catania, S., Pidoux, A.L., and Allshire, R.C. (2015). Sequence features and transcriptional stalling within centromere DNA promote establishment of CENP-A chromatin. PLoS Genet. *11*, e1004986.

S3. Folco, H.D., Pidoux, A.L., Urano, T., and Allshire, R.C. (2008). Heterochromatin and RNAi are required to establish CENP-A chromatin at centromeres. Science *319*, 94-97.

S4. Kagansky, A., Folco, H.D., Almeida, R., Pidoux, A.L., Boukaba, A., Simmer, F., Urano, T., Hamilton, G.L., and Allshire, R.C. (2009). Synthetic heterochromatin bypasses RNAi and centromeric repeats to establish functional centromeres. Science *324*, 1716-1719.

S5. Bernard, P., Maure, J.F., Partridge, J.F., Genier, S., Javerzat, J.P., and Allshire, R.C. (2001). Requirement of heterochromatin for cohesion at centromeres. Science *294*, 2539-2542.

S6. Nonaka, N., Kitajima, T., Yokobayashi, S., Xiao, G., Yamamoto, M., Grewal, S.I., and Watanabe, Y. (2002). Recruitment of cohesin to heterochromatic regions by Swi6/HP1 in fission yeast. Nat. Cell Biol. *4*, 89-93.

S7. Bestul, A.J., Yu, Z., Unruh, J.R., and Jaspersen, S.L. (2017). Molecular model of fission yeast centrosome assembly determined by superresolution imaging. J. Cell Biol. *216*, 2409-2424.

S8. Torres-Garcia, S., Di Pompeo, L., Eivers, L., Gaborieau, B., White, S.A., Pidoux, A.L., Kanigowska, P., Yaseen, I., Cai, Y., and Allshire, R.C. (2020). SpEDIT: A fast and efficient CRISPR/Cas9 method for fission yeast. Wellcome Open Res. *5*, 274.

S9. Fernández-Álvarez, A., Bez, C., O'Toole, Eileen T., Morphew, M., and Cooper, Julia P. (2016). Mitotic Nuclear Envelope Breakdown and Spindle Nucleation Are Controlled by Interphase Contacts between Centromeres and the Nuclear Envelope. Dev. Cell *39*, 544-559.
