# Supplementary material for: Establishment of centromere identity is dependent on nuclear spatial organization
Source: Curr Biol. 2022 Jul 25;32(14):3121–3136.e6. doi: 10.1016/j.cub.2022.06.048 (PMC9616734; doi:10.1016/j.cub.2022.06.048)
Supplement: Document S2. Article plus supplemental information [file mmc4.pdf]

Current Biology

Article

Establishment of centromere identity is dependent on nuclear spatial organization

Graphical abstract

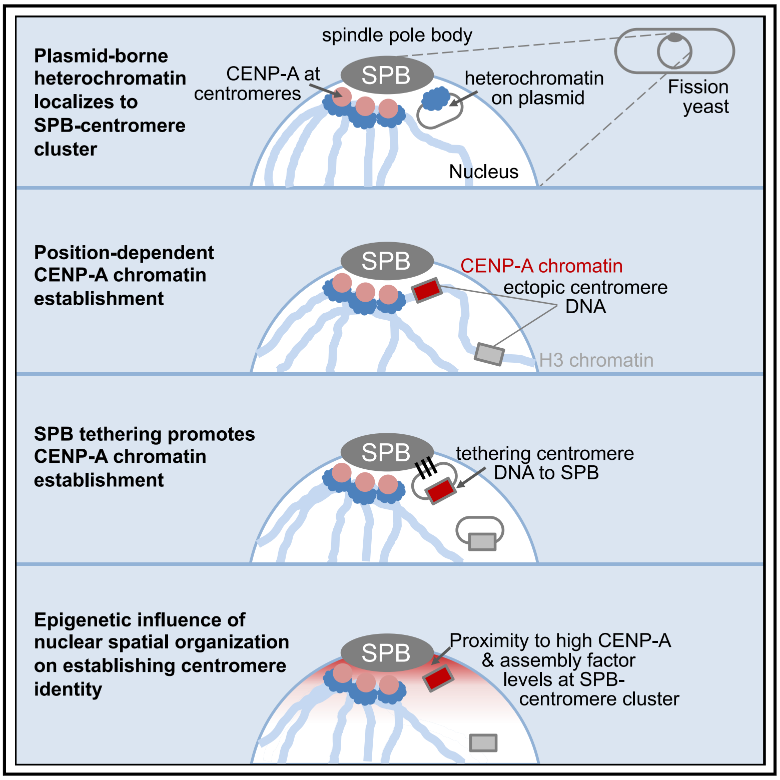

Highlights

- Episomes bearing heterochromatin localize near fission yeast SPB-centromere clusters
- Only centromere DNA inserted near active centromeres assembles CENP-A chromatin
- Centromere DNA directly tethered to the SPB assembles CENP-A chromatin
- Nuclear position is an epigenetic factor that influences centromere identity

Authors

Weifang Wu, Toni McHugh, David A. Kelly, Alison L. Pidoux, Robin C. Allshire

Correspondence

robin.allshire@ed.ac.uk

In brief

Wu et al. show that heterochromatin tends to associate with fission yeast spindle-pole bodies (SPBs) where centromeres cluster. The requirement for heterochromatin in CENP-A/kinetochore establishment on centromeric DNA is bypassed by placing or tethering centromeric DNA near SPB-centromere clusters. Thus, nuclear positioning influences centromere identity.

Wu et al., 2022, Current Biology *32*, 3121–3136
July 25, 2022 © 2022 The Author(s). Published by Elsevier Inc.
https://doi.org/10.1016/j.cub.2022.06.048

CellPress

Current Biology

CellPress
OPEN ACCESS

Article

Establishment of centromere identity is dependent on nuclear spatial organization

Weifang Wu,[1] Toni McHugh,[1] David A. Kelly,[1] Alison L. Pidoux,[1] and Robin C. Allshire[1,3,4,*]
[1]Wellcome Trust Centre for Cell Biology, Institute of Cell Biology, School of Biological Sciences, The University of Edinburgh, Edinburgh EH9 3BF, Scotland, UK
[3]Twitter: @Allshire_Lab
[4]Lead contact
*Correspondence: robin.allshire@ed.ac.uk
https://doi.org/10.1016/j.cub.2022.06.048

SUMMARY

The establishment of centromere-specific CENP-A chromatin is influenced by epigenetic and genetic processes. Central domain sequences from fission yeast centromeres are preferred substrates for CENP-A^{Cnp1} incorporation, but their use is context dependent, requiring adjacent heterochromatin. CENP-A^{Cnp1} overexpression bypasses heterochromatin dependency, suggesting that heterochromatin ensures exposure to conditions or locations permissive for CENP-A^{Cnp1} assembly. Centromeres cluster around spindle-pole bodies (SPBs). We show that heterochromatin-bearing minichromosomes localize close to SPBs, consistent with this location promoting CENP-A^{Cnp1} incorporation. We demonstrate that heterochromatin-independent *de novo* CENP-A^{Cnp1} chromatin assembly occurs when central domain DNA is placed near, but not far from, endogenous centromeres or neocentromeres. Moreover, direct tethering of central domain DNA at SPBs permits CENP-A^{Cnp1} assembly, suggesting that the nuclear compartment surrounding SPBs is permissive for CENP-A^{Cnp1} incorporation because target sequences are exposed to high levels of CENP-A^{Cnp1} and associated assembly factors. Thus, nuclear spatial organization is a key epigenetic factor that influences centromere identity.

INTRODUCTION

Centromeres are specialized chromosomal sites where multiprotein complexes known as kinetochores are assembled. Kinetochores attach chromosomes to spindle microtubules to mediate accurate mitotic and meiotic chromosome segregation. The assembly of kinetochores in many eukaryotes, including yeasts and humans, relies on specialized centromeric chromatin in which canonical histone H3 is replaced by the CENP-A/cenH3 histone H3 variant (Cnp1 in fission yeast, *Schizosaccharomyces pombe*). CENP-A-containing chromatin provides the underlying epigenetic mark that specifies the chromosomal site at which kinetochores assemble. CENP-A is required to establish and maintain centromere identity and thus indicates active centromeres.[1,2]

In organisms with monocentric chromosomes, centromeres are confined to a single locus on each chromosome. Such centromeres are often composed of long tandem arrays of repetitive sequences, such as α-satellite repeats on human chromosomes.[3] These repeats provide a substrate for the *de novo* establishment of CENP-A chromatin and the assembly of functional kinetochores when introduced into human cells. Thus, α-satellite repeats trigger centromere formation. Acentric chromosomes lacking centromeres are unable to attach to spindle microtubules and are lost during cell division. However, following centromere ablation through centromere inactivation or deletion of centromere DNA, neocentromeres can arise spontaneously at unusual locations that lack sequence similarity to normal centromere DNA but allow stable segregation of such acentric chromosomes.[3,4] Thus, centromeric DNAs are not the only sequences that can trigger the assembly of functional kinetochores. Once assembled at a particular location, including neocentromeres or sites that do not usually incorporate CENP-A, CENP-A chromatin is stably propagated at that site though cell division using intrinsic maintenance mechanisms.[5,6] Consequently, prior CENP-A assembly can mark a chromosomal locus for continued persistence of CENP-A chromatin on one homolog, whereas the same locus remains devoid of CENP-A on the other.[3]

The fission yeast genome is carried on three monocentric chromosomes with regional centromeres of 40–110 kb comprising two distinct domains (Figure S1): CENP-A^{Cnp1} chromatin assembles across the central domain consisting of central core (*cc*) and flanking innermost repeat (*imr*) DNA, which are surrounded by outer repeats (*otr-dg/dh*) assembled in Clr4 histone H3 lysine 9 methyl-(H3K9me)-transferase-dependent heterochromatin.[7,8] The central core of centromere 2 (*cc2*) is unique, but the central cores of *cen1* and *cen3* share the same sequence. *imr* elements are unique to each centromere and mark the transition between CENP-A^{Cnp1} chromatin and the heterochromatic *otr-dg/dh* repeats, which are conserved in sequence, but not in arrangement, between the three centromeres.[9,10] tRNA genes that reside in each *imr* element demarcate these distinct centromeric domains and prevent heterochromatin from encroaching into the central CENP-A^{Cnp1}

This is an open access article under the CC BY license (http://creativecommons.org/licenses/by/4.0/).

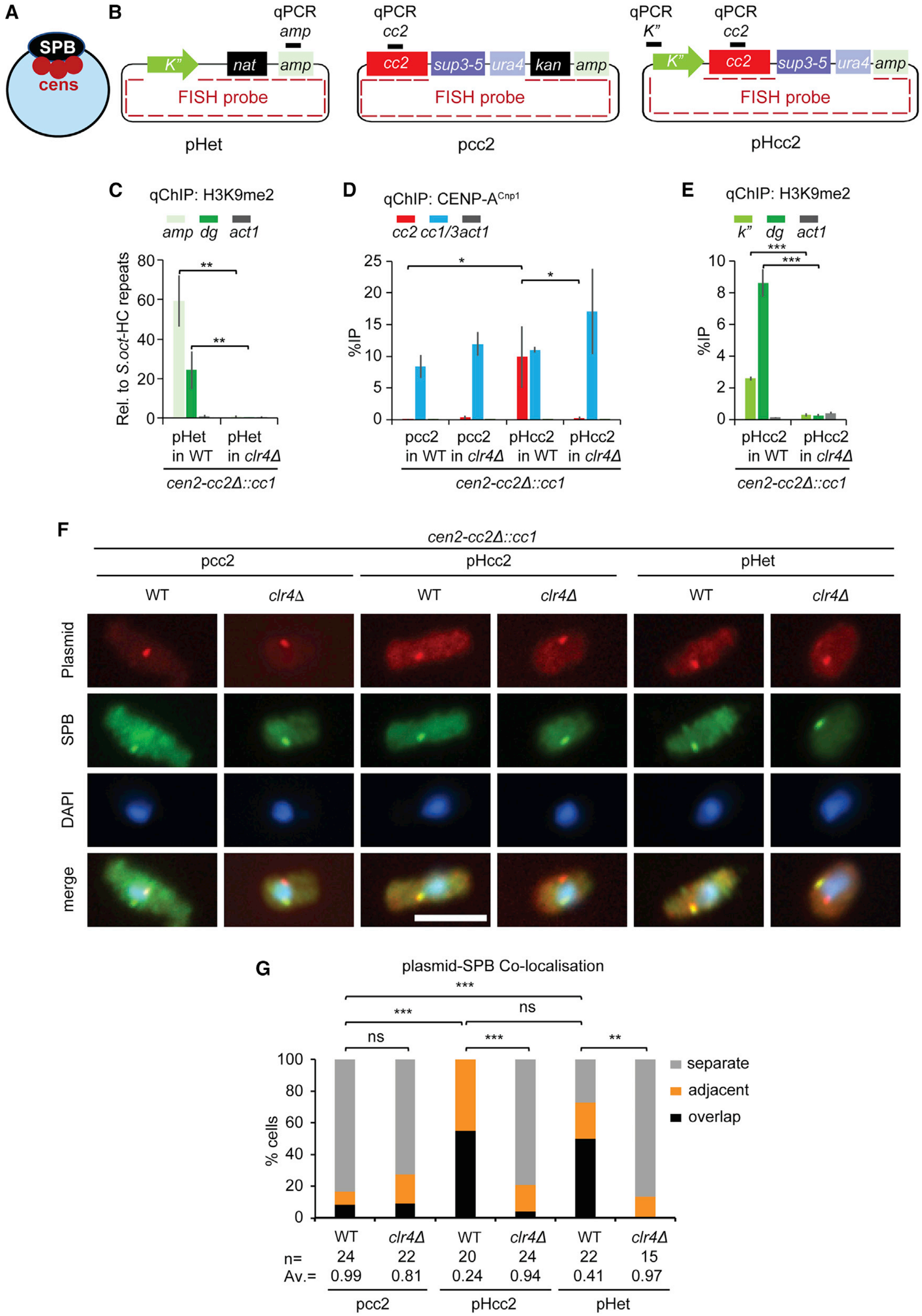

(legend on next page)

chromatin domain.[11,12] Two divergent *Schizosaccharomyces* species (*S. octosporus* and *S. cryophilus*) share a similar centromere domain organization.[13]

Like human α-satellite centromeric DNA, fission yeast central domain DNA is a preferred substrate for CENP-A^{Cnp1} and kinetochore assembly. This preferred status is underscored by the observation that, in contrast to other sequences, naive central domain DNA borne on minichromosomes readily assembles and maintains CENP-A^{Cnp1} chromatin following transient CENP-A^{Cnp1} overexpression, bypassing the usual requirement for adjacent heterochromatin.[14,15] Interestingly, despite having no sequence homology with *S. pombe* centromeres, central domains from *S. octosporus* and *S. cryophilus* are competent to assemble CENP-A^{Cnp1} chromatin and functional centromeres in *S. pombe*, indicating that fission yeast central domains possess conserved instructive features.[13] *S. pombe* central domain sequences are transcribed by RNAPII and exhibit high rates of histone H3 turnover, which may contribute to the replacement of S-phase-deposited placeholder H3 with CENP-A^{Cnp1} during the subsequent G2.[16,17] H3 is evicted from central domain chromatin even in the absence of CENP-A and kinetochore proteins.[16] The Mis18 complex acts in concert with the CENP-A chaperone, HJURP, to recognize pre-existing CENP-A nucleosomes and ensure their persistence at particular locations by mediating H3 replacement with CENP-A in new H3-containing nucleosomes assembled during the preceding S phase.[5,6,18] Thus, fission yeast central domain DNA possesses innate sequence-driven properties that program H3 eviction, making it a favored substrate for CENP-A^{Cnp1} chromatin assembly, which, once assembled, is rendered heritable though an intricate read-write mechanism.

Centromeres are tightly clustered around spindle-pole bodies (SPBs; centrosome equivalents; Figure 1A) during interphase in both fission (*S. pombe*) and budding (*Saccharomyces cerevisiae*) yeast.[19–21] In *S. cerevisiae*, SPB-to-centromere microtubules persist in G1 and mediate SPB-centromere clustering.[21–23] Proper centromere clustering around *S. pombe* SPBs is dependent on the functions of SPB component Sad1 (LINC complex SUN domain protein) and Lem2 (LEM domain inner nuclear membrane protein), which is distributed around the entire nuclear envelope (NE) but is concentrated at SPBs.[24,25] Csi1 that resides at the kinetochore-SPB interface is required for Lem2 accumulation around SPBs, and it acts with Lem2 to maintain SPB-centromere associations.[24,26,27] The CENP-A assembly factors Scm3^{HJURP}, Mis16$^{RbAP46/48}$, Mis18, and Eic1/Mis19 are concentrated at centromeres clustered close to SPBs from late anaphase to prophase, including during G2 when new CENP-A^{Cnp1} is incorporated.[16,28–31]

Although fission yeast centromeric central domains are the preferred substrate for CENP-A^{Cnp1} assembly, the establishment of CENP-A^{Cnp1} chromatin is subject to epigenetic regulation. The *de novo* assembly of CENP-A^{Cnp1} chromatin and functional centromeres on central domain sequences is dependent on the presence of adjacent outer repeat heterochromatin (Figure S2).[32,33] Direct transformation of naked minichromosome DNA into cells lacking heterochromatin compared with crossing minichromosomes preassembled in chromatin from wild-type (WT) cells results in a different fate: in the former, the central domain is assembled in H3 chromatin; in the latter, it is assembled in CENP-A^{Cnp1} chromatin (Figures S2B and S2C).[32] These observations indicate that both context and prior history are important for determining chromatin state. Synthetic heterochromatin, assembled by tethering the Clr4 H3K9-methyltransferase, substitutes for outer repeats in promoting CENP-A^{Cnp1} assembly on minichromosomes when placed next to central domain DNA (Figure S2F).[34] Thus, the properties of adjacent heterochromatin itself, rather than other features of outer repeat elements, are critical for *de novo* CENP-A^{Cnp1} assembly. Heterochromatin could promote the establishment of CENP-A^{Cnp1} chromatin by the recruitment of chromatin modifiers that influence turnover or other properties of histone H3 chromatin on the adjacent central domain to favor CENP-A^{Cnp1} deposition.[15,16] Alternatively, because CENP-A^{Cnp1} overexpression circumvents the need for flanking heterochromatin in such CENP-A^{Cnp1} chromatin establishment assays[15] (Figure S2), and endogenous heterochromatin domains are located at the nuclear periphery,[19,35,36] it is possible that centromeric heterochromatin places such minichromosomes at a nuclear location that encourages *de novo* CENP-A^{Cnp1} chromatin assembly. The centromere clusters at *S. pombe* SPBs would be expected to provide a compartment naturally enriched with CENP-A^{Cnp1} and its loading factors.

Here, we test whether the positioning of centromeric DNA relative to existing centromeres and/or SPBs influences *de novo* CENP-A^{Cnp1} chromatin assembly and the recruitment of kinetochore proteins. Heterochromatin-bearing plasmids localize close to SPBs, suggesting that heterochromatin may play a positioning role in promoting the establishment of CENP-A^{Cnp1} chromatin. We demonstrate that potentially functional centromeric central domain DNA does not assemble CENP-A^{Cnp1} or

Figure 1. Centromeric heterochromatin colocalizes with the SPB-centromere cluster

(A) Carton showing clustering of three endogenous centromeres (red circles) at the SPB (black oval) during interphase.

(B) Diagram of pHet, pcc2, and pHcc2 minichromosomes. Black bars above each plasmid map represent qChIP primer sites on ampicillin gene (*amp*), *cc2*, and *K″* repeats of plasmids, respectively. Dashed red line in plasmids indicates position of FISH probe.

(C–E) qChIP analyses for H3K9me2 levels (C and E) on *amp* gene of pHet (C); *K″* repeats of pHcc2 (E); *dg* repeats of centromeric HC and *act1* gene; CENP-A^{Cnp1} levels (D) on *cc2*, *cc1/3* (indicates sequences common to *cc1* and *cc3*), and *act1* in WT and *clr4*Δ cells containing *cc2*Δ::*cc1* at *cen2* transformed with pHet (C), pcc2 (D), or pHcc2 (D and E). %IP levels in *S. pombe* were normalized to %IP of *cen3* HC repeats from spiked-in *S. octosporus* chromatin in (C). qChIP results in (D) and (E) are reported as %IP. Data are mean ± SD (error bars) (n = 3–4 experimental replicates). $^{*}p < 0.05$, $^{**}p < 0.005$, $^{***}p < 0.0005$ (unpaired t test).

(F) Representative images of plasmid DNA FISH (red; probe as indicated in A), SPB location (green; anti-Cdc11), and DNA staining (blue, DAPI) in WT and *clr4*Δ cells transformed with pcc2, pHcc2, or pHet. Images were scaled relative to the maximum values of histogram. Scale bars, 5 μm.

(G) Cells were classified into three groups according to the 3D distances between plasmid and SPB (Cdc11): overlap (≤0.3 μm), adjacent (0.3–0.5 μm), or separate (0.5–3 μm).

Percentage of interphase cells (n, number analyzed from 3 independent experiments) in each category. AV, average distance; ns, no significance; $^{**}p < 0.001$, $^{***}p < 0.0001$ (Mann-Whitney U test) (see also STAR Methods and Figures S1–S3).

kinetochore proteins unless inserted close to an already functional native centromere or neocentromere. Thus, proximity to an existing centromere in *cis* on the same chromosome promotes CENP-A^{Cnp1} and kinetochore assembly. Direct tethering of naive minichromosome-borne central domain DNA to SPB-associated proteins in the absence of flanking heterochromatin revealed that proximity in *trans* to SPB-centromere clusters is also sufficient to trigger the assembly of CENP-A^{Cnp1} chromatin and recruitment of kinetochore components. Thus, we define a key role for spatial genome organization, in particular centromere clustering, and the resulting nuclear compartmentalization in determining centromere identity. Our findings reveal that centromeric heterochromatin functions to position centromeres within a nuclear compartment that ensures *de novo* CENP-A^{Cnp1} chromatin assembly.

RESULTS

Centromeric heterochromatin mediates localization near the SPB-centromere cluster

Endogenous fission yeast centromeres are clustered together at the SPB during interphase (Figure 1A).[19,37] The *de novo* assembly of CENP-A^{Cnp1} chromatin on naive centromeric central domain DNA that is freshly introduced into fission yeast as DNA by transformation on plasmid-based minichromosomes requires H3K9me-dependent heterochromatin formation on the flanking outer dg/dh (K/L) centromere repeat DNA (Figures S2A, S2B, and S2D).[32] Heterochromatin may influence CENP-A^{Cnp1} chromatin establishment through nuclear positioning cues. To test whether centromeric heterochromatin promotes localization close to SPBs, we utilized autonomously replicating minichromosomes, which are less constrained than endogenous chromosomal regions with respect to their positioning within nuclei. In all strains used, 6 kb of endogenous *cc2* was replaced with 5.5 kb of *cen1* central domain DNA (*cc2*Δ::*cc1*; Figure S1B). Thus, *cc2* DNA carried by minichromosomes are unique sequences in these strains, allowing their specific analysis by quantitative chromatin immunoprecipitation (qChIP). As a consequence of this manipulation, sequences common to WT *cc1* and *cc3* are present at all three endogenous centromeres in *cc2*Δ::*cc1* cells and provide a positive control comparator for CENP-A^{Cnp1} and kinetochore protein association. The establishment of centromere function (i.e., mitotic segregation ability) is assayed by replica plating fresh transformants to indicator plates (STAR Methods; Figure S2). Both centromeric heterochromatin (which ensures sister-centromere cohesion and hence biorientation of centromeres on the spindle)[38,39] and CENP-A^{Cnp1} chromatin (which recruits the kinetochore) are required for centromere function.[32] The establishment of CENP-A^{Cnp1} chromatin (and kinetochore protein recruitment) and the establishment of heterochromatin are assayed by qChIP on cultures grown from randomly picked transformants, as various plasmid/strain combinations are not capable of establishing full centromere function (Figures S2 and S3). The pHet minichromosome carries outer repeat DNA (*K*″, 2 kb) that is sufficient to trigger Clr4-dependent *de novo* heterochromatin formation when transformed into WT, but not *clr4*Δ, cells (Figures 1B and 1C; Table S2).[32,34] pcc2 carries 8.6 kb of *cen2* central domain DNA but lacks outer repeat DNA heterochromatin (Figure 1B; Table S2) and thus cannot assemble CENP-A^{Cnp1} chromatin or kinetochores (Figures 1D and S2D).[15,32] However, pHcc2, carrying both outer repeat and *cc2* DNA (Figure 1B; Table S2) forms heterochromatin, which permits CENP-A^{Cnp1} chromatin (Figures 1D and 1E), kinetochores, and functional centromeres to be frequently established *de novo* in WT cells following transformation (Figures S2A and S2G–S2I).[15,32]

Fluorescence *in situ* hybridization (FISH) to the backbone plasmid and/or cc2 sequences (Figure 1B) allowed pHet, pcc2, or pHcc2 minichromosome localization in WT cells relative to SPBs (Cdc11, SPB-specific centriolin ortholog). pcc2 was found at, or in close proximity to, SPBs in 17% of cells; however, the presence of a heterochromatic repeat on pHcc2 with resulting CENP-A^{Cnp1} and kinetochore assembly increased SPB colocalization to 100% (Figures 1F, 1G, and S3A). Consistent with a requirement for heterochromatin for SPB association, only low levels of pHcc2-SPB colocalization were detected in *clr4*Δ cells where heterochromatin and CENP-A^{Cnp1}/kinetochores are unable to assemble (Figures 1D–1G, S2G, and S3A).[32] Moreover, pHet, which only assembles heterochromatin, localized close to SPBs in 73% of WT cells but only 13% of *clr4*Δ cells (Figures 1C, 1F, 1G, and S3A). The assembly of synthetic heterochromatin via TetR-Clr4[40] increased colocalization of a ptetO plasmid with the SPB 3.5-fold (29% colocalization in TetR-Clr4 cells versus 8% in control cells), suggesting that heterochromatin assembled independently of outer repeat (K) sequences can also mediate localization with SPBs (Figures S3B–S3E).

Together, these data indicate that centromeric outer-repeat-induced heterochromatin is sufficient to mediate frequent colocalization with SPBs where centromeres and CENP-A^{Cnp1} assembly factors are concentrated. Thus, we propose that centromeric heterochromatin promotes exposure of adjacent *cc2* centromere DNA to this CENP-A^{Cnp1} assembly-factor-rich nuclear compartment, thereby ensuring the assembly of CENP-A^{Cnp1} chromatin and kinetochores.

Centromeric central domain DNA assembles CENP-A^{Cnp1} chromatin when inserted close to native centromeres

To test whether a nuclear compartment formed by SPB-centromere clustering might stimulate *de novo* CENP-A^{Cnp1} chromatin assembly, we inserted 8.6 kb of *cc2* DNA near or far from *cen1* and assayed for the presence of CENP-A^{Cnp1} chromatin. In all strains used, endogenous *cc2* had been replaced with *cc1* so that regions L-to-Q of the resulting 8.6-kb *cc2* insertions are unique (*cc2*Δ::*cc1*; Figure S1B). The *lys1* locus resides just 26 kb from *cc1* and 11.3 kb from the left *otr1* heterochromatin repeat, while *ade3* is a distant 2,438 kb from *cc1* (Figure 2A). Microscopy measurements demonstrated that *lys1* and *ade3* decorated with LacI-GFP on *lacO*-array insertions[41] are positioned in close proximity to or distant from SPBs, respectively, in three-dimensional nuclear space (Figures 2B and 2C). qChIP analysis showed that CENP-A^{Cnp1} was uniformly incorporated onto regions L-P across *cc2* following insertion at *lys1* (*lys1:cc2*). In contrast, no CENP-A^{Cnp1} enrichment was observed on *cc2* inserted at *ade3* (*ade3:cc2*) (Figure 2D). In addition, kinetochore proteins CENP-C^{Cnp3}, CENP-K^{Sim4}, and Knl1^{Spc7} were also recruited to *lys1:cc2* (Figures 2E–2G), indicating that

CellPress
OPEN ACCESS

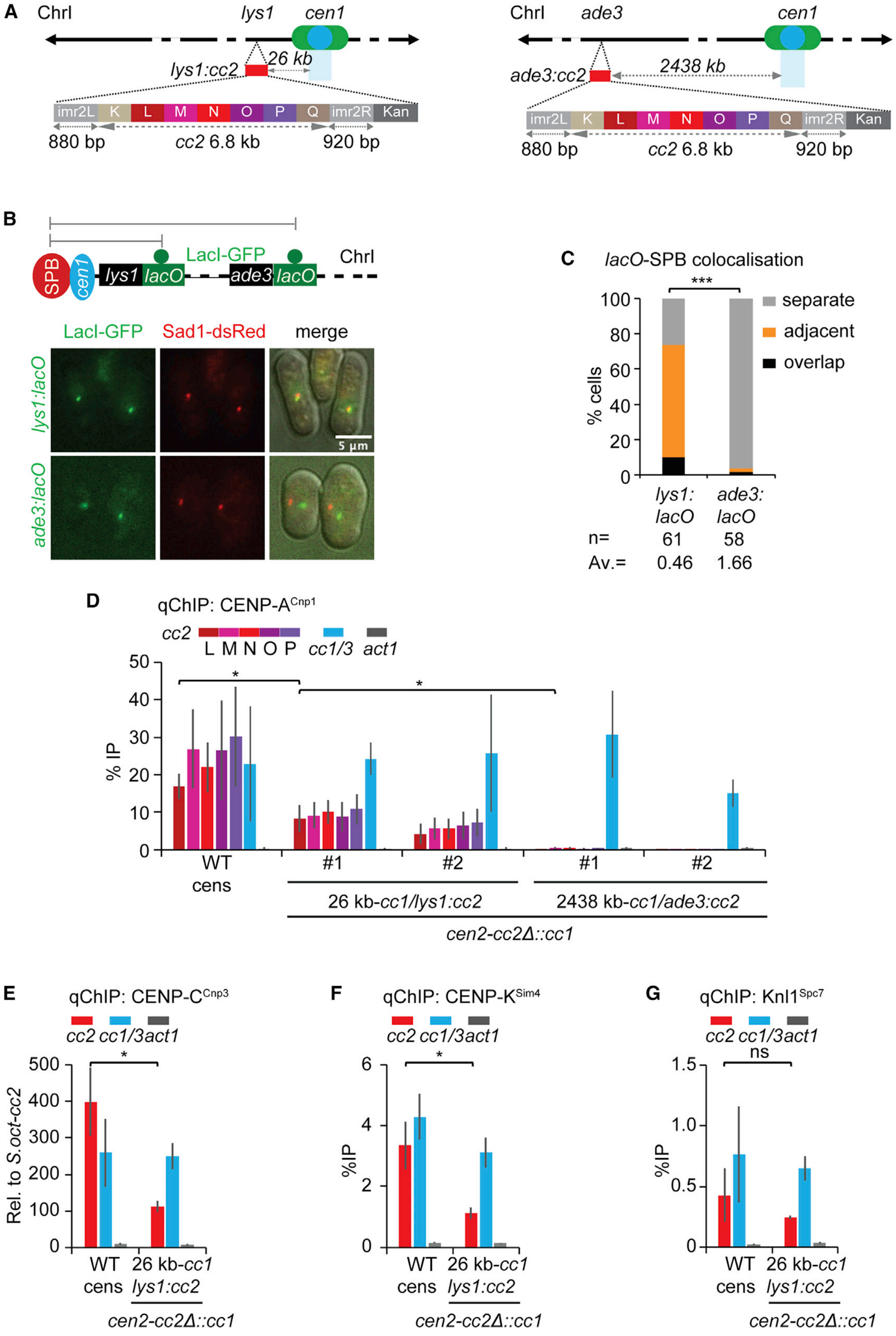

(legend on next page)

CENP-A^{Cnp1} deposition on *lys1:cc2* results in recruitment of both inner and outer kinetochore proteins. CENP-A^{Cnp1} was also incorporated on *cc2* inserted at *sdh1*, 24 kb to the right of *cen1-cc1*, or at a location we named *itg10*, 27 kb from the right side of *cen2-cc2*Δ::*cc1* (*itg10:cc2*; Figures S4A and S4B). Insertion of *cc2* at locations 41 kb (*vps29:cc2*) and 47 kb (*bud6:cc2*) that is further away on the left side of *cen1-cc1* resulted in progressively less CENP-A^{Cnp1} incorporation, suggesting that the level incorporated on inserted *cc2* DNA is dependent on its proximity in *cis* to functional *cen1* (Figure S4C). Thus, either proximity to an endogenous centromere in *cis* on the same chromosome, or exposure to a distinct nuclear compartment formed by SPB-centromere clusters, effectively mediates *de novo* CENP-A^{Cnp1} assembly and kinetochore protein recruitment on naive central domain DNA. *cc2* DNA inserted close to *cen1* might acquire CENP-A^{Cnp1} chromatin as a result of it spreading from *cen1* into *lys1:cc2*. However, little or no CENP-A^{Cnp1} enrichment was detected at three positions (i–iii) between *cen1* and *lys1:cc2* (Figure S4D). Thus, CENP-A^{Cnp1} does not uniformly spread along the chromosome from its normal location at *cen1-cc1* into the *lys1:cc2* insert.

To assess the impact of *cc2* insertions on cell viability, strains were grown on media containing the vital dye, phloxine B. Regardless of *cc2* location (*cen2-cc2*, *lys1:cc2*, or *ade3:cc2*), colonies were pale pink, indicative of normal growth (Figure S4E). In strains overexpressing *nmt41*-CENP-A^{Cnp1} (hi-CENP-A^{Cnp1}), the *ade3:cc2* centromere-distal-insertion strain was darker pink than *cen2-cc2* and centromere-proximal (*lys1:cc2*) strains, indicating decreased viability. CENP-A^{Cnp1} was detectable on *ade3:cc2* by qChIP only upon hi-CENP-A^{Cnp1}, indicating that arm-located *cc2* is competent for CENP-A^{Cnp1} incorporation under certain conditions (Figure S4F). These observations are consistent with CENP-A^{Cnp1}-overexpression-induced dicentric formation (*cen1* and centromere-proximal *ade3:cc2*) and associated reduced viability. The fact that *lys1:cc2* strains exhibit normal viability despite the incorporation of CENP-A^{Cnp1} at even endogenous levels of expression, suggests that the chromosome-bearing *lys1:cc2* is functionally monocentric due to the proximity of *lys1:cc2* to *cen1*.

These analyses demonstrate that *cc2* DNA, a known substrate for fission yeast CENP-A^{Cnp1} and kinetochore assembly, incorporates CENP-A^{Cnp1} when inserted in *cis* close to native centromeres. The finding that the levels of CENP-A^{Cnp1} incorporated decrease with increasing distance from a centromere suggests that proximity to native centromeres provides an environment that is more favorable for CENP-A^{Cnp1} and kinetochore assembly on naive centromere DNA.

Proximity to functional centromeres, not locus-specific context, promotes CENP-A^{Cnp1} chromatin establishment

Neocentromeres form near fission yeast telomeres when an endogenous centromere is deleted (Figure 3A).[4] Deletion of *cen1* (*cen1*Δ) results in neocentromeres being formed over the left (*neo1L; cd39*) or right (*neo1R; cd60*) subtelomeric regions on chromosome I.[4] FISH demonstrates that prior to neocentromere formation the subtelomeric *neo1R* locus is not located near SPBs, whereas upon CENP-A^{Cnp1} assembly and neocentromere formation, *neo1R* joins the interphase SPB-centromere cluster in 94% of cells, where CENP-A is concentrated (Figures 3A–3E). Unlike when *cc2* was inserted at *lys1* in cells with a nearby functional *cen1* (Figure 2), insertion of *cc2* at *lys1* in *cen1*Δ cells with the *neo1R* neocentromere 1.8-Mb away failed to incorporate CENP-A^{Cnp1} (Figures 4A and 4B). This finding suggests that CENP-A^{Cnp1} fails to be incorporated at *lys1:cc2* upon insertion in cells with this neocentromere because *lys1* is displaced from the centromere cluster. Thus, a prediction is that insertion of *cc2* close to a region where neocentromeres can form will only result in CENP-A^{Cnp1} incorporation when an active neocentromere is present. We therefore inserted *cc2* at locations 73 (*itg6*), 60 (*itg7*), and 7 kb (*itg8*) from the *neo1R* region in cells with a WT *cen1* (no subtelomeric neocentromere) or with an active neocentromere *neo1R* (WT *cen1* deleted) (Figure 4A).

Unlike WT cells where *itg7 and itg8* were spatially distant from SPBs, both itg7 and *itg8* were positioned close to SPB-centromere clusters in 92% and 96% of *neo1R* cells, respectively (Figures 4C–4E). CENP-A^{Cnp1} was enriched on *lys1:cc2*, but not *itg7:cc2* or *itg8:cc2*, in cells with WT *cen1* (Figure 4B). However, in cells with *cen1*Δ *neo1R*, the pattern was reversed: no CENP-A^{Cnp1} incorporation occurred on *lys1:cc2*, whereas low or high levels of CENP-A^{Cnp1} were detected on *itg7:cc2* and *itg8:cc2* that are located 60 and 8 kb from the active *neo1R* neocentromere, respectively. In addition, kinetochore protein CENP-C^{Cnp3} was recruited to *itg8:cc2* at levels comparable to sites vi and vii within *neo1R* and endogenous centromeres (Figure 4F). Little or no CENP-A^{Cnp1} was detected on the *itg6:cc2* and *itg7:cc2* insertions at greater distances from this neocentromere (Figure 4B).

In *cen1*Δ *neo1R* cells, with or without *cc2* inserted at *itg8*, we tested for CENP-A^{Cnp1} enrichment at five positions (sites i–v) between *itg8* and the active *neo1R* centromere and two positions (sites vi and vii) within *neo1R* (Figure 4A). As expected, high levels of CENP-A^{Cnp1} were detected at sites vi and vii within the characterized *neo1R* neocentromere[4] in cells with or without *cc2* inserted at *itg8*. However, substantial CENP-A^{Cnp1}

Figure 2. CENP-A^{Cnp1} chromatin is established on the centromere-adjacent *lys1:cc2* central domain

(A) Ectopic *cc2*, carrying 880 bp *imr2L*, 6.8 kb *cc2* (subdivided into K-to-Q regions; 6 kb is unique), and 920 bp *imr2R* DNA, was inserted at *lys1* (*lys1:cc2*; 26 kb from *cc1*) or *ade3* (*ade3:cc2*; 2438 kb from *cc1*) on ChrI in *cc2*Δ::*cc1* strain.

(B) Representative images of live cells expressing Sad1-dsRed (SPB marker) and LacI-GFP bound to *lys1:lacO* or *ade3:lacO*.[41] Images were scaled relative to the maximum intensity in the set of images. Scale bars, 5 μm.

(C) 3D distances between *lys1:lacO* or *ade3:lacO* and SPBs (Sad1). Percentage of G2 cells (n, number analyzed from 3 independent experiments) in each category, classified as in Figure 1. AV, average distance. ***$p < 0.0001$ (Mann-Whitney U test) (see also STAR Methods).

(D) qChIP for CENP-A^{Cnp1} at regions L-P of *cc2, cc1/3* and *act1* in WT cens strain carrying endogenous *cen2-cc2* or *cen2-cc2*Δ::*cc1* strain with *lys1:cc2* or *ade3:cc2* insertions. # number indicates individual isolates.

(E–G) qChIP analyses for CENP-C^{Cnp3} (E), CENP-K^{Sim4} (F), and Knl1^{Spc7} (G) levels at *cc2*, *cc1/3*, and *act1* genes in WT cens strain carrying endogenous *cen2-cc2* or *cen2-cc2*Δ::*cc1* strain with *lys1:cc2*. %IP levels in *S. pombe* were normalized to %IP of *S. octosporus* central core from spiked-in chromatin in (E). qChIP results in (D), (F), and (G) were reported as %IP. Data are mean ± SD (n = 3). ns, no significance; *$p < 0.05$ (unpaired t test) (see also Figures S1, S4, and S5).

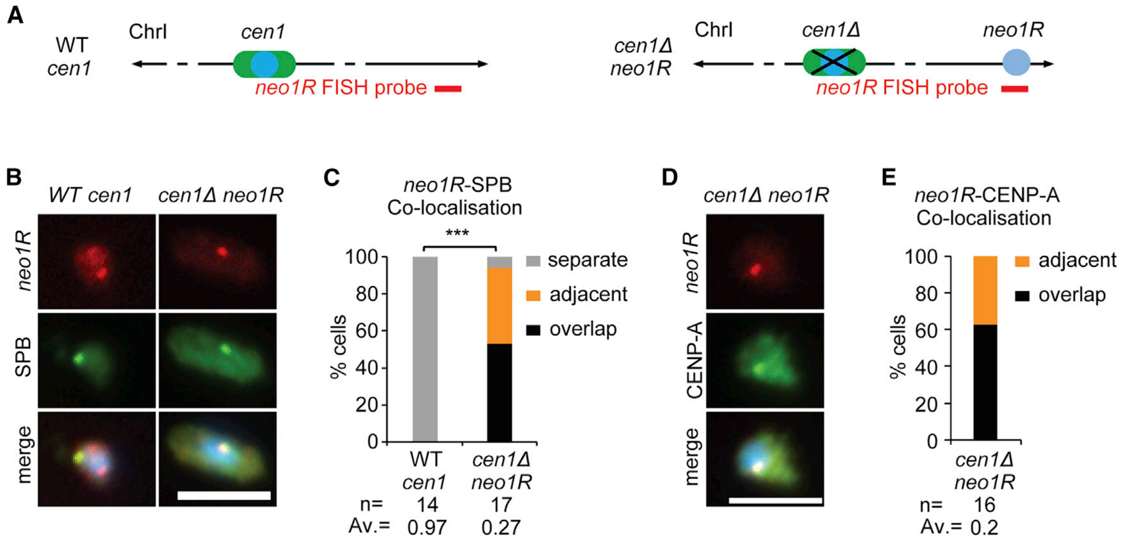

Figure 3. *neo1R* neocentromere clusters with endogenous centromeres at the SPB during interphase

(A) Diagram represents strains with *cen1* or lacking *cen1* but carrying *neo1R* neocentromere (*cen1*Δ *neo1R*). Red line indicates position of *neo1R* DNA FISH probe (ChrI: 5,513,871–5,530,124).

(B and D) Representative images of *neo1R* DNA FISH (red; probe as indicated in A), SPB location (green; anti-Cdc11; B) or centromere clusters (green; anti-CENP^{Cnp1}; D), and DNA staining (blue, DAPI) in WT cen1 (B) and *cen1*Δ *neo1R* cells. Images were scaled as in Figure 1. Scale bars, 5 μm.

(C and E) 3D distances between *neo1R* DNA and SPBs (Cdc11; C) or centromere clusters (CENP-A^{Cnp1}; E).

Percentage of interphase cells (n, number analyzed) in each distance category, classified as in Figure 1. AV, average distance. ***p < 0.0001 (Mann-Whitney U test) (see also Figure S1).

incorporation was only observed 5.2 kb from *neo1R* (site i; 1.6 kb from *itg8*, yellow bar; Figure 4G) if *cc2* was inserted at *itg8*, whereas little or no CENP-A^{Cnp1} enrichment was detected at sites i–v between *itg8* and *neo1R* in cells lacking *cc2* at *itg8* (gray bars; Figure 4G).

These analyses demonstrate that the deletion of native *cen1* prevents *de novo* CENP-A^{Cnp1} incorporation on *cc2* subsequently inserted at *lys1* but permits CENP-A^{Cnp1} assembly on *cc2* when inserted close to a resulting neocentromere. The fact that CENP-A^{Cnp1} is not detected at most positions between the *neo1R* centromere and *itg8:cc2* indicates that as at native *cen1* (Figure S4D), CENP-A^{Cnp1} chromatin does not spread uniformly from the pre-existing neocentromere to the nearby inserted *cc2* DNA. We conclude that it is the proximity of *lys1* or *itg8* to functional centromeres, rather than properties of sequences immediately flanking these loci, that allows the naturally CENP-A^{Cnp1}-permissive *cc2* DNA substrate to assemble CENP-A^{Cnp1} when inserted at these locations.

Centromeric heterochromatin is not required for *de novo* CENP-A^{Cnp1} incorporation on centromere DNA placed close to an existing centromere

In minichromosome-based establishment assays, H3K9me-dependent heterochromatin is needed to allow *de novo* CENP-A^{Cnp1} incorporation on adjacent *cc2* central domain DNA (Figure S2).[32] If the nuclear environment formed by SPB-centromere clustering is sufficient to promote *de novo* CENP-A^{Cnp1} assembly, the prediction is that centromeric heterochromatin would not be required when central domain DNA is inserted close to endogenous centromeres. The *lys1*:*cc2* insertion is positioned only 11.3 kb from endogenous *cen1* heterochromatic *dh/otr1* repeats (Figure S5A). To determine whether centromeric heterochromatin influences CENP-A^{Cnp1} chromatin establishment at *lys1*, we inserted *cc2* DNA at this locus in either WT or heterochromatin-deficient *clr4*Δ cells (lack Clr4 H3K9 methyltransferase). FISH confirmed that the *lys1* locus and *lys1*:*cc2* insertion remain near SPBs in cells lacking Clr4 (Figures S5B–S5E). qChIP demonstrated that CENP-A^{Cnp1} was established on *lys1:cc2* insertions made in either WT or *clr4*Δ cells and that both CENP-C^{Cnp3} and Knl1^{Spc7} kinetochore proteins were recruited (Figures S5F–S5H). Thus, the *de novo* assembly of CENP-A^{Cnp1} and kinetochore proteins at *lys1:cc2* occurs independently of nearby centromeric heterochromatin.

We conclude that centromeric heterochromatin is not required to assemble CENP-A^{Cnp1} and kinetochore proteins on freshly introduced centromeric DNA if that DNA is positioned in *cis* close to an existing centromere, which clusters with other centromeres and associated CENP-A^{Cnp1} plus its assembly factors, around SPBs. The placement of centromeric central domain DNA close to active centromeres bypasses the requirement for heterochromatin. This lack of a need for centromeric heterochromatin is consistent with heterochromatin normally influencing the establishment of CENP-A^{Cnp1} chromatin by sequestering freshly introduced centromeric DNA at SPBs.

Direct tethering of centromeric DNA to SPBs mediates establishment of CENP-A^{Cnp1} chromatin

Insertion of central domain *cc2* DNA near endogenous centromeres indicates that proximity in *cis* to SPB-centromere clusters enhances CENP-A^{Cnp1} chromatin establishment. If the SPB-centromere cluster creates a nuclear compartment that promotes CENP-A^{Cnp1} assembly, then positioning centromeric

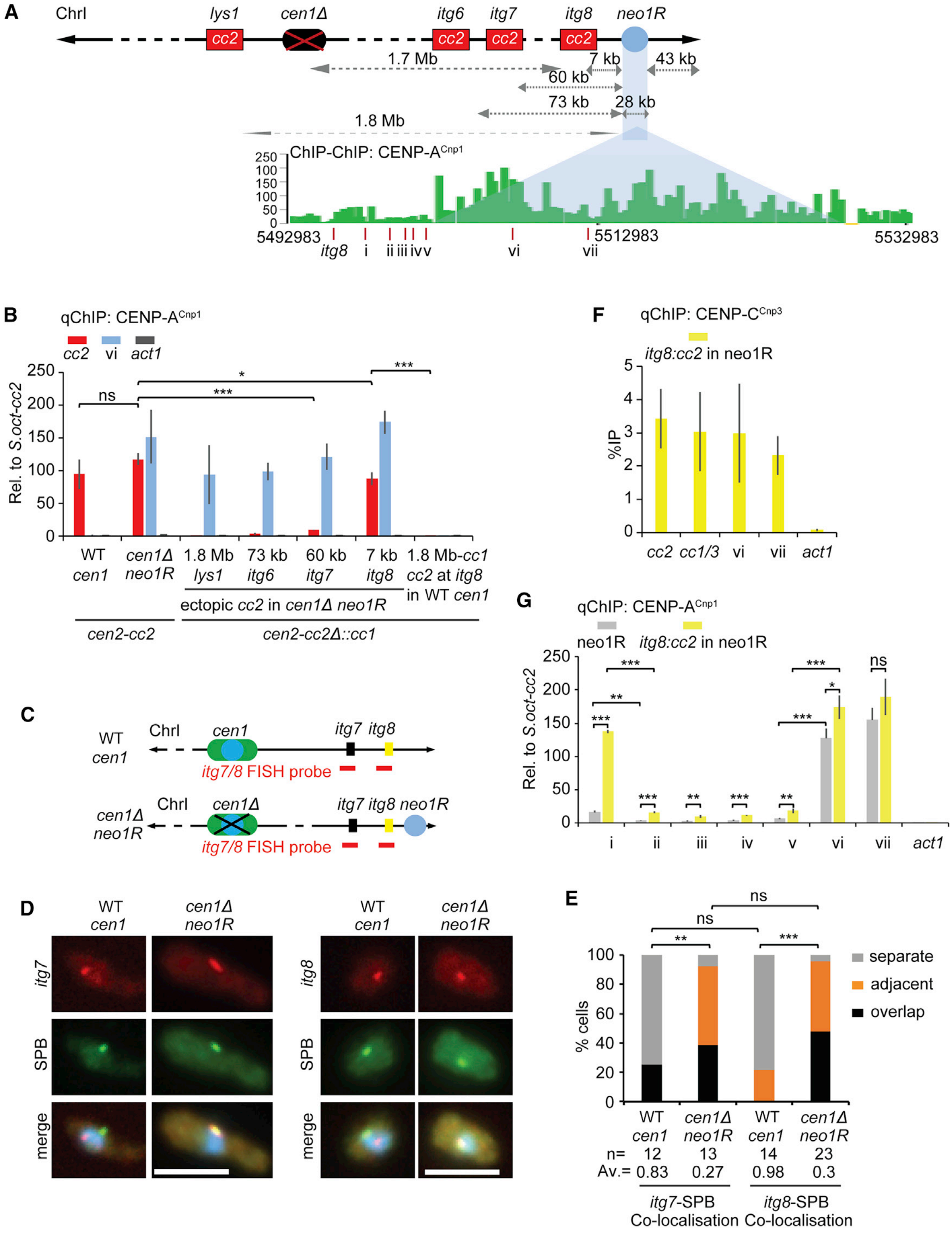

Figure 4. CENP-A^{Cnp1} chromatin can establish on central domain DNA inserted close to the neocentromere

(A) Ectopic *cc2* inserted at *lys1*, *itg6* (ChrI: 5,435,010–5,435,237), *itg7* (ChrI: 5,447,816–5,448,235), and *itg8* (ChrI: 5,501,647–5,502,134), 1.8 Mb, 73, 60, and 7 kb from *neo1R* CENP-A^{Cnp1} domain, respectively. ChIP-CHIP analysis for CENP-A^{Cnp1} in *cen1Δ neo1R* (*cd60*) strain was obtained from Ishii et al.[4] Red lines indicate *itg8* and 7 qChIP primer sites (i–vii).

(B) qChIP analyses of CENP-A^{Cnp1} levels at *cc2*, *cc1/3*, and *act1* in WT *cen1* or *cen1Δ neo1R* strain with *lys1:cc2*, *itg6:cc2*, *itg7:cc2*, or *itg8:cc2* insertions (genome positions as indicated in A).

(C) Diagram represents WT-*cen1* or *cen1Δ neo1R* strains. Red line indicates position of itg7 or *itg8* DNA FISH probe (ChrI 5,438,081–5,453,142; ChrI 5,495,975–5,508,459), respectively.

(legend continued on next page)

cc2 DNA in *trans* near SPBs might also lead to CENP-A^{Cnp1} and kinetochore assembly. To directly test whether the SPB-centromere compartment influences CENP-A^{Cnp1} chromatin establishment on centromeric DNA, we artificially tethered episomal minichromosomes to SPBs. The inner nuclear membrane (INM) protein Lem2 localizes around the NE and also exhibits strong colocalization with SPBs (Figure 5A).[24] Lem2 is also specifically enriched across the central domain of fission yeast centromeres.[26,42] Arrays of *lacO* sites (2.8 kb; ~90 *lacO* sites) were placed in pcc2, generating pcc2-lacO (Table S2), which was then transformed into cells constitutively expressing a LacI-GFP fusion protein (binds pcc2-lacO) and Lem2 fused to both GFP-binding protein (GBP) and mCherry (Lem2-GBP-mCherry; Figures 5A and 5B). Therefore, cells expressing both Lem2-GBP-mCherry and LacI-GFP should tether pcc2-lacO to SPBs. Indeed, Lem2-mediated tethering resulted in the pcc2-lacO FISH signal being in close proximity to SPBs in 77% of cells, whereas in the absence of tethering components it was located away from SPBs in >77% of cells examined (Figures 5C and 5D). Crucially, this Lem2-mediated tethering of pcc2-LacO near SPBs resulted in CENP-A^{Cnp1} incorporation at *cc2* on SPB-adjacent pcc2-lacO, whereas CENP-A^{Cnp1} was not detected on untethered pcc2-lacO or pcc2 itself (Figure 5E). In addition to CENP-A^{Cnp1}, the inner kinetochore protein, CENP-C^{Cnp3}, and outer kinetochore protein, Knl1^{Spc7}, were also assembled on the *cc2* central domain of pcc2-lacO, but only when it was tethered at SPBs (Figures 5F and 5G).

These analyses demonstrate that direct tethering of *cc2* DNA to SPBs enables CENP-A^{Cnp1} chromatin to be established without the need for adjacent heterochromatin. However, Lem2 is not an SPB-specific protein and thus Lem2-mediated pcc2-lacO tethering does not rule out the possibility that the non-SPB fraction of Lem2, localized around the NE (Figure 5A), somehow contributes to CENP-A^{Cnp1} and kinetochore protein enrichment. The Alp4 and Alp6 proteins are components of the SPB-associated γ-tubulin complex, and a proportion of both proteins localize on the nucleoplasmic side of SPBs in interphase.[43] Cells expressing Alp4-GBP-mCherry or Alp6-GBP-mCherry fusion proteins and LacI-GFP, transformed with pcc2-LacO, were therefore generated (Figures S6A and S6B). Both Alp4-GBP- and Alp6-GBP-mediated tethering resulted in pcc2-lacO being located close to SPBs in 82%–90% of cells, whereas in >79% of cells lacking tethering components, pcc2-lacO was located distant from SPBs (Figures S6C–S6E). Importantly, SPB tethering via Alp4-GBP or Alp6-GBP resulted in CENP-A^{Cnp1} incorporation on the *cc2* region of pcc2-lacO (Figures S6F and S6G). Thus, the direct tethering of *cc2* DNA to SPBs via SPB-specific components enables CENP-A^{Cnp1} chromatin establishment. The establishment of CENP-A^{Cnp1} chromatin on pcc2-lacO transformed into Lem2-GBP/LacI-GFP-expressing cells was unaffected by the absence of Clr4-dependent heterochromatin (*clr4*Δ; Figure S7).

Together, these manipulations reveal that in the absence of adjacent heterochromatin, the forced localization of centromeric central domain DNA, the native substrate for fission yeast CENP-A^{Cnp1} assembly, to SPBs is sufficient to trigger CENP-A^{Cnp1} chromatin and kinetochore assembly.

Loss of centromere-SPB association prevents CENP-A^{Cnp1} chromatin establishment

If the *de novo* establishment of CENP-A^{Cnp1} chromatin on centromeric DNA tethered near SPBs depends on the surrounding nuclear compartment, then loss of centromere-SPB association would be expected to hinder CENP-A^{Cnp1} incorporation. The accumulation of Lem2 at SPBs requires the Csi1 protein; in cells lacking Csi1 (*csi1*Δ) Lem2, Lem2-GBP-mCherry and associated LacI-GFP are mainly localized around the nuclear periphery (Figures 6A and 6C).[24] We therefore used *csi1*Δ cells to test whether the loss of the SPB-associated Lem2 pool affects Lem2-mediated tethering of pcc2-lacO at SPBs (Figures 6A and 6B). Indeed, pcc2-lacO was located near SPBs in only 23% of *csi1*Δ cells compared with 77% of WT cells expressing Lem2-GBP-mCherry and LacI-GFP (Figures 6D and 6E). Furthermore, *csi1*Δ cells were unable to establish CENP-A^{Cnp1} chromatin on Lem2-tethered pcc2-lacO (Figure 6F). However, CENP-A^{Cnp1} can assemble *de novo* on *cc2* of pHcc2 transformed into *csi1*Δ cells (Figure 6G), indicating that Csi1 itself is not required for CENP-A^{Cnp1} establishment. Thus, Lem2 needs to be concentrated at SPBs in order to induce CENP-A^{Cnp1} incorporation on tethered centromeric DNA.

Together, these data indicate that pericentromeric heterochromatin is sufficient to mediate frequent colocalization with SPBs where centromeres and CENP-A^{Cnp1} assembly factors are concentrated. We conclude that heterochromatin promotes the exposure of adjacent *cc2* centromere DNA to this CENP-A^{Cnp1} assembly-factor-rich nuclear sub-compartment, thus ensuring the assembly of CENP-A chromatin and kinetochores (Figure 7).

DISCUSSION

Assembly of CENP-A chromatin is epigenetically regulated. Here, we demonstrate that in addition to the impact of chromatin context and prior CENP-A history, spatial location within the nucleus is an epigenetic influence on the chromatin fate of centromeric DNA. We show that heterochromatin causes minichromosomes to localize near SPBs, providing a likely

(D) Representative images of *itg7* or *itg8* DNA FISH (red; probe as indicated in C), SPB location (green, anti-Cdc11), and DNA staining (blue, DAPI) in WT *cen1* and *cen1*Δ *neo1R* cells. Images scaled as in Figure 1. Scale bars, 5 μm.

(E) 3D distances between *itg7* or *itg8* and SPBs (Cdc11), percentage of interphase cells (n, number analyzed) in each category, classified as in Figure 1. AV, average distance. ns, no significance; **p < 0.001, ***p < 0.0001 (Mann-Whitney U test).

(F) qChIP analyses for CENP-C^{Cnp3} levels at *cc1/3*, *cc2*, *act1*, and site i and ii within *neo1R* in *cen1*Δ *neo1R* strain with *itg8:cc2* insertion. qChIP results were reported as %IP.

(G) qChIP analyses for CENP-A^{Cnp1} levels at 7 loci (i–vii, positions as indicated in A) and *act1* in *cen1*Δ *neo1R* strain, with or without *itg8:cc2* insertion.

ns, no significance; *p < 0.05, **p < 0.005, ***p < 0.0005 (unpaired t test). %IP levels in *S. pombe* were normalized to %IP of *S. octosporus* central core from spiked-in chromatin (B and G). Data are mean ± SD (n = 3) (see also Figure S1).

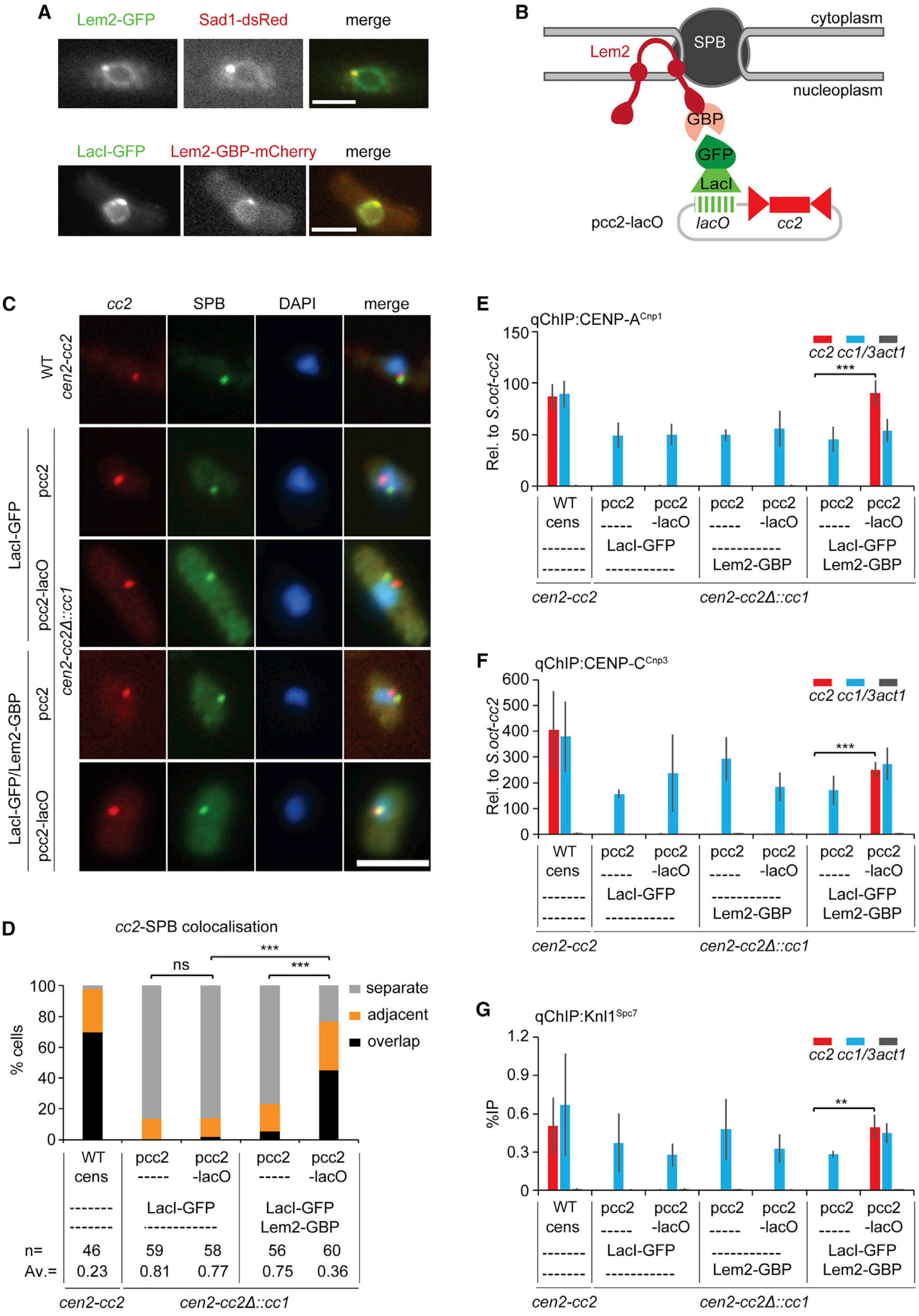

Figure 5. Tethering *cc2* DNA to Lem2 allows CENP-A^{Cnp1} incorporation and kinetochore protein recruitment

(A) Representative images of live cells expressing Lem2-GFP and Sad1-dsRed or LacI-GFP and Lem2-GBP-mCherry. Images were scaled as in Figure 2. Scale bars, 5 μm.

(legend continued on next page)

CellPress
OPEN ACCESS

explanation for the role of heterochromatin in promoting CENP-A^{Cnp1} chromatin establishment on adjacent centromeric sequences. By placing a CENP-A^{Cnp1} assembly competent sequence (*cc2*) in various spatial contexts, we demonstrate that being in the vicinity of centromere clusters at SPBs triggers *de novo* CENP-A^{Cnp1} chromatin establishment.

Despite epigenetic factors being important in the establishment of CENP-A chromatin, certain sequences are preferred, including human α-satellite arrays and fission yeast central domains. Rather than the precise sequence being critical, evidence suggests that innate properties of central domain regions, such as their unusual transcriptional landscape and high rates of histone H3 turnover, are permissive for CENP-A^{Cnp1} incorporation into chromatin.[15–17] Although central domain sequences are the preferred substrate for CENP-A^{Cnp1} assembly in fission yeast, *de novo* assembly of CENP-A^{Cnp1} chromatin is context dependent. Outer-repeat-directed or synthetic heterochromatin promotes CENP-A^{Cnp1} chromatin establishment on the adjacent central domain DNA.[32,34] CENP-A^{Cnp1} overexpression induces *de novo* CENP-A^{Cnp1} chromatin establishment on plasmid-based minichromosomes devoid of heterochromatin and carrying only central domain sequences.[15]

We have previously suggested two models to explain these observations.[15,32,44] In the "modifier" model, heterochromatin performs a chromatin-directed role such as recruitment of histone-modifying enzymes or remodelers that influence histone dynamics to favor CENP-A^{Cnp1} incorporation on adjacent central domain regions. In this scenario, CENP-A^{Cnp1} overexpression would shift the equilibrium away from transcription-dependent histone H3 recycling and toward CENP-A^{Cnp1} deposition. In the "positioning" model, the role of heterochromatin, due to its own localization, would place central domain DNA at a nuclear location permissive for CENP-A^{Cnp1} deposition, such as a compartment exhibiting high levels of CENP-A^{Cnp1} and associated assembly factors. In this model, overexpression of CENP-A^{Cnp1} would bypass heterochromatin's function by making a greater proportion of nuclear space permissive for CENP-A^{Cnp1} assembly.

Here, we have utilized FISH to demonstrate that minichromosome-borne heterochromatin preferentially locates close to SPBs. We hypothesize that any sequence positioned at this location will be exposed to high concentrations of CENP-A^{Cnp1} and its assembly factors because centromeres are clustered at SPBs for most of the cell cycle. However, only sequences such as centromeric central domain DNA, with embedded properties that drive transcription-coupled H3 replacement with CENP-A^{Cnp1}, actually incorporate CENP-A^{Cnp1}.[16]

Support for the hypothesis that a key role for heterochromatin in CENP-A^{Cnp1} establishment is to position the central domain within the SPB-centromere cluster compartment of nuclei is provided by our finding that centromeric *cc2* DNA inserted close to endogenous or neocentromeres assembled CENP-A^{Cnp1} chromatin, whereas *cc2* inserted at locations far away from centromeres did not. The positioning of *lys1* and *itg8* close to SPBs in WT and neocentromere-containing cells, respectively, correlates with the incorporation of CENP-A^{Cnp1} on *cc2* when inserted at these sites. Although the failure of CENP-A^{Cnp1} to assemble on centromere-distal sites such as *ade3* could be attributed to selection against deleterious dicentric formation on this endogenous chromosome, a strain bearing *ade3:cc2* does not show decreased viability compared with strains with *cc2* at *cen2* or *cen1*-proximal *lys1:cc2* (Figure S7). Centromere-distal *cc2* is not refractory to CENP-A^{Cnp1} incorporation, as combining CENP-A^{Cnp1} overexpresssion with *ade3:cc2* results in the incorporation of CENP-A^{Cnp1} and reduced viability, consistent with dicentric chromosome formation. In addition, we have previously shown that *cc2* present on the arm of a 530-kb non-essential linear minichromosome also does not normally assemble CENP-A^{Cnp1}. However, that minichromosome is capable of dicentric formation because overexpressed CENP-A^{Cnp1} incorporates into *cc2* and causes missegregation.[15] Thus, placing central domain DNA near centromeres in *cis* results in CENP-A^{Cnp1} incorporation. Moreover, direct tethering of minichromosome-borne central domain DNA in *trans* to SPB-associated proteins also triggered the *de novo* assembly of CENP-A^{Cnp1} chromatin, bypassing the requirement for heterochromatin. Thus, when susceptible sequences are positioned in the vicinity of SPBs, the establishment of CENP-A^{Cnp1} chromatin is uncoupled from the presence of heterochromatin. These observations indicate that nuclear positioning is an epigenetic factor that is important for establishing centromere function, and the function that heterochromatin provides is positioning information.

Our finding that centromeric central domain *cc2* DNA inserted close to an endogenous natural centromere or neocentromere results in gain of CENP-A^{Cnp1} chromatin is consistent with the centromere-SPB cluster providing a favorable microenvironment for *de novo* CENP-A^{Cnp1} and kinetochore assembly. Interestingly, it has previously been proposed that during a brief period in meiotic prophase when centromeres and telomeres colocalize at the SPB, telomeres contribute to a SPB-focused microenvironment, which promotes the incorporation of GFP-CENP-A^{Cnp1} and reinforces centromere identity in meiosis.[45]

(B) Schematic representation of the tethering system used to force pcc2-lacO association with Lem2-GBP-mCherry at the NE and SPB. pcc2-lacO is bound by LacI-GFP and ultimately tethered to Lem2-GBP-mCherry via GFP/GBP interaction.
(C) Representative images of *cc2* DNA FISH (red), SPB location (green, anti-Cdc11), and DNA staining (blue, DAPI) in WT cens strain carrying endogenous *cen2-cc2* or *cen2-cc2*Δ::*cc1* strain expressing LacI-GFP or both LacI-GFP and Lem2-GBP-mCherry transformed with pcc2 or pcc2-lacO. Fluorescence of LacI-GFP and Lem2-GBP-mCherry was dissipated by the immunofluorescence/DNA FISH procedure and did not contribute punctate signal. Images were scaled as in Figure 1. Scale bars, 5 μm.
(D) 3D distances between *cc2* and SPBs (Cdc11), percentage of interphase cells (n, number analyzed) in each category, classified as in Figure 1. AV, average distance; ns, no significance; ***$p < 0.0001$ (Mann-Whitney U test).
(E–G) qChIP analyses for CENP-A^{Cnp1} (E), CENP-C^{Cnp3} (F), and Knl1^{Spc7} (G) levels at *cc2*, *cc1/3*, and *act1* in WT cens strain carrying endogenous *cen2-cc2* or *cen2-cc2*Δ::*cc1* strain expressing LacI-GFP or Lem2-GBP-mCherry, or both of them transformed with pcc2 or pcc2-lacO. %IP levels in *S. pombe* were normalized to %IP of *S. octosporus* central core from spiked-in chromatin in (E) and (F). qChIP results in (G) reported as %IP. Data are mean ± SD (n = 3). **$p < 0.005$, ***$p < 0.0005$ (unpaired t test) (see also Figures S1, S6, and S7).

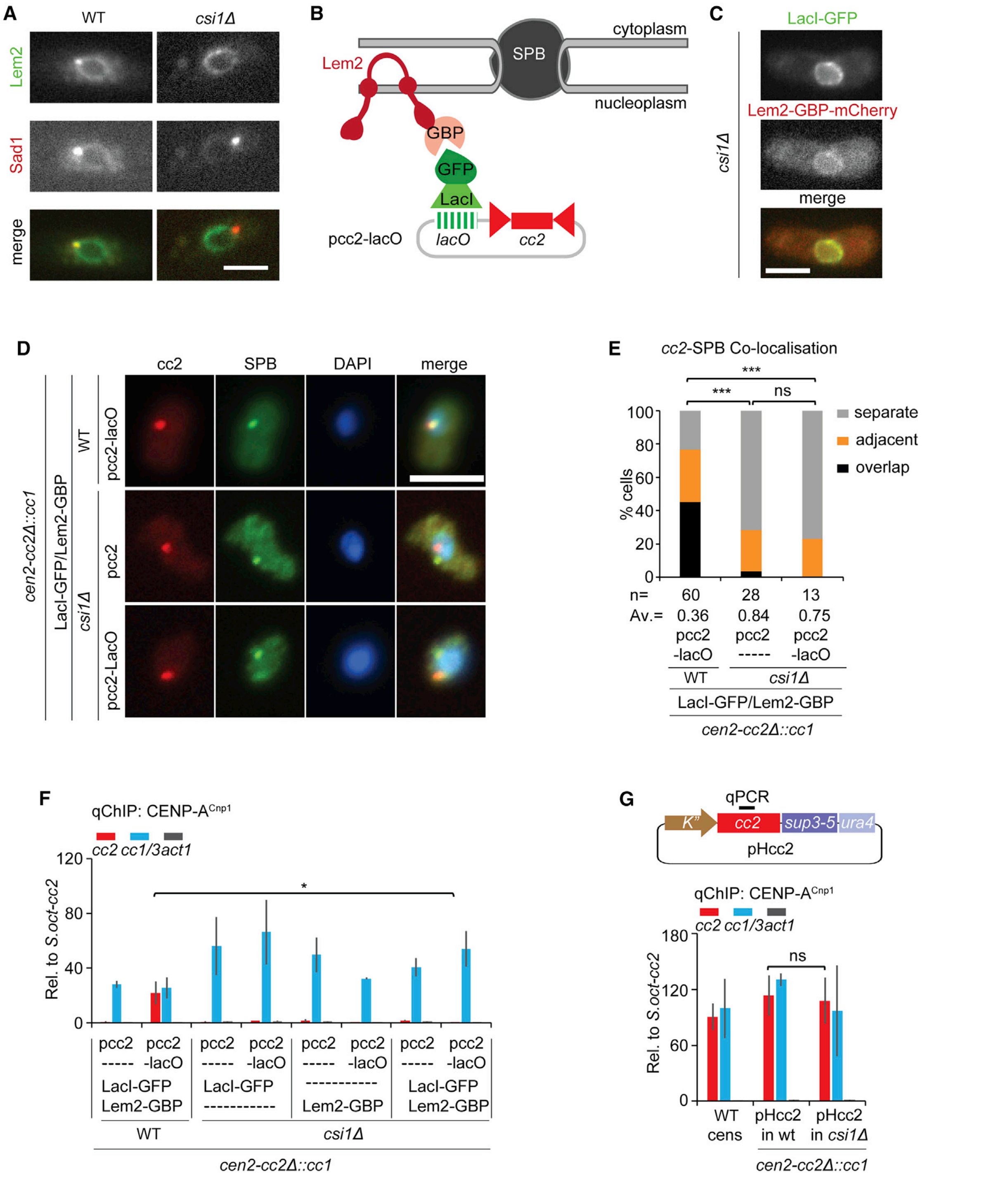

Figure 6. Loss of Csi1 prevents CENP-A^{Cnp1} chromatin establishment on Lem2-tethered pcc2-*lacO*

(A and C) Representative images of live WT and *csi1Δ* cells expressing Lem2-GFP and Sad1-dsRed (A) or LacI-GFP and Lem2-GBP-mCherry (C). Images were scaled as in Figure 2. Scale bars, 5 μm.

(B) Forced association of pcc2-lacO with Lem2-GBP-mCherry at NE in *csi1Δ* using same tethering system as in Figure 5. In *csi1Δ*, pcc2-lacO is expected to detach from the SPB due to loss of Lem2 from SPB.

(D) Representative images of *cc2* DNA FISH (red), SPB location (green, anti-Cdc11), and DNA staining (blue, DAPI) WT or *csi1Δ* strains expressing both LacI-GFP and Lem2-GBP-mCherry transformed with pcc2 or pcc2-lacO. Images were scaled as in Figure 1. Scale bars, 5 μm.

(legend continued on next page)

Although we do not detect CENP-A^{Cnp1} enrichment at sites between endogenous *cc1* or *neo1R* and *cc2* inserted at *lys1* or *itg8*, respectively, it is possible that CENP-A^{Cnp1} spreads in *cis* along intervening chromatin, but properties of these sequences provoke its loss. CENP-A^{Cnp1} spreading may be favored or hindered by innate sequence features such as their transcriptional activity. Low levels of transcription, open chromatin, or nucleosome turnover may promote CENP-A^{Cnp1} spreading. Moreover, the topology of the intervening chromatin may place a region physically closer or further away, depending on its level of compaction and/or looping in three-dimensional space.

Once assembled, CENP-A^{Cnp1} chromatin and thus kinetochores are self-propagating.[5,6,18] However, *de novo* establishment may be required if catastrophic events result in complete CENP-A^{Cnp1} loss. For example, a double-strand break and resection in the CENP-A^{Cnp1} chromatin domain of a centromere could result in disassembly of all CENP-A^{Cnp1} chromatin at that centromere. In such circumstances, continued association of the damaged centromere with the SPB via flanking heterochromatin could ensure the re-establishment of CENP-A^{Cnp1} chromatin and kinetochores following repair of central domain DNA.

Fission yeast neocentromeres arise most frequently in subtelomeric regions, and immature neocentromeres near rDNA can be stabilized by relocation to subtelomeric regions or upon acquisition of adjacent heterochromatin.[4,46] When overexpressed, CENP-A^{Cnp1} is incorporated at moderate levels over subtelomeric regions.[14] Therefore, subtelomeric regions represent favored, but secondary, sites for CENP-A^{Cnp1} and kinetochore assembly. H3K9me-dependent heterochromatin is normally assembled adjacent to telomeres.[47] During interphase, fission yeast telomeres are attached to the NE via INM proteins Bqt3 and Bqt4.[48] Although Hi-C analysis does not detect frequent contacts between telomere and centromere regions,[49] we suggest that as a consequence of their association with the nuclear periphery, subtelomeric regions are highly constrained in their nuclear explorations, essentially being confined to the NE's inner surface rather than having access to the entire nuclear volume. This constraint on spatial exploration would make telomeres more likely than arm sites to meet SPB-centromere clusters, thereby exposing them to the immediate nuclear compartment containing high levels of CENP-A^{Cnp1} and its assembly factors. Thus, nuclear-envelope association offers an attractive explanation for the subtelomeric location of most fission yeast neocentromeres. As neocentromeres arise rarely at telomeres, even in the absence of telomeric heterochromatin,[4] it is possible that telomere anchoring at the NE contributes to their role as secondary CENP-A^{Cnp1} assembly sites.

In fission yeast, centromeres cluster at the SPB throughout the cell cycle, except during mitosis, after which they return to the SPB in anaphase. CENP-A^{Cnp1} and several CENP-A^{Cnp1} assembly factors and chaperones, such as Scm3^{HJURP}, Mis16$^{RbAP46/48}$, Mis18, and Eic1/Mis19, are concentrated on centromeres around the SPB during interphase.[28–31] Mammalian centromeres are not localized close to centrosomes (SPB equivalent) during most of the cell cycle. However, after mitotic chromosome segregation, mammalian centromeres transiently cluster at spindle poles in late anaphase/telophase, subsequently dispersing during G1.[50] Centromere clustering is also pronounced in plants that exhibit an overt "Rabl" configuration, where centromeres and telomeres are clustered at opposite sides of interphase nuclei.[51] Intriguingly, the Mis18 CENP-A assembly complex is normally recruited to human centromeres in late anaphase/telophase prior to arrival of the HJURP CENP-A chaperone and new CENP-A incorporation in early G1.[18] Therefore, centromeres of complex eukaryotes are briefly clustered together at precisely the time when assembly factors are recruited to centromeres. This spatiotemporal coordination may maximize the local concentration of CENP-A and its assembly factors to ensure the efficient removal of H3 placeholder nucleosomes and the replenishment of CENP-A nucleosomes in centromeric chromatin.[52] However, we note that loss of CDK (cyclin-dependent kinase) regulation in mammalian cells allows premature CENP-A deposition in G2 cells.[53] Moreover, mammalian CENP-A can be loaded at centromeres exiting a manipulated mitosis (in the absence of microtubules and BubR1) without chromosome segregation/movement to the spindle poles.[54] The possible influence of centromere clustering on CENP-A assembly during normal mammalian cell cycles will require more direct investigation.

Once CENP-A^{Cnp1} chromatin and kinetochores are assembled at fission yeast centromeres, it is clear that heterochromatin-independent connections with SPBs are established. Centromeres remain clustered at SPBs in the absence of pericentromeric H3K9me-dependent heterochromatin, but SPB-centromere clustering is disrupted when essential kinetochore components such as Mis6 are defective.[37] Thus, once assembled, an intact interphase kinetochore structure, rather than pericentromeric heterochromatin, appears to provide the main physical link between functional centromeres and SPBs. Interestingly, cells defective in the essential kinetochore component Mis6 display both SPB-centromere declustering[37] and reduced CENP-A^{Cnp1} levels at centromeres,[55] suggesting that clustering might impact CENP-A^{Cnp1} maintenance at centromeres.

The tendency for heterochromatin to concentrate at SPBs may be mediated by interactions between heterochromatin proteins and SPB components. Indeed, proteomic analyses show that several SPB proteins are enriched with Swi6^{HP1} heterochromatin.[42] A plasmid-based minichromosome (ptetO) bearing completely synthetic TetR-Clr4-driven heterochromatin also colocalizes with SPBs, albeit less frequently than pHet bearing K-repeat-directed heterochromatin (Figures 1 and S5). Thus, in addition to heterochromatin itself, other unknown factors bound to K-repeat, but not synthetic heterochromatin, may contribute to robust SPB association. Although such factors may influence minichromosome-SPB association, it is clear that artificially tethering central domain DNA to SPBs bypasses the

(E) Percentage of interphase cells (n, number analyzed) displaying distinct degrees of *cc2* DNA colocalization with SPBs (Cdc11). Cells were classified into three groups as in Figure 1. AV, average distance. ns, no significance; ***p < 0.0001 (Mann-Whitney U test).

(F and G) qChIP analyses for CENP-A^{Cnp1} at *cc2*, *cc1/3*, and *act1* in indicated strains transform with pcc2 or pcc2-lacO (F) or pHcc2 (G). qChIP primer site on pHcc2-borne *cc2* is indicated as black bar above plasmid map (G). %IP levels in *S. pombe* were normalized to %IP of *S. octosporus* central core from spiked-in chromatin. Data are mean ± SD (n = 3). ns, no significance; *p < 0.05 (unpaired t test) (see also Figure S1).

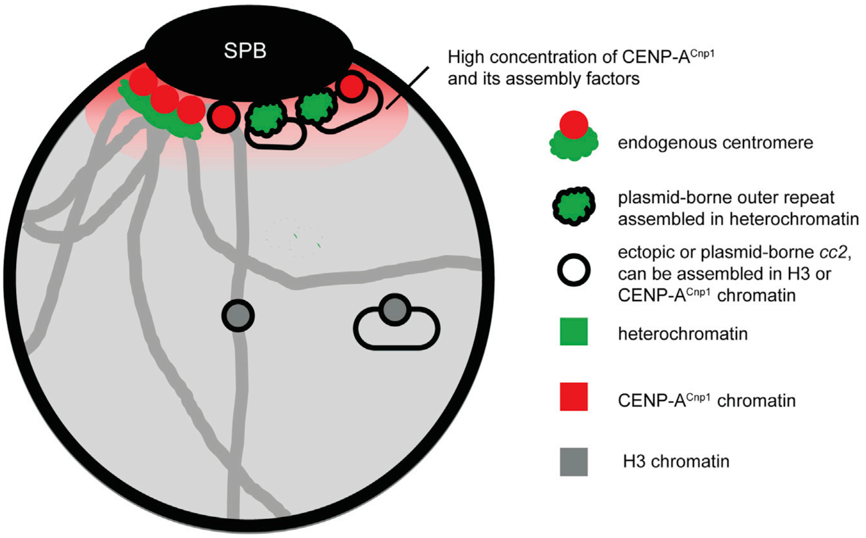

Figure 7. Model: Centromere identity is influenced by nuclear spatial organization

Due to clustering of endogenous centromeres ($CENP\text{-}A^{Cnp1}$-assembled central domains, red circles; heterochromatic outer repeats, green) at SPBs and incorporation of $CENP\text{-}A^{Cnp1}$ at centromeres in G2, the zone around SPBs forms a nuclear sub-compartment rich in $CENP\text{-}A^{Cnp1}$ and its assembly factors (red-shaded cloud). Ectopic central domain (outlined circles) inserted at centromere-proximal sites exposed the high-$CENP\text{-}A^{Cnp1}$ SPB/centromere sub-compartment, promoting *de novo* incorporation of $CENP\text{-}A^{Cnp1}$, unlike centromere-distal locations. Similarly, only minichromosomes bearing heterochromatin, which mediates localization close to the SPB, exposes the adjacent central domain to the high-$CENP\text{-}A^{Cnp1}$ SPB/centromere sub-compartment, resulting in $CENP\text{-}A^{Cnp1}$ incorporation. Heterochromatin, green; $CENP\text{-}A^{Cnp1}$, red; neutral H3 chromatin, gray (see also Figure S1).

need for heterochromatin for establishing high levels of $CENP\text{-}A^{Cnp1}$ chromatin in cells lacking Clr4-dependent heterochromatin (Figure S7).

Here, we have demonstrated that the specific location of centromere sequences within nuclei (i.e., their spatial context) exerts an epigenetic influence on the eventual CENP-A chromatin state attained by specific DNA sequences. Our analyses demonstrate that the SPB-centromere cluster forms a subcompartment within the nucleus that promotes CENP-A and kinetochore assembly on DNA sequences, presenting the required features to facilitate CENP-A chromatin assembly in place of canonical H3 chromatin. Thus, spatial positioning in the nucleus is a hitherto unrecognized epigenetic determinant of centromere identity

STAR★METHODS

Detailed methods are provided in the online version of this paper and include the following:

- KEY RESOURCES TABLE
- RESOURCE AVAILABILITY
  - ○ Lead contact
  - ○ Materials availability
  - ○ Data and code availability
- EXPERIMENTAL MODEL AND SUBJECT DETAILS
  - ○ Yeast strains
  - ○ Yeast growth medium and conditions
  - ○ Bacteria
- METHOD DETAILS
  - ○ Yeast genetic crosses
  - ○ Yeast colony PCR
  - ○ Yeast transformation
  - ○ Serial dilution spotting assays
  - ○ Centromere establishment assay on minichromosome pHcc2
  - ○ Quantitative chromatin immunoprecipitation (qChIP)
  - ○ Fluorescence microscopy
  - ○ Localization and fluorescence in situ hybridization (FISH)
  - ○ 3D distance measurements
- QUANTIFICATION AND STATISTICAL ANALYSIS

SUPPLEMENTAL INFORMATION

Supplemental information can be found online at https://doi.org/10.1016/j.cub.2022.06.048.

ACKNOWLEDGMENTS

We are grateful to the members of Allshire lab, especially Manu Shukla, Nitobe London, and Dominik Hoelper, for helpful suggestions and comments on the manuscript and Sharon White for organizational support. Takeshi Urano is thanked for the 5.1.1 (H3K9me) antibody, Ken Sawin for the Cdc11 antibody, Kevin Hardwick for the Spc7 antibody, Julie Cooper for the plasmid pF6a-GBP-mCherry-Hyg, and Nick Rhind for the *S. octosporus* strain. W.W. is supported by the Darwin Trust of Edinburgh. R.C.A. is a Wellcome Principal Research Fellow (095021 and 200885); the Wellcome Centre for Cell Biology is supported by core funding from Wellcome (203149). The Allshire lab dedicates this study to the memory of our dear colleague and friend Sasha Kagansky, whose research and insights were an inspiration for this project.

AUTHOR CONTRIBUTIONS

Conceptualization, W.W., A.L.P., and R.C.A.; methodology, W.W., T.M., D.A.K., and A.L.P.; investigation, W.W.; visualization, W.W.; writing – original draft, W.W.; writing – review & editing, W.W., A.L.P., and R.C.A.; funding acquisition, R.C.A.; supervision, A.L.P. and R.C.A.

DECLARATION OF INTERESTS

The authors declare no competing interests.

Received: December 28, 2021
Revised: April 24, 2022
Accepted: June 15, 2022
Published: July 12, 2022

CellPress
OPEN ACCESS

REFERENCES

1. Allshire, R.C., and Karpen, G.H. (2008). Epigenetic regulation of centromeric chromatin: old dogs, new tricks? Nat. Rev. Genet. *9*, 923–937.
2. Mellone, B.G., and Fachinetti, D. (2021). Diverse mechanisms of centromere specification. Curr. Biol. *31*, R1491–R1504.
3. DeBose-Scarlett, E.M., and Sullivan, B.A. (2021). Genomic and epigenetic foundations of neocentromere formation. Annu. Rev. Genet. *55*, 331–348.
4. Ishii, K., Ogiyama, Y., Chikashige, Y., Soejima, S., Masuda, F., Kakuma, T., Hiraoka, Y., and Takahashi, K. (2008). Heterochromatin integrity affects chromosome reorganization after centromere dysfunction. Science *321*, 1088–1091.
5. Mitra, S., Srinivasan, B., and Jansen, L.E.T. (2020). Stable inheritance of CENP-A chromatin: inner strength versus dynamic control. J. Cell Biol. *219*, e202005099. https://doi.org/10.1083/jcb.202005099.
6. Westhorpe, F.G., and Straight, A.F. (2014). The centromere: epigenetic control of chromosome segregation during mitosis. Cold Spring Harb. Perspect. Biol. *7*, a015818. https://doi.org/10.1101/cshperspect.a015818.
7. Allshire, R.C., and Ekwall, K. (2015). Epigenetic regulation of chromatin states in *Schizosaccharomyces pombe*. Cold Spring Harb. Perspect. Biol. *7*, a018770. https://doi.org/10.1101/cshperspect.a018770.
8. Martienssen, R., and Moazed, D. (2015). RNAi and heterochromatin assembly. Cold Spring Harb. Perspect. Biol. *7*, a019323. https://doi.org/10.1101/cshperspect.a019323.
9. Clarke, L., Baum, M., Marschall, L.G., Ngan, V.K., and Steiner, N.C. (1993). Structure and function of *Schizosaccharomyces pombe* centromeres. Cold Spring Harb. Symp. Quant. Biol. *58*, 687–695.
10. Takahashi, K., Murakami, S., Chikashige, Y., Funabiki, H., Niwa, O., and Yanagida, M. (1992). A low copy number central sequence with strict symmetry and unusual chromatin structure in fission yeast centromere. Mol. Biol. Cell *3*, 819–835.
11. Noma, K., Cam, H.P., Maraia, R.J., and Grewal, S.I. (2006). A role for TFIIIC transcription factor complex in genome organization. Cell *125*, 859–872.
12. Scott, K.C., Merrett, S.L., and Willard, H.F. (2006). A heterochromatin barrier partitions the fission yeast centromere into discrete chromatin domains. Curr. Biol. *16*, 119–129.
13. Tong, P., Pidoux, A.L., Toda, N.R.T., Ard, R., Berger, H., Shukla, M., et al. (2019). Interspecies conservation of organisation and function between nonhomologous regional centromeres. Nat. Commun. *10*, 2343. https://doi.org/10.1038/s41467-019-09824-4.
14. Castillo, A.G., Pidoux, A.L., Catania, S., Durand-Dubief, M., Choi, E.S., Hamilton, G., et al. (2013). Telomeric repeats facilitate CENP-A(Cnp1) incorporation via telomere binding proteins. PLoS One *8*, e69673. https://doi.org/10.1371/journal.pone.0069673.
15. Catania, S., Pidoux, A.L., and Allshire, R.C. (2015). Sequence features and transcriptional stalling within centromere DNA promote establishment of CENP-A chromatin. PLoS Genet *11*, e1004986. https://doi.org/10.1371/journal.pgen.1004986.
16. Shukla, M., Tong, P., White, S.A., Singh, P.P., Reid, A.M., Catania, S., et al. (2018). Centromere DNA destabilizes H3 nucleosomes to promote CENP-A deposition during the cell cycle. Curr. Biol. *28*, 3924–3936.e4. https://doi.org/10.1016/j.cub.2018.10.049.
17. Singh, P.P., Shukla, M., White, S.A., Lafos, M., Tong, P., Auchynnikava, T., Spanos, C., Rappsilber, J., Pidoux, A.L., and Allshire, R.C. (2020). Hap2-Ino80-facilitated transcription promotes *de novo* establishment of CENP-A chromatin. Genes Dev *34*, 226–238.
18. Zasadzińska, E., and Foltz, D.R. (2017). Orchestrating the specific assembly of centromeric nucleosomes. Prog. Mol. Subcell. Biol. *56*, 165–192.
19. Funabiki, H., Hagan, I., Uzawa, S., and Yanagida, M. (1993). Cell cycle-dependent specific positioning and clustering of centromeres and telomeres in fission yeast. J. Cell Biol. *121*, 961–976.
20. Muller, H., Gil, J., Jr., and Drinnenberg, I.A. (2019). The impact of centromeres on spatial genome architecture. Trends Genet *35*, 565–578.
21. Winey, M., and O'Toole, E.T. (2001). The spindle cycle in budding yeast. Nat. Cell Biol. *3*, E23–E27.
22. Guacci, V., Hogan, E., and Koshland, D. (1997). Centromere position in budding yeast: evidence for anaphase A. Mol. Biol. Cell *8*, 957–972.
23. Jaspersen, S.L. (2021). Anatomy of the fungal microtubule organizing center, the spindle pole body. Curr. Opin. Struct. Biol. *66*, 22–31.
24. Ebrahimi, H., Masuda, H., Jain, D., and Cooper, J.P. (2018). Distinct 'safe zones' at the nuclear envelope ensure robust replication of heterochromatic chromosome regions. eLife *7*, e32911. https://doi.org/10.7554/eLife.32911.
25. Fernández-Álvarez, A., Bez, C., O'Toole, E.T., Morphew, M., and Cooper, J.P. (2016). Mitotic nuclear envelope breakdown and spindle nucleation are controlled by interphase contacts between centromeres and the nuclear envelope. Dev. Cell *39*, 544–559.
26. Barrales, R.R., Forn, M., Georgescu, P.R., Sarkadi, Z., and Braun, S. (2016). Control of heterochromatin localization and silencing by the nuclear membrane protein Lem2. Genes Dev *30*, 133–148.
27. Hou, H., Zhou, Z., Wang, Y., Wang, J., Kallgren, S.P., Kurchuk, T., Miller, E.A., Chang, F., and Jia, S. (2012). Csi1 links centromeres to the nuclear envelope for centromere clustering. J. Cell Biol. *199*, 735–744.
28. Hayashi, T., Fujita, Y., Iwasaki, O., Adachi, Y., Takahashi, K., and Yanagida, M. (2004). Mis16 and Mis18 are required for CENP-A loading and histone deacetylation at centromeres. Cell *118*, 715–729.
29. Pidoux, A.L., Choi, E.S., Abbott, J.K., Liu, X., Kagansky, A., Castillo, A.G., Hamilton, G.L., Richardson, W., Rappsilber, J., He, X., and Allshire, R.C. (2009). Fission yeast Scm3: a CENP-A receptor required for integrity of subkinetochore chromatin. Mol. Cell *33*, 299–311.
30. Subramanian, L., Toda, N.R., Rappsilber, J., and Allshire, R.C. (2014). Eic1 links Mis18 with the CCAN/Mis6/Ctf19 complex to promote CENP-A assembly. Open Biol *4*, 140043. https://doi.org/10.1098/rsob.140043.
31. Williams, J.S., Hayashi, T., Yanagida, M., and Russell, P. (2009). Fission yeast Scm3 mediates stable assembly of Cnp1/CENP-A into centromeric chromatin. Mol. Cell *33*, 287–298.
32. Folco, H.D., Pidoux, A.L., Urano, T., and Allshire, R.C. (2008). Heterochromatin and RNAi are required to establish CENP-A chromatin at centromeres. Science *319*, 94–97.
33. Steiner, N.C., and Clarke, L. (1994). A novel epigenetic effect can alter centromere function in fission yeast. Cell *79*, 865–874.
34. Kagansky, A., Folco, H.D., Almeida, R., Pidoux, A.L., Boukaba, A., Simmer, F., Urano, T., Hamilton, G.L., and Allshire, R.C. (2009). Synthetic heterochromatin bypasses RNAi and centromeric repeats to establish functional centromeres. Science *324*, 1716–1719.
35. Alfredsson-Timmins, J., Henningson, F., and Bjerling, P. (2007). The Clr4 methyltransferase determines the subnuclear localization of the mating-type region in fission yeast. J. Cell Sci. *120*, 1935–1943.
36. Pichugina, T., Sugawara, T., Kaykov, A., Schierding, W., Masuda, K., Uewaki, J., et al. (2016). A diffusion model for the coordination of DNA replication in *Schizosaccharomyces pombe*. Sci. Rep. *6*, 18757. https://doi.org/10.1038/srep18757.
37. Saitoh, S., Takahashi, K., and Yanagida, M. (1997). Mis6, a fission yeast inner centromere protein, acts during G1/S and forms specialized chromatin required for equal segregation. Cell *90*, 131–143.
38. Bernard, P., Maure, J.F., Partridge, J.F., Genier, S., Javerzat, J.P., and Allshire, R.C. (2001). Requirement of heterochromatin for cohesion at centromeres. Science *294*, 2539–2542.
39. Nonaka, N., Kitajima, T., Yokobayashi, S., Xiao, G., Yamamoto, M., Grewal, S.I., and Watanabe, Y. (2002). Recruitment of cohesin to heterochromatic regions by Swi6/HP1 in fission yeast. Nat. Cell Biol. *4*, 89–93.
40. Audergon, P.N., Catania, S., Kagansky, A., Tong, P., Shukla, M., Pidoux, A.L., and Allshire, R.C. (2015). Epigenetics. Restricted epigenetic inheritance of H3K9 methylation. Science *348*, 132–135.
41. Ding, D.Q., and Hiraoka, Y. (2017). Visualization of a specific genome locus by the *lacO*/LacI-GFP system. Cold Spring Harb. Protoc. *2017*. pdb.prot091934. https://doi.org/10.1101/pdb.prot091934.

42. Iglesias, N., Paulo, J.A., Tatarakis, A., Wang, X., Edwards, A.L., Bhanu, N.V., et al. (2020). Native chromatin proteomics reveals a role for specific nucleoporins in heterochromatin organization and maintenance. Mol. Cell *77*, 51–66.e8.

43. Bestul, A.J., Yu, Z., Unruh, J.R., and Jaspersen, S.L. (2017). Molecular model of fission yeast centrosome assembly determined by superresolution imaging. J. Cell Biol. *216*, 2409–2424.

44. Choi, E.S., Strålfors, A., Catania, S., Castillo, A.G., Svensson, J.P., Pidoux, A.L., et al. (2012). Factors that promote H3 chromatin integrity during transcription prevent promiscuous deposition of CENP-A(Cnp1) in fission yeast. PLoS Genet. *8*, e1002985. https://doi.org/10.1371/journal.pgen.1002985.

45. Klutstein, M., Fennell, A., Fernández-Álvarez, A., and Cooper, J.P. (2015). The telomere bouquet regulates meiotic centromere assembly. Nat. Cell Biol. *17*, 458–469.

46. Ogiyama, Y., Ohno, Y., Kubota, Y., and Ishii, K. (2013). Epigenetically induced paucity of histone H2A.Z stabilizes fission-yeast ectopic centromeres. Nat. Struct. Mol. Biol. *20*, 1397–1406.

47. Kanoh, J., Sadaie, M., Urano, T., and Ishikawa, F. (2005). Telomere binding protein Taz1 establishes Swi6 heterochromatin independently of RNAi at telomeres. Curr. Biol. *15*, 1808–1819.

48. Chikashige, Y., Yamane, M., Okamasa, K., Tsutsumi, C., Kojidani, T., Sato, M., Haraguchi, T., and Hiraoka, Y. (2009). Membrane proteins Bqt3 and -4 anchor telomeres to the nuclear envelope to ensure chromosomal bouquet formation. J. Cell Biol. *187*, 413–427.

49. Mizuguchi, T., Fudenberg, G., Mehta, S., Belton, J.M., Taneja, N., Folco, H.D., FitzGerald, P., Dekker, J., Mirny, L., Barrowman, J., and Grewal, S. (2014). Cohesin-dependent globules and heterochromatin shape 3D genome architecture in *S. pombe*. Nature *516*, 432–435.

50. Gerlich, D., Beaudouin, J., Kalbfuss, B., Daigle, N., Eils, R., and Ellenberg, J. (2003). Global chromosome positions are transmitted through mitosis in mammalian cells. Cell *112*, 751–764.

51. Oko, Y., Ito, N., and Sakamoto, T. (2020). The mechanisms and significance of the positional control of centromeres and telomeres in plants. J. Plant Res. *133*, 471–478.

52. Dunleavy, E.M., Almouzni, G., and Karpen, G.H. (2011). H3.3 is deposited at centromeres in S phase as a placeholder for newly assembled CENP-A in G1 phase. Nucleus *2*, 146–157.

53. Stankovic, A., Guo, L.Y., Mata, J.F., Bodor, D.L., Cao, X.J., Bailey, A.O., Shabanowitz, J., Hunt, D.F., Garcia, B.A., Black, B.E., and Jansen, L. (2017). A dual inhibitory mechanism sufficient to maintain cell-cycle-restricted CENP-A assembly. Mol. Cell *65*, 231–246.

54. Jansen, L.E., Black, B.E., Foltz, D.R., and Cleveland, D.W. (2007). Propagation of centromeric chromatin requires exit from mitosis. J. Cell Biol. *176*, 795–805.

55. Takahashi, K., Chen, E.S., and Yanagida, M. (2000). Requirement of Mis6 centromere connector for localizing a CENP-A-like protein in fission yeast. Science *288*, 2215–2219.

56. Rhind, N., Chen, Z., Yassour, M., Thompson, D.A., Haas, B.J., Habib, N., Wapinski, I., Roy, S., Lin, M.F., Heiman, D.I., et al. (2011). Comparative functional genomics of the fission yeasts. Science *332*, 930–936.

57. Torres-Garcia, S., Di Pompeo, L., Eivers, L., Gaborieau, B., White, S.A., Pidoux, A.L., et al. (2020). SpEDIT: a fast and efficient CRISPR/Cas9 method for fission yeast. Wellcome Open Res. *5*, 274. https://doi.org/10.12688/wellcomeopenres.16405.1.

58. Moreno, S., Klar, A., and Nurse, P. (1991). Molecular genetic analysis of fission yeast *Schizosaccharomyces pombe*. Methods Enzymol. *194*, 795–823.

59. Bähler, J., Wu, J.Q., Longtine, M.S., Shah, N.G., McKenzie, A., 3rd, Steever, A.B., Wach, A., Philippsen, P., and Pringle, J.R. (1998). Heterologous modules for efficient and versatile PCR-based gene targeting in *Schizosaccharomyces pombe*. Yeast *14*, 943–951.

60. Castillo, A.G., Mellone, B.G., Partridge, J.F., Richardson, W., Hamilton, G.L., Allshire, R.C., et al. (2007). Plasticity of fission yeast CENP-A chromatin driven by relative levels of histone H3 and H4. PLoS Genet. *3*, e121. https://doi.org/10.1371/journal.pgen.0030121.

CellPress
OPEN ACCESS

STAR★METHODS

KEY RESOURCES TABLE

REAGENT or RESOURCE	SOURCE	IDENTIFIER
Antibodies		
Mouse monoclonal anti-H3K9me2	Gift from Takeshi Urano, mAb5.1.1	N/A
Sheep polyclonal anti-CENP-A^{Cnp1}	In-house preparation[15]	N/A
Sheep polyclonal anti-CENP-C^{Cnp3}	In-house preparation[15]	N/A
Sheep polyclonal anti-CENP-K^{Sim4}	In-house preparation[15]	N/A
Sheep polyclonal anti-KNL1^{Spc7}	Gift from Kevin Hardwick	N/A
Sheep anti-Cdc11	Gift from Ken Sawin	N/A
Donkey anti-Sheep IgG (H+L) Cross-Adsorbed Secondary Antibody, Alexa Fluor 488	Thermo Fisher Scientific	Cat#A-11015; RRID: AB_2534082
Sheep polyclonal Anti-Digoxigenin-Rhodamine, Fab fragments	Roche	Cat#11207750910; RRID: AB_514501
Bacterial and virus strains		
NEB 5-alpha Competent E. coli (High Efficiency)	New England Biolabs	Cat#C2987H
Chemicals, peptides, and recombinant proteins		
Nourseothricin (cloNAT)	Werner BioAgents	CAS#96736-11-7
Hygromycin B (Hyg)	Duchefa Biochemie	CAS#31282-04-9
Geneticin Selective Antibiotic (G418 Sulfate)	Gibco Life Technologies	CAS#10131027
Carbenicillin Disodium Salt	Invitrogen	CAS#10177012
Formaldehyde, 37%	MERCK	CAS#F8775
Glycine	MERCK	G8790
Glutaraldehyde solution, 50%	MERCK	CAS#111-30-8
Zymolyase-100T	MP Biomedicals	Cat#08320932
Lallzyme	Litmus Wines	Lallzyme-MMX
Protein G Dynabeads (Life Technologies)	Thermo Fisher Scientific	Cat#10009D
Protein G-Agarose	Roche	Cat#11243233001
Critical commercial assays		
QIAquick PCR Purification Kit	QIAGEN	Cat#28104
Monarch Plasmid Miniprep Kit	New England Biolabs	Cat#T1010L
FastStart Taq DNA Polymerase	Roche	Cat#12032953001
Light Cycler 480 SybrGreen Master Mix	Roche	Cat#04887352001
DIG-Nick Translation Mix	Roche	Cat#11745816910
NEB Golden Gate Assembly Kit (BsaI-HF v2)	New England Biolabs	Cat# E1601S
Experimental models: Organisms/strains		
S. pombe strains, see Table S1	This study	N/A
S. octosporus	From Nick Rhind[56]	yFS286
Oligonucleotides		
Primers, see Table S3	This study	N/A
Recombinant DNA		
Plasmid pLSB-Kan, see Table S2	[57]	Addgene#166700; RRID: Addgene_166700
Plasmid clr4-pLSB-Kan, see Table S2	This study	N/A
Plasmid pMC52, see Table S2	This study	N/A
Plasmid pFA6a-GBP-mCherry-hygMX6, see Table S2	Gift from Julia Promisel Cooper[25]	N/A

(*Continued on next page*)

Continued		
REAGENT or RESOURCE	SOURCE	IDENTIFIER
Plasmid pFA6a-GFP-NatMX6, see Table S2	This study	N/A
Plasmid pMC2 (pcc2), see Table S2	This study	N/A
Plasmid pMC12 (pcc2-lacO), see Table S2	This study	N/A
Plasmid pHcc2, see Table S2	This study	N/A
Plasmid pMC183 (pHET), see Table S2	This study	N/A
Plasmid pMC1, see Table S2	This study	N/A
Software and algorithms		
Roche LightCycler software version 1.5.1.62	Roche	N/A
Nikon NIS Elements software version 5.21.03	Nikon	RRID: SCR_014329
Fiji	ImageJ, http://fiji.sc	RRID: SCR_002285
Fiji-based bespoke in-house 3D analysis code	This study; https://doi.org/10.5281/zenodo.5657360	N/A
SnapGene 5.2.5	GSL Biotech LLC	RRID: SCR_015052
Prism Version 9.1.0	GraphPad	RRID: SCR_002798
pombase	https://www.pombase.org/	RRID: SCR_006586
Other		
Glusulase	NEN	NEE-154
*Kpn*I-HF	New England Biolabs	Cat#R3142S
*Xho*I	New England Biolabs	Cat# R0146S
*Sac*I-HF	New England Biolabs	Cat# R3156S
*Msc*I	New England Biolabs	Cat# R0534S
T4 DNA Ligase	New England Biolabs	Cat# M0202S
PMSF	MERCK	CAS#329-98-6
Yeast Protease Inhibitor Cocktail	MERCK	P8215
IGEPAL CA-630 NP40	MERCK	Cat# 56741
Chelex 100 Chelating Resin	Bio-Rad	Cat#1421253
VECTASHIELD Antifade Mounting Medium	Vector Laboratories	Cat# H-1000-10
L-Lysine hydrochloride	MERCK	Cat# 657-27-2
Bovine Serum Albumin (BSA)	MERCK	Cat# A0281
RNase A	Qiagen	Cat#19101
Dextran sulfate sodium salt	MERCK	D8906
deionized formamide	MERCK	S4117
Denhardt's Solution 50x	MERCK	D2532
Gelatin from cold water fish skin	MERCK	Cat#G7765
Ambion DNase I (RNase-free)	Thermo Fisher Scientific	Cat#AM2222

RESOURCE AVAILABILITY

Lead contact

Requests concerning resources or material should be directed to and will be fulfilled by the lead contact Robin Allshire (robin.allshire@ed.ac.uk).

Materials availability

All plasmids and *Schizosaccharomyces pombe* (fission yeast) strains generated or used for this study are available form the lead contact without restriction.

Data and code availability

- All original microscopy images, qChIP and 3D distance measurements data reported in this paper will be shared by the lead contact upon request.

- All original code has been deposited at GitHub (https://zenodo.org/record/5657360#.Yn7YZBPMLUJ) and is publicly available as of the date of publications. DOIs is listed in the key resources table.
- Any additional information required to reanalyze the data reported in this paper is available from the Lead Contact upon request.

EXPERIMENTAL MODEL AND SUBJECT DETAILS

Yeast strains

Yeast strains used in this study and their genotypes are listed in Table S1.

Standard genetic and molecular methods were used as described.[58] All ectopic *cc2* insertions were made in *cc2*Δ::*cc1* strains[15] by integrating linear *cen2* central domain constructs (~880 bp *imr2L*, -6.8 kb *cc2* and ~920 bp *imr2R*, abbreviated as *cc2*) by homologous recombination (HR). pMC52 (Table S2), bearing 8.6 kb of *cc2* and kanMX6 selection cassette, was used as a starting plasmid for linear *cc2* constructs. Two flanking DNA fragments of the desired target locus for *cc2* insertions were amplified using primers listed in Table S3 by PCR. Restriction enzyme *Kpn*I/*Xho*I-digested first fragment was cloned into *Kpn*I/*Xho*I-digested pMC52, which were then digested by *Sac*I/*Msc*I and ligated with *Sac*I/*Msc*I-digested second PCR fragment by T4 DNA ligase (M0202S; NEB). Linear *cc2* constructs were obtained by *Sac*I/*Kpn*I digestion of the resulting plasmids and transformed into desired strain for *cc2* insertion.

For the construction of Lem2/Alp4/Alp6-GBP-mCherry and Lem2-GFP, the GBP-mCherry-hygMX6 and GFP-natMX6 cassette in plasmid pFA6a-GBP-mCherry-hygMX6[25] and pFA6a-GFP-NatMX6 were amplified by PCR and integrated into genome by HR.[59]

*clr4*Δ mutant was created by CRISPR/Cas9 method as described previously.[57] Briefly, *clr4* gene-specific sgRNA was cloned into Cas9 containing pLSB-KAN plasmid by Golden Gate Assembly kit (E1601S, NEB). The resulting plasmid *clr4*-pLSB-KAN and *clr4* HR template obtained by annealing primer pair WW748/WW749 (Table S3) were co-transformed into *S. pombe* by sorbitol-electroporation method.

Transformants were grown on appropriate selection plates and screened for correct integration or *clr4Δ* mutant by yeast colony PCR using primers listed in Table S3. All plasmids and primers used in this study are listed in Tables S2 and S3 respectively.

Yeast growth medium and conditions

All strains were grown at 32°C in YES (Yeast Extract with Supplements) rich medium or PMG (Pombe Minimal Glutamate) minimal medium, as appropriate. Selection for dominant markers was performed on YES medium supplemented with 100 μg/ml clonNAT (96736-11-7, Werner BioAgents), 100 μg/ml G418 (10131027, Gibco), or 123 μg/ml HygMX6 (31282-04-9, Duchefa Biochemie). *clr4Δ* transformants were selected on YES supplemented with G418 plate and re-streaked to non-selective YES medium to allow loss of plasmid clr4-pLSB-KAN. Transformants with *cc2* insertions were selected on YES supplemented G418. Plasmids pcc2 (pMC2; carrying 8.6 kb of *cc2*) and pcc2-LacO (pMC12; carrying 8.6 kb of *cc2* and 2.8 kb of *lacO*) were selected on YES containing 100 μg/ml G418 in wt strains or on PMG-uracil in *csi1*Δ (*csi1*Δ::*ura4*) strain. Strains carrying plasmid pHet (pMC183; carrying 2 kb of *K*'' repeats), ptetO (pMC171; bearing 4 *tet* operators embedded in 2 kb of randomized AT-rich sequence) were selected on YES supplemented with clonNAT or whereas pHcc2 (H denotes 5.6 kb of *K*'' repeats, *cc2* denotes 8.6 kb of *cc2*) were selected on PMG-adenine-uracil medium, respectively.

Bacteria

DH5α E. coli strains (C2987H, NEB) were grown in LB medium at 37°C. E. coli competent cells carrying plasmids were selected on LB agar plates supplemented with 100 μg/ml of ampicillin or LB liquid supplemented with 50 μg/ml Carbenicillin (10177012; Invitrogen).

METHOD DETAILS

Yeast genetic crosses

To obtain desired genotypes, two strains with opposite mating type (h+/h-) were mixed and grown on the nitrogen starved ME plate for sporulation at 32°C for 2 days. Asci was digested in glusulase (NEE-154, NEN) to release spores that were then plated on appropriate selective medium and grown at 32°C.

Yeast colony PCR

Yeast strains were suspended in SPZ buffer (1.2 M sorbitol, 100 mM sodium phosphate and 2.5 mg/ml Zymolyase-100T (08320932, MP Biomedicals)) and incubated at 37°C for 30 min. The resulted mixtures were used as PCR template for strain genotyping by Roche FastStart Taq polymerase PCR kit (12032953001, Roche) supplemented with primers.

Yeast transformation

Yeast cells were transformed using the sorbitol-electroporation method. Log phase cultures were harvested and resuspended in pre-transformation buffer (25 mM DTT, 0.6 M sorbitol and 20 mM HEPES, pH7.6) and incubated at 32°C with 180 rpm shaking for 10 min. Cells were washed three times in ice-cold 1.2 M sorbitol, mixed in an ice-cold cuvette with 200 ng of plasmid DNA or purified DNA fragments obtained by QIAquick PCR Purification Kit (28104, QIAGEN) and then pulsed by an electroporator (Bio-Rad Gene Pulser II)

at a setting of 2.25kV, 200Ω and 25μF. Cells were either directly plated on medium with prototrophic selection directly or grown overnight in non-selective liquid before selection for antibiotic resistance (G418/cloNAT/HygMX6). Single colonies were isolated from selective medium.

Serial dilution spotting assays

Equal amounts of starting cells for each strain were serially diluted 5-fold and then spotted onto PMG plate complemented with 2.5 ug/ml vital dye Phloxine B. Colonies with a higher proportion of dead cells stain darker pink. Cells were grown at 25 °C or 32 °C for 3-5 days and then photographed.

Centromere establishment assay on minichromosome pHcc2

Fresh transformant colonies carrying circular plasmid-based minichromosome pHcc2 were replica-plated from PMG -adenine -uracil to PMG low-adenine plates (10 μg/ml adenine) and incubated at 32°C for 2 days to determine initial frequency of establishment of functional centromeres. Plasmid pHcc2 contains the *sup3e* tRNA selection marker that suppresses the *ade6-704* mutation within strains, thus colony color on these PMG low-adenine plates will indicate minichromosome loss (red colonies) or retention (white/pale pink colonies). In the absence of centromere establishment, minichromosomes behave as episomes that are rapidly lost. Minichromosomes that established functional centromere (need both heterochromatin and CENP-A^{Cnp1} chromatin) segregate efficiently during mitosis. Minichromosomes which occasionally integrate at genome will give a false-positive white phenotype. To assess the frequency of such integration events and to confirm establishment of centromere segregation function, colonies providing a the white/pale-pink phenotype upon replica plating were re-streaked to single colonies on PMG-low-adenine plates. Red/white sectored colonies are indicative of centromere function with low levels of minichromosome loss, whereas pure white colonies are indicative of integration into endogenous chromosomes. Therefore, the number of sectored colonies divided by the number of total colonies (minus pure white colonies) was used to calculate the centromere establishment frequency (%) on minichromosome pHcc2.

Quantitative chromatin immunoprecipitation (qChIP)

For ChIP of cells containing plasmid minichromosomes, three independent transformant colonies were randomly picked from PMG-ade-ura or YES+antibiotic plates and grown to 50-100 ml cultures in appropriate selective media (the centromere-establishment status of colonies (if relevant) was not determined prior to picking). Log phase cultures were fixed in 1% formaldehyde (F8775, MERCK) for 15 min followed by quenching in 125 mM Glycine (G8790, MERCK) at room temperature. ChIP was performed as previously described.[60] $2.5x10^8$ cells were used for each ChIP. Briefly, cells were lysed by bead beating (Biospec) in 350 μl Lysis Buffer (50 mM Hepes-KOH pH 7.5, 140 mM NaCl, 1 mM EDTA, 1% (v/v) Triton X-100 and 0.1% (w/v) sodium deoxycholate) supplemented with 3.5 μl of 100 mM PMSF (329-98-6, MERCK) and 3.5 μl of 100 mM yeast protease inhibitor (P8215, MERCK). Where indicated, $\sim 5x10^7$ fixed, lysed *S. octosporus* cells[56] were added to each initial crude cell lysates as a spike-in control. Crude cell lysates were sonicated using a Bioruptor (Diagenode) at 4°C on high voltage for 20 min (20 cycles of 30 s ON/OFF), followed by centrifugation at 13000 rpm for 10 min to pellet cell debris. The resulting supernatant was used for following steps.

For H3K9me2 ChIP, 10 μl lysate was retained as crude 'input' sample, whereas 300 μl of the remaining lysates were incubated overnight with 20 μl of washed protein G Dynabeads (10009D, Thermo Fisher Scientific) and 1 μl of mouse anti-H3K9me2 (mAb5.1.1, gift from Takeshi Urano).

For CENP-A^{Cnp1}/CENP-C^{Cnp3}/Knl^{Spc7} ChIP, lysates were precleared for 1 h with 25 μl of washed protein-G agarose beads (11243233001, Roche) and 10 μl of precleared lysate was retained as crude 'input' sample. 300 μl of the remaining pre-cleared lysates were incubated overnight with appropriate amount of antibody (10 μl of sheep CENP-A^{Cnp1}, CENP-C^{Cnp3}, CENP-K^{Sim4} serum[15] (in-house preparation), 3 μl of affinity-purified sheep anti-Spc7 (a gift from Kevin Hardwick) and 25 μl of protein-G agarose beads.

After immunoprecipitation, the crude "IP" samples on beads were washed in Lysis Buffer, Lysis Buffer supplemented with 500 mM NaCl, Wash Buffer (10 mM Tris-HCl pH 8, 250 mM LiCl, 0.5% IGEPAL NP40 (56741, MERCK) 0.5% (w/v) sodium deoxycholate and 1 mM EDTA) and TE Buffer (10mM Tris-HCl pH 8, 1 mM EDTA). DNA was recovered from input and IP samples using Chelex resin (1421253, BioRad). Quantitative PCR reactions (qPCR) were performed using a LightCycler 480 SybrGreen Master Mix (04887352001, Roche) and analyzed using Roche LightCycler software (version 1.5.1.62). Primers used for qPCR are listed in Table S3. ChIP enrichments on regions of interest were calculated as the ratio of "IP" sample to the corresponding "input" sample using the ΔCT method and represented as %IP. Where indicated, for spike-in qChIPs, %IP levels in *S. pombe* were normalized to % IP from spiked-in *S. octosporus* chromatin (specified in the figure legends).

Fluorescence microscopy

Live fission yeast cells were mounted on a 2% agarose pad formed on 1 mm SuperFrost slides (Thermo Scientific) whereas fixed cells (immunofluorescence and DNA FISH) were mounted in VECTASHIELD Mounting Medium (H-1000-10, Vector Laboratories) on 1 mm Polysine slides (Thermo Scientific). Microscopy was performed with Nikon Ti2 inverted microscope equipped with a ×100 1.49 NA CFI Plan Apochromat TIRF objective, Lumencor Spectra X light source (Lumencor, Beaverton, OR USA) and a Photometrics Prime 95B camera (Teledyne Photometrics, Birmingham, UK), all controlled by Nikon NIS Elements software version 5.21.03 (RRID: SCR_014329). Filter sets from Semrock (Semrock, Rochester, New York, USA) were used to image Lem2-GFP, LacI-GFP, Alexa Fluor 488 (A-11015, Invitrogen) at excitation 488 nm, emission 535 nm, Sad1-dsRed, Rhodamine at excitation 554 nm, emission 590 nm, Lem2/Alp4/Alp6-mCherry at excitation 578 nm, emission 630 nm and DAPI, excitation 378 nm, emission 460 nm. A Mad

CellPress
OPEN ACCESS

City nano drive (Mad City Labs, Madison, WI, USA) was used to produce whole cell 3 dimensional (3D) images with a step size of 0.3 μm. All images were processed by Fiji software (RRID: SCR_002285). Live cell images were scaled relative to the maximum intensity in the set of images to allow comparison between images, but fixed cell images were scaled relative to the maximum value of histogram (specified in figure legends).

Localization and fluorescence in situ hybridization (FISH)

For Immunofluorescence/DNA FISH, cells were initially subjected to a similar Immunofluorescence protocol as described previously with some modifications[60] and subsequent FISH process. Briefly, log phase yeast cultures were fixed with 3.7% formaldehyde for 7 min at room temperature, washed by PEM buffer (100 mM PIPES pH 7, 1 mM EDTA, 1 mM $MgCl_2$) and PEMS buffer (100 mM PIPES pH 7, 1 mM EDTA, 1 mM $MgCl_2$, 1.2 M Sorbitol), followed by cell-wall digestion in PEMS buffer supplemented with 1 mg/ml Zymolyase-100T and 1 mg/ml Lallzyme (Lallzyme-MMX, Litmus Wines) at 37°C for 90 min. After permeabilization in PEMS containing 1% Triton X-100 for 5 min at room temperature, cells were washed, blocked in PEMBAL (PEM containing 1% BSA (A0281, MERCK), 0.1% sodium azide, 100 mM lysine hydrochloride (657-27-2, MERCK)) for 1 h. Cells were then incubated overnight at 4°C with 1:500 anti-Cdc11[60] (a SPB protein; gift from Ken Sawin) or 1:500 anti-CENP-A^{Cnp1} (in-house preparation) in 500 μl of PEMBAL. Cells were then washed three times with PEMBAL and incubated overnight with 1:500 Alexa-488-coupled donkey anti-sheep secondary antibody (A-11015, Invitrogen) in 500 μl of PEMBAL. Cells were then washed in PEMBAL and PEM buffer and re-fixed in 3.7% formaldehyde and 0.25% glutaraldehyde (111-30-8, MERCK) for 15 min, washed with PEM buffer and treated with 1 mg/ml sodium borohydride in PEM buffer. After incubation with 2 μl of 10 mg/ml RNase A (19101, Qiagen) in 100 μl of PEMBAL at 37°C for 2h, cells were denatured in 100 μl of freshly prepared 0.1 M NaOH for 1 min and hybridized with 2 μl of DNA FISH probe in 100 μl hybridization buffer (10% Dextran sulphate (D8906, MERCK), 50% deionized formamide (S4117, MERCK), 2XSSC, 5X Denhardts (D2532, MERCK), 0.5 mg/ml denatured salmon sperm DNA) at 37°C overnight.

For *lys1*, itg7, *itg8* and *neo1R* FISH probe, a ~12.5 kb region (ChrI: 3,727,604-3,737,389 and ChrI: 3,739,857-3,742,327) spanning *lys1* gene, ~15 kb region (ChrI 5,438,081-5,453,142) spraining itg7 locus (ChrI 5,447,817-5,448,235), ~12.5 kb region (ChrI: 5,495,975-5,508,459) spanning *itg8* locus (ChrI: 5,500,986-5,502,881) and ~16.3 kb region (ChrI: 5,513,871-5,530,124) within *neo1R* CENP-A^{Cnp1} domain were amplified by PCR using primers listed in Table S3 respectively. ptetO plasmid was used to make FISH probe for ptetO. Plasmid pMC52, pMC1 was used to make *cc2* and plasmid backbone DNA FISH probes, respectively. *cc2* DNA FISH probe was used to locate *cc2* at endogenous *cen2*, *lys1* and plasmid pcc2 and pHcc2, while plasmid backbone probe was used to locate pHet. FISH probes were obtained by DIG labeling 500 ng DNA (PCR products or plasmids) using DIG-Nick Translation Mix (11745816910, Roche) supplemented with 1 μl of 1:50 diluted DNase I (AM2222, Ambion).

After hybridization with DNA FISH probe, cells were washed with 2XSSC containing 0.1% sodium azide and incubated with 1:100 sheep anti-DIG-Rhodamine (11207750910; Roche) in 100 μl of PBS-BAG (PBS buffer supplemented with 1% BSA (A0281, MERCK), 0.1% sodium azide and 0.5% cold water fish gelatin (G7765, MERCK)) at room temperature overnight. Cells were finally stained with 4',6-diamidino-2-phenylindole (DAPI), mounted in VECTASHIELD Mounting Medium on Polylysine slides and imaged using Nikon NIS Elements software (version 5.21.03) on a Nikon Ti2 inverted microscope as indicated above. All images are scaled relative to the maximum value of histogram.

3D distance measurements

3D distances between spots in two channels (green and red): Cdc11/CENP-A^{Cnp1} (green) and DNA FISH (red) or *lys1:lacO* (*ade3:lacO*)/LacI-GFP (green) and Sad1-dsred (red), were measured by Fiji using in-house script (https://zenodo.org/record/5657360#.Yn7YZBPMLUJ). Briefly, the center of spot in each channel were determined in X-Y using the Fiji "Find Maxima…" function with same threshold (Prominence>500), applied to a Z-projection. The Z-positions of each spot were then determined as the slice with the maximum pixel intensity at each X-Y position. The distance to the nearest red spot for each green spot was reported if within 3 μm representing the diameter of the fission yeast nucleus. If no red spot was detected within 3 μm then that green spot was not included in the analysis. Distances between the resulting spots in each channel were measured by equation:

$$d = \sqrt{(\Delta x^2 + \Delta y^2 + \Delta z^2)}$$

Live mono-nuclear cells 8-12 μm in length and only one SPB (Sad1-dsRed) nucleus-associated dot were recognized as G2 cells and subjected to distance measurements between LacI-GFP (binds to *lys1:lacO* or *ade3:lacO*) and Sad1-dsRed. For immunofluorescence/DNA FISH, mononuclear cells with nuclear green-red spot pairs and only one SPB (Cdc11) or centromere cluster (CENP-A^{Cnp1}) spot were recognized as interphase cells and retained for distance measurement between DNA FISH locus (red) and protein Cdc11 or CENP-A^{Cnp1} (green).

QUANTIFICATION AND STATISTICAL ANALYSIS

All quantification and statistical details of experiments are described in the figure legends or in the methods section. The qChIP results are obtained from more than 3 independent experimental replicates ($n \geq 3$) and represented as mean ± SD (standard deviation, error bars). Significance of the differences in qChIP results was evaluated using Unpaired t test with a p value threshold < 0.05, by Prism Version 9.1.0 software (RRID: SCR_002798). 3D distance measurement results were obtained by analyzing n number of

interphase cells from 3 independent experimental replicates. Average distance for each strain were calculated and indicated as "AV." (specified in figure legends). Cells were classified into three groups according to the distance: overlap (≤0.3 μm), adjacent (0.3-0.5 μm) or separate (0.5-3 μm). The results were reported as percentage of cells (% cells) in each group. For statistical significance analysis of distance data, Mann-Whitney U test with a p value threshold <0.01 was performed by Prism Version 9.1.0 software (RRID: SCR_002798) and the detailed results were showed in Data S1.

Current Biology, Volume *32*

Supplemental Information

Establishment of centromere identity is dependent on nuclear spatial organization

Weifang Wu, Toni McHugh, David A. Kelly, Alison L. Pidoux, and Robin C. Allshire

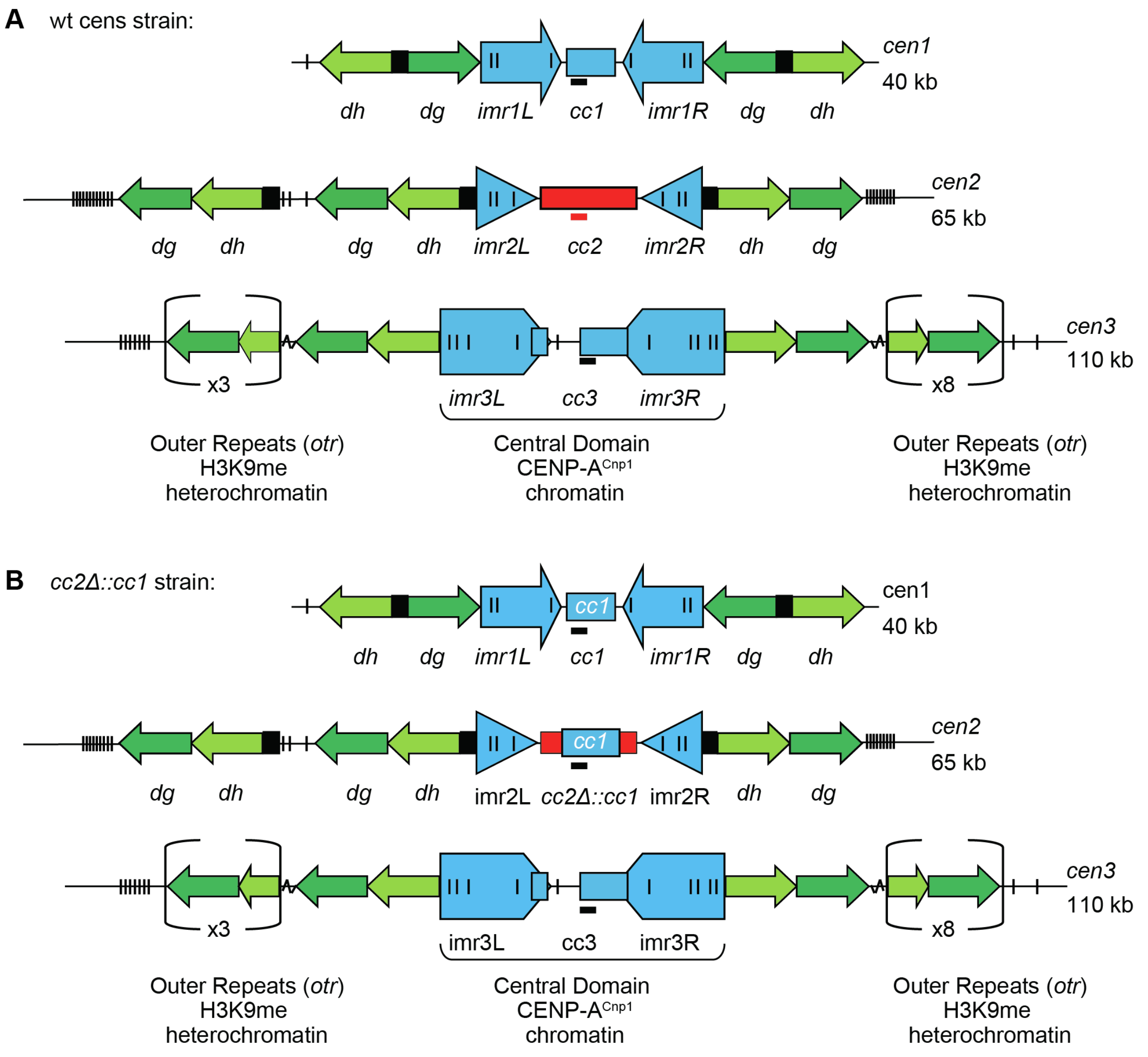

Figure S1. Domain organization of centromeres in fission yeast. Related to Figures 1-to-7.

(A, B) Schematic representation of the three endogenous wild-type centromeres - wt-cens (A) or *cc2*Δ*::cc1* strains (B). Each centromere consists of two distinct domains: a central domain assembled inCENP-A^{Cnp1} chromatin harbouring a central core (*cc*) DNA and flanking innermost repeats (*imr*), which are surrounded by various repetitive DNA elements known as the outer repeats (*otr-dg/dh*) assembled in H3K9me-dependent heterochromatin.[S1] In *cc2*Δ*::cc1* cells,[S2] 6 kb of *cc2* DNA was replaced by 5.5 kb of *cc1* sequence, allowing the specific analysis of unique *cc2* DNA present in ectopic *cc2* insertions and minichromosomes pcc2 and pHcc2. Black, red bars represent qChIP primer sites on *cc1/3* (the homologous region between *cc1* and *cc3*) and *cc2* DNA, respectively.

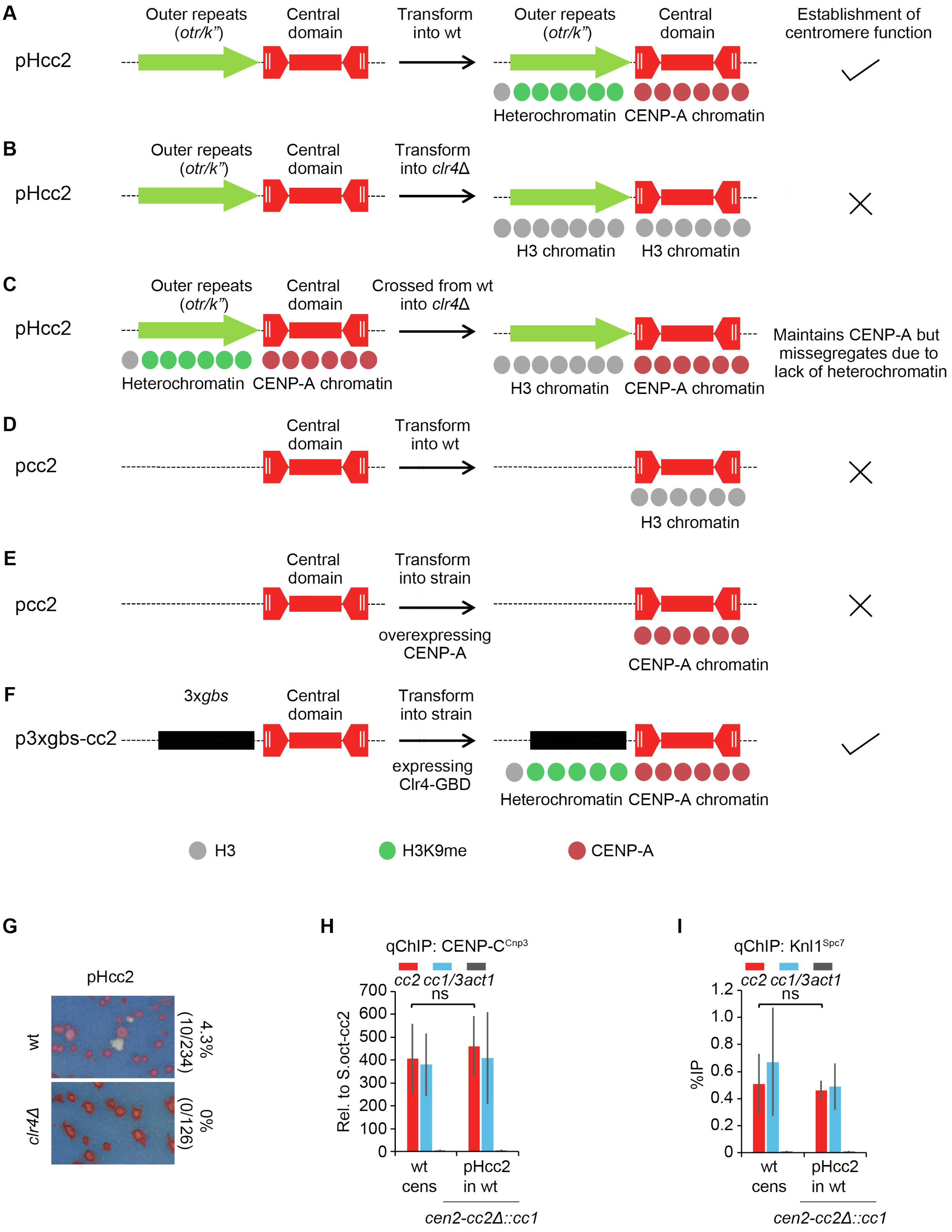
A
Outer repeats (otr/k")
Central domain
Transform into wt
Establishment of centromere function
pHcc2
Heterochromatin
CENP-A chromatin
B
Transform into clr4Δ
H3 chromatin
C
Crossed from wt into clr4Δ
Maintains CENP-A but missegregates due to lack of heterochromatin
D
pcc2
E
Transform into strain overexpressing CENP-A
F
3xgbs
p3xgbs-cc2
Transform into strain expressing Clr4-GBD
H3
H3K9me
CENP-A
G
wt
clr4Δ
4.3% (10/234)
0% (0/126)
H
qChIP: CENP-C^Cnp3
cc2 cc1/3act1
ns
Rel. to S.oct-cc2
wt cens
pHcc2 in wt
cen2-cc2Δ::cc1
I
qChIP: Knl1^Spc7
%IP

Figure S2. Heterochromatin is required to establish CENP-A^{Cnp1} chromatin, establish functional centromeres and recruit kinetochore proteins. Related to Figure 1.

(A, B) pHcc2 assembles heterochromatin on outer repeats (*otr/K"*) which promotes CENP-A^{Cnp1} chromatin establishment on its central domain DNA in wild-type - wt (A) cells but not in *clr4*Δ (B) cells following transformation.[S3] (C) Heterochromatin is not required to maintain CENP-A^{Cnp1} chromatin on central domain of pHcc2 in *clr4*Δ crossed from wild-type.[S3] Although pHcc2 maintains CENP-A^{Cnp1} in *clr4*Δ, it does not segregate properly due to lack of heterochromatin, cohesion and biorientation function. (D, E) pcc2 assembles CENP-A^{Cnp1} chromatin on its central domain in CENP-A^{Cnp1} overexpressed cells (E) but not in wild-type CENP-A^{Cnp1} cells (D) upon transformation.[S2] (F) p3xgbs-cc2 assembles heterochromatin on *3xgbs* (three Gal4-binding sites) that permits CENP-A^{Cnp1} chromatin establishment on its *cc2* central domain in cells expressing Clr4-GBD (the DNA binding domain of the *S. cerevisiae* Gal4 protein) fusions following transformation. Artificial association of Clr4 with *3xgbs* bypasses the requirement for heterochromatic outer repeats (*otr/K"*) in heterochromatin assembly.[S4] "✓" indicates functional centromere can be established on plasmid-based minichromosome, whereas "×" indicates no centromere is established. Both heterochromatin and CENP-A^{Cnp1} are required to form a functional centromere. (G) Functional centromere establishment assay on pHcc2 transformed into wt and *clr4*Δ strains carrying *cc2*Δ*::cc1*. The centromere establishment assay provides a measure of immediate ability to establish functional centromeres. pHcc2 plasmid can establish heterochromatin via the outer repeat region. Heterochromatin promotes establishment of CENP-A^{Cnp1} chromatin on the central core domain (*cc2*). CENP-A^{Cnp1} chromatin is the platform for kinetochore assembly.[S3] Heterochromatin also ensures proper cohesion, meaning that the plasmid can biorient on the spindle and segregate efficiently.[S5,S6] Both heterochromatin and CENP-A are required to form a functional centromere. For the centromere establishment assay pHcc2 was transformed into wt and *clr4*Δ strains and plated on selective plates. Transformants were promptly replica plated to low adenine-containing plates. Pale pink/white colonies indicate the presence of functional centromere since *sup3e* tRNA on pHcc2 suppresses ade6-704 mutation within strains. Establishment frequency was calculated as the percentage of pale pink/white colonies divided by the total number of transformants (minus pure white colonies) (See also STAR Methods), thus 4.3%, 0% of colonies established functional centromeres on pHcc2 transformed into wt and *clr4*Δ cells, respectively. (H, I) qChIP analyses for CENP-C^{Cnp3} (H), Knl1^{Spc7} (I) levels at *cc2, cc1/3* and *act1* in wild-type strains carrying endogenous *cen2-cc2* or *cen2-cc2*Δ*::cc1* strain transformed with pHcc2. For ChIP analyses transformants were picked randomly from selective plates. Replica plating to low adenine plates was not performed. During growth to log phase for ChIP further establishment of centromeres occurs – in up to 100% of wild-type pHcc2 cultures. %IP levels in *S. pombe* were normalized to %IP of central core from spiked-in *S. octosporus* chromatin in (H). qChIP results in (I) were reported as %IP. Data are mean ± SD (n=3). ns, no significance (Unpaired t-test).

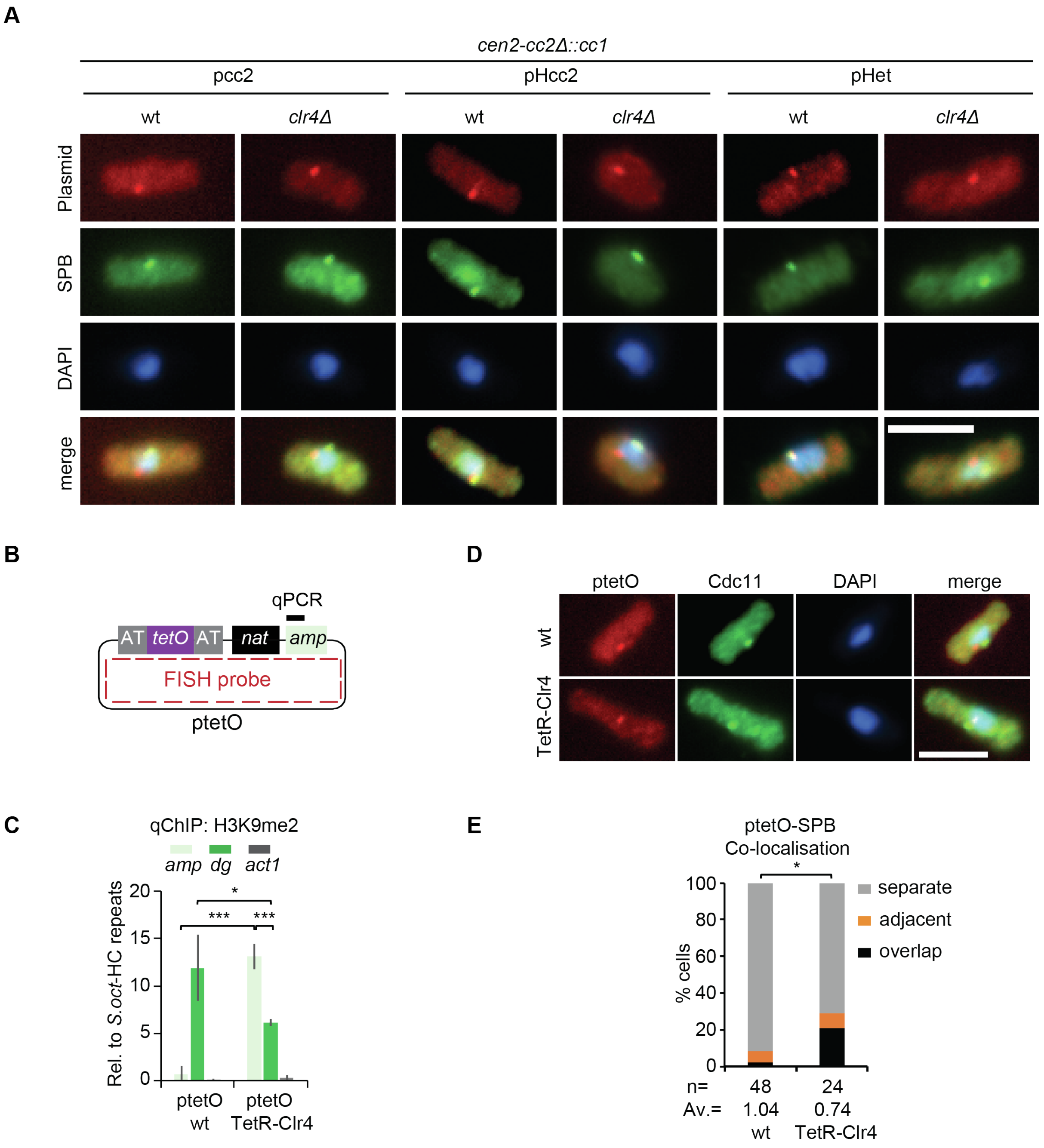
A
cen2-cc2Δ::cc1
pcc2
pHcc2
pHet
wt
clr4Δ
wt
clr4Δ
wt
clr4Δ
Plasmid
SPB
DAPI
merge
B
qPCR
AT
tetO
AT
nat
amp
FISH probe
ptetO
C
qChIP: H3K9me2
amp
dg
act1
Rel. to S.oct-HC repeats
20
15
10
5
0
*
***
***
ptetO
wt
ptetO
TetR-Clr4
D
ptetO
Cdc11
DAPI
merge
wt
TetR-Clr4
E
ptetO-SPB
Co-localisation
*
100
80
60
40
20
0
% cells
separate
adjacent
overlap
n=
48
24
Av.=
1.04
0.74
wt
TetR-Clr4

Figure S3. The assembly of centromeric or synthetic heterochromatin increases the frequency of pHcc2, pHet and ptetO colocalization with SPBs. Related to Figure 1.

(A) Representative images of plasmid DNA FISH (red; probe as indicated in Figure 1B), SPB location (green; anti-Cdc11) and DNA staining (blue, DAPI) in wild-type (*wt*) and *clr4*Δ cells transformed with pcc2, pHcc2 or pHet. Images were scaled relative to the maximum values of histogram. Scale bar, 5 μm. (B) Diagram of ptetO minichromosome. Black bar above plasmid map represents qChIP primer site on ampicillin gene (*amp*). Dashed red line on plasmid indicates position of FISH probe. (C) qChIP analyses for H3K9me2 levels on *amp* gene of ptetO, *dg* repeats of centromeric heterochromatin and *act1* gene. (D) Representative images of ptetO plasmid DNA FISH (red; probe as indicated in A), SPB location (green; anti-Cdc11) and DNA staining (blue, DAPI) in wild-type (wt) and TetR-Clr4 cells transformed with ptetO. Images were scaled relative to the maximum values of histogram. Scale bar, 5 μm. (E) Cells were classified into three groups according to the 3D distances between ptetO and SPB (Cdc11): overlap (≤0.3 μm), adjacent (0.3-0.5 μm) or separate (0.5-3 μm). Percentage of interphase cells (n, number analyzed from 3 independent experiments) in each category. AV., average distance. ns, no significance; *, $p<0.01$ (Mann-Whitney U test).

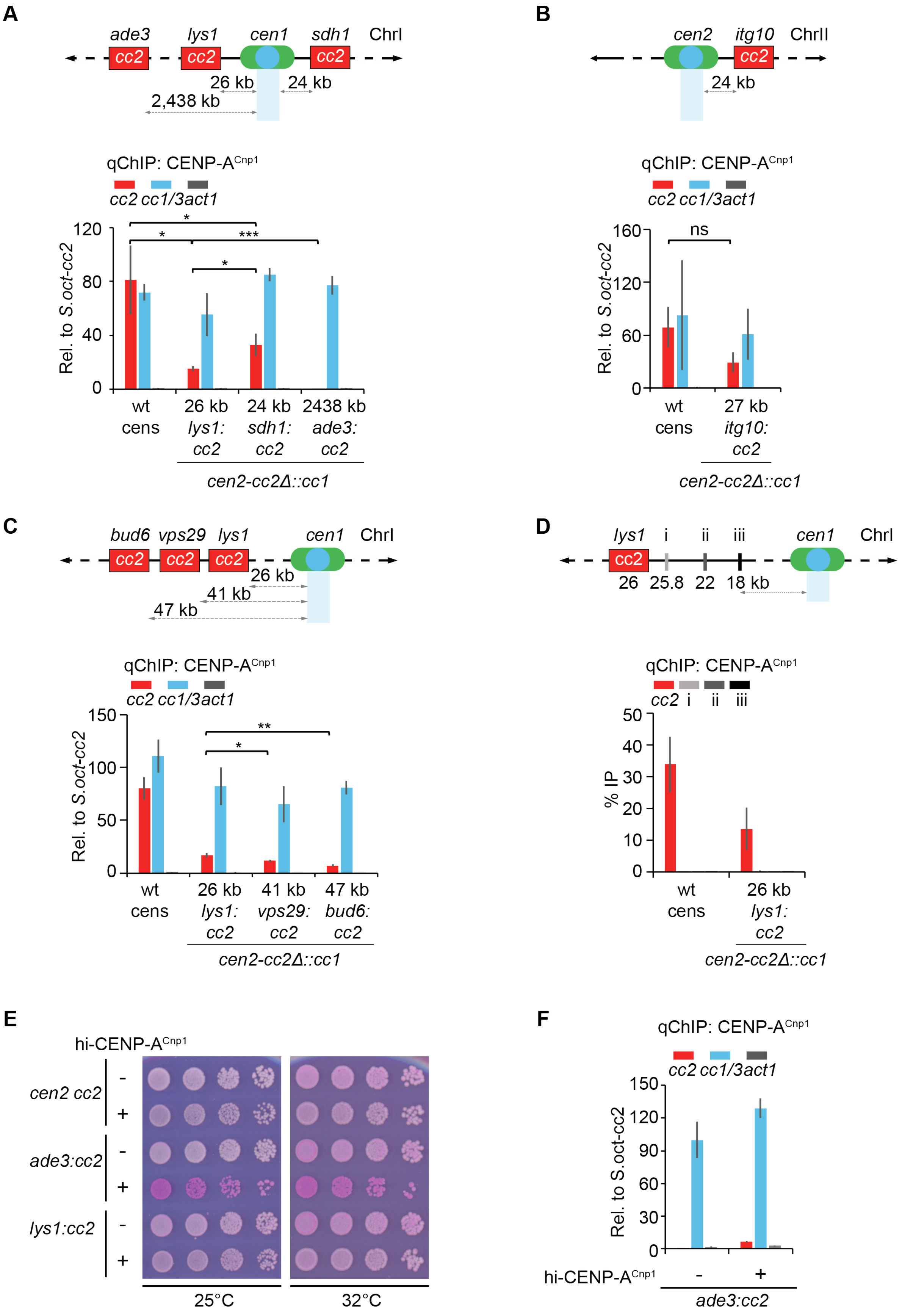
A
ade3
lys1
cen1
sdh1
ChrI
cc2
cc2
cc2
26 kb
24 kb
2,438 kb
qChIP: CENP-A^Cnp1
cc2 cc1/3act1
120
80
40
0
Rel. to S.oct-cc2
*
*
***
*
wt cens
26 kb lys1: cc2
24 kb sdh1: cc2
2438 kb ade3: cc2
cen2-cc2Δ::cc1
B
cen2
itg10
ChrII
cc2
24 kb
qChIP: CENP-A^Cnp1
cc2 cc1/3act1
160
120
60
0
Rel. to S.oct-cc2
ns
wt cens
27 kb itg10: cc2
cen2-cc2Δ::cc1
C
bud6
vps29
lys1
cen1
ChrI
cc2
cc2
cc2
26 kb
41 kb
47 kb
qChIP: CENP-A^Cnp1
cc2 cc1/3act1
150
100
50
0
Rel. to S.oct-cc2
**
*
wt cens
26 kb lys1: cc2
41 kb vps29: cc2
47 kb bud6: cc2
cen2-cc2Δ::cc1
D
lys1
i
ii
iii
cen1
ChrI
cc2
26
25.8
22
18 kb
qChIP: CENP-A^Cnp1
cc2 i ii iii
50
40
30
20
10
0
% IP
wt cens
26 kb lys1: cc2
cen2-cc2Δ::cc1
E
hi-CENP-A^Cnp1
cen2 cc2
-
+
ade3:cc2
-
+
lys1:cc2
-
+
25°C
32°C
F
qChIP: CENP-A^Cnp1
cc2 cc1/3act1
150
120
90
60
30
0
Rel. to S.oct-cc2
hi-CENP-A^Cnp1
-
+
ade3:cc2

Figure S4. CENP-A^{Cnp1} chromatin is established on *cc2* inserted closed to centromeres and CENP-A^{Cnp1} overexpression results in defect growth of cells containing a centromere-distal *ade3:cc2* insertion. Related to Figure 2.

(A-C) qChIP analyses for CENP-A^{Cnp1} levels at *cc2, cc1/3* and *act1* in wt-cens strain with *cen2-cc2* or *cc2Δ::cc1* strain with *lys1:cc2, sdh1:cc2*, *ade3:cc2* (A) or *itg10:cc2* (*itg10*; ChrII: 1,645,855-1,655,523; B) or *vps29:cc2* or *bud6:cc2* (C) insertion. %IP levels in *S. pombe* were normalized to %IP of central core from spiked-in *S. octosporus* chromatin. (D) qChIP analyses for CENP-A^{Cnp1} levels at three euchromatic locus between *lys1* and *cen1*: sites i, ii, iii, 25.8, 22, 18 kb from *cc1* respectively in wt-cens or *cc2Δ::cc1* strain with *lys1:cc2.* qChIP results were reported as %IP. All qChIP data are mean ± SD (n=3). ns, no significance; *, p<0.05; **, p<0.005; ***, p<0.0005 (Unpaired t-test). (E) Overexpression of CENP-A^{Cnp1} leads to growth defect in cells containing centromere-distant *ade3:cc2* insertion**.** Growth of indicated strains carrying endogenous *cen2 cc2* or *ade3:cc2* or *lys1:cc2* expressing wild-type levels (-; wt-CENP-A^{Cnp1}) or high levels of CENP-A^{Cnp1} (+; hi-CENP-A^{Cnp1}) on plates containing the vital dye phoxine B. Colonies with a higher proportion of dead cells stain darker pink. All strains were grown at 25°C or 32°C for 3-5 days and then photographed. (F) qChIP analyses for CENP-A^{Cnp1} levels at *cc2, cc1/3* and *act1* in *cc2Δ::cc1*, *nmt41*-CENP-A^{Cnp1} strain with *ade3:cc2* insertion expressing wild-type levels (-; wt-CENP-A^{Cnp1}) or high levels of CENP-A^{Cnp1} (+; hi-CENP-A^{Cnp1}). %IP levels in *S. pombe* in were normalized to %IP of central core from spiked-in *S. octosporus* chromatin. All qChIP data are mean ± SD (n=3). hi-CENP-A^{Cnp1} expression in A and B is provided by the nmt41 promoter (nmt41-CENP-A^{Cnp1} integrated in genome) under no thiamine conditions.

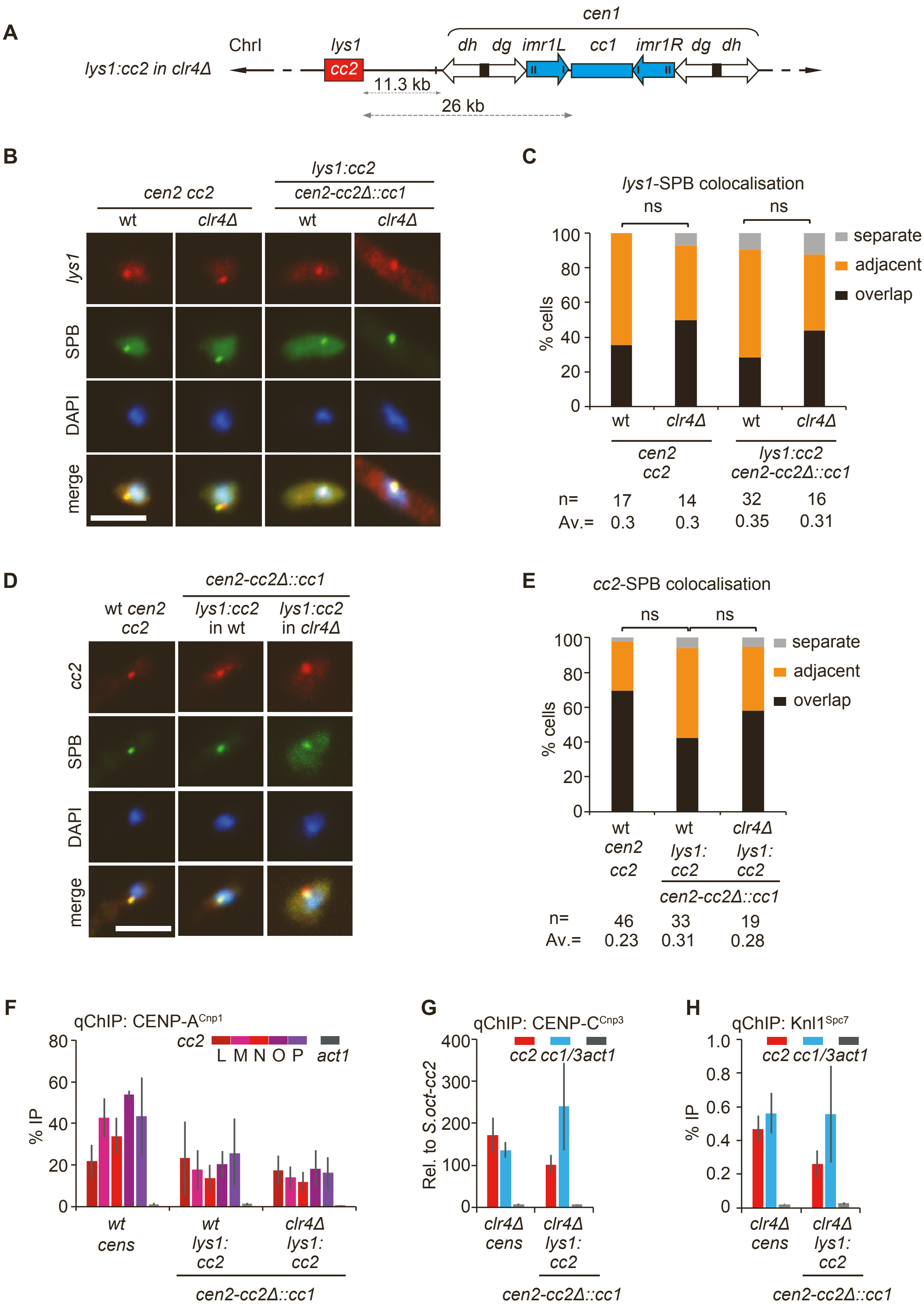
A
lys1:cc2 in clr4Δ
ChrI
lys1
cc2
cen1
dh dg imr1L cc1 imr1R dg dh
11.3 kb
26 kb
B
cen2 cc2
lys1:cc2
cen2-cc2Δ::cc1
wt clr4Δ wt clr4Δ
lys1
SPB
DAPI
merge
C
lys1-SPB colocalisation
ns ns
separate
adjacent
overlap
% cells
0 20 40 60 80 100
wt clr4Δ wt clr4Δ
cen2 cc2
lys1:cc2 cen2-cc2Δ::cc1
n= 17 14 32 16
Av.= 0.3 0.3 0.35 0.31
D
cen2-cc2Δ::cc1
wt cen2 cc2
lys1:cc2 in wt
lys1:cc2 in clr4Δ
cc2
SPB
DAPI
merge
E
cc2-SPB colocalisation
ns ns
separate
adjacent
overlap
% cells
0 20 40 60 80 100
wt cen2 cc2
wt lys1: cc2
clr4Δ lys1: cc2
cen2-cc2Δ::cc1
n= 46 33 19
Av.= 0.23 0.31 0.28
F
qChIP: CENP-ACnp1
cc2 L M N O P act1
% IP
0 20 40 60 80
wt cens
wt lys1: cc2
clr4Δ lys1: cc2
cen2-cc2Δ::cc1
G
qChIP: CENP-CCnp3
cc2 cc1/3 act1
Rel. to S.oct-cc2
0 100 200 300 400
clr4Δ cens
clr4Δ lys1: cc2
cen2-cc2Δ::cc1
H
qChIP: Knl1Spc7
cc2 cc1/3 act1
% IP
0 0.2 0.4 0.6 0.8 1.0
clr4Δ cens
clr4Δ lys1: cc2
cen2-cc2Δ::cc1

Figure S5. Centromeric heterochromatin is not required to establish CENP-A^{Cnp1} chromatin on *cc2* inserted close to *cen1*. Related to Figure 2.

(A) Diagram represents *lys1:cc2* insertion, 26 kb or 11.3 kb from *cc1*, *cen1 dh* repeat in heterochromatin-deficient *clr4Δ* cells following transformation, respectively. (B, D) Representative images of *lys1* (B) or *cc2* (D) DNA FISH (red), SPB location (green; anti-Cdc11) and DNA staining (blue, DAPI) in wild-type (wt) or *clr4Δ* strain with endogenous *cen2-cc2* or *cen2-cc2Δ::cc1* and *lys1:cc2*. Images were scaled as in Figure 1. Scale bar, 5 μm. (C, E) Cells were classified into three groups according to the 3D distances between *lys1* (C) or *cc2* (E) and SPB (Cdc11): overlap (≤0.3 μm), adjacent (0.3-0.5 μm) or separate (0.5-3 μm). Percentage of interphase cells (n, number analyzed from 3 independent experiments) in each category. AV., average distance. ns, no significance (Mann-Whitney U test). (F-H) qChIP analyses for CENP-A^{Cnp1} (F), CENP-C^{Cnp3} (G), Knl1^{Spc7} (H) levels at *cc2*, *cc1/3* and *act1* wt or *clr4Δ* strain with endogenous *cen2-cc2* or *cen2-cc2Δ::cc1* and *lys1:cc2*. %IP levels in *S. pombe* were normalized to %IP of central core from spiked-in *S. octosporus* chromatin in (G). qChIP results in (F, H) were reported as %IP. Data are mean ± SD (n=3).

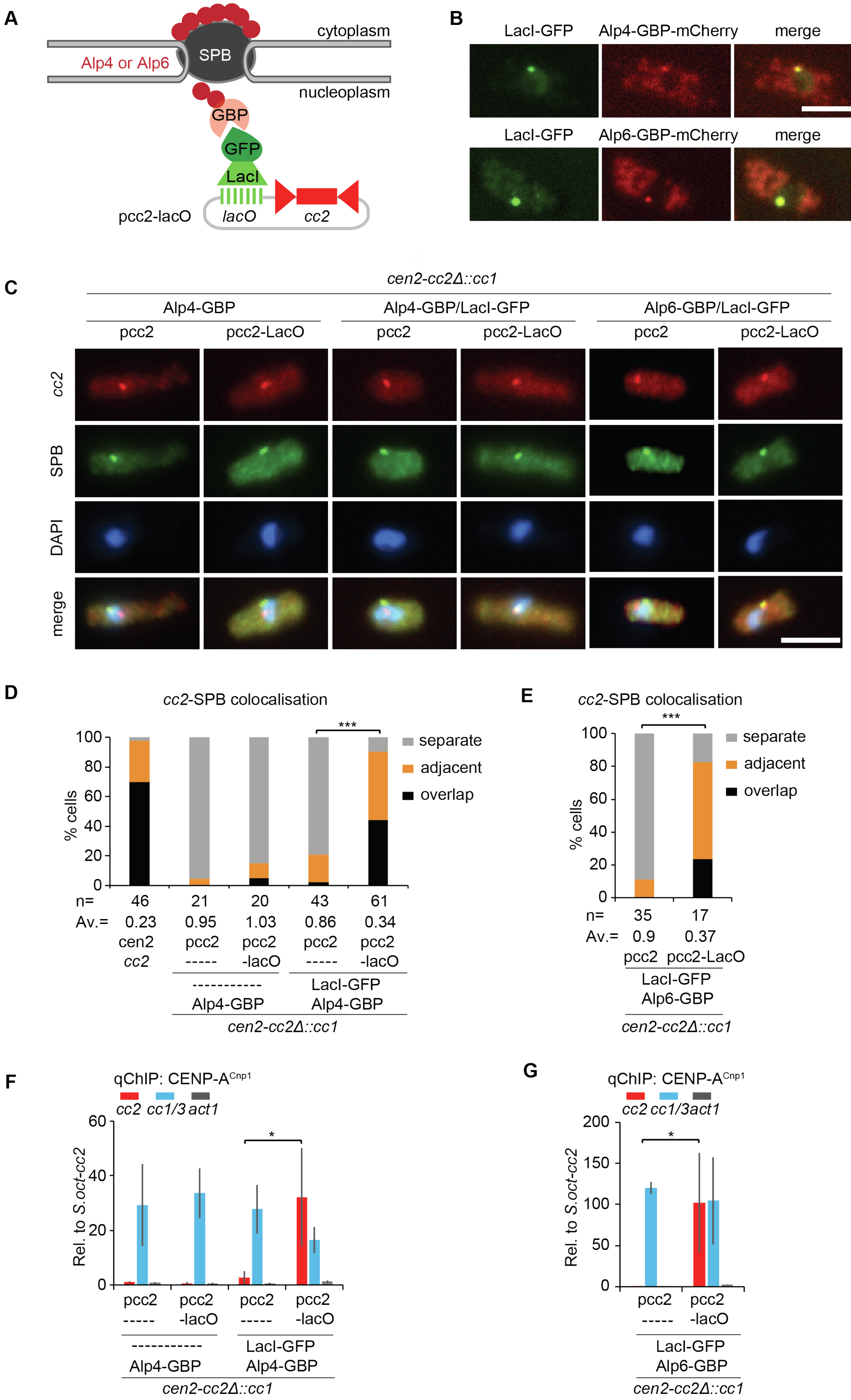

A
cytoplasm
Alp4 or Alp6
SPB
nucleoplasm
GBP
GFP
LacI
pcc2-lacO
lacO
cc2
B
LacI-GFP
Alp4-GBP-mCherry
merge
LacI-GFP
Alp6-GBP-mCherry
merge
C
cen2-cc2Δ::cc1
Alp4-GBP
Alp4-GBP/LacI-GFP
Alp6-GBP/LacI-GFP
pcc2
pcc2-LacO
pcc2
pcc2-LacO
pcc2
pcc2-LacO
cc2
SPB
DAPI
merge
D
cc2-SPB colocalisation
separate
adjacent
overlap
% cells
***
n= 46 21 20 43 61
Av.= 0.23 0.95 1.03 0.86 0.34
cen2 pcc2 pcc2 pcc2 pcc2
cc2 ----- -lacO ----- -lacO

Alp4-GBP
LacI-GFP
Alp4-GBP
cen2-cc2Δ::cc1
E
cc2-SPB colocalisation
separate
adjacent
overlap
% cells
***
n= 35 17
Av.= 0.9 0.37
pcc2 pcc2-LacO
LacI-GFP
Alp6-GBP
cen2-cc2Δ::cc1
F
qChIP: CENP-ACnp1
cc2 cc1/3 act1
Rel. to S.oct-cc2
*
pcc2 pcc2 pcc2 pcc2
----- -lacO ----- -lacO

Alp4-GBP
LacI-GFP
Alp4-GBP
cen2-cc2Δ::cc1
G
qChIP: CENP-ACnp1
cc2 cc1/3 act1
Rel. to S.oct-cc2
*
pcc2 pcc2
----- -lacO
LacI-GFP
Alp6-GBP
cen2-cc2Δ::cc1

Figure S6. Tethering *cc2* DNA to Alp4 or Alp6 allows CENP-A^{Cnp1} incorporation. Related to Figure 5.

(A) Forced association of pcc2-lacO with Alp4 or Alp6-GBP-mCherry at SPB using same tethering system as in Figure 5. A small subset of Alp4 or Alp6 molecules (red circles) are localized to the nucleoplasmic side of SPB during interphase.[S7] (B) Representative images of live cells expressing LacI-GFP and Alp4 or Alp6-GBP-mCherry. Images were scaled as in Figure 2. Scale bar, 5 μm. (C) Representative images of *cc2* DNA FISH (red), SPB location (green; anti-Cdc11) and DNA staining (blue, DAPI) in indicated strains. Images were scaled as in Figure 1. Scale bar, 5 μm. (D, E) Cells were classified into three groups according to the 3D distances between *cc2* and SPB (Cdc11): overlap (≤0.3 μm), adjacent (0.3-0.5 μm) or separate (0.5-3 μm). Percentage of interphase cells (n, number analyzed from 3 independent experiments) in each category. AV., average distance. ***, $p < 0.0001$ (Mann-Whitney U test). (F, G) qChIP analyses for CENP-A^{Cnp1} levels at *cc2*, *cc1/3* and *act1* in indicated strains. %IP levels in *S. pombe* were normalized to %IP of central core from spiked-in *S. octosporus* chromatin. Data are mean ± SD (n=3-4). *, $p<0.05$ (Unpaired t-test).

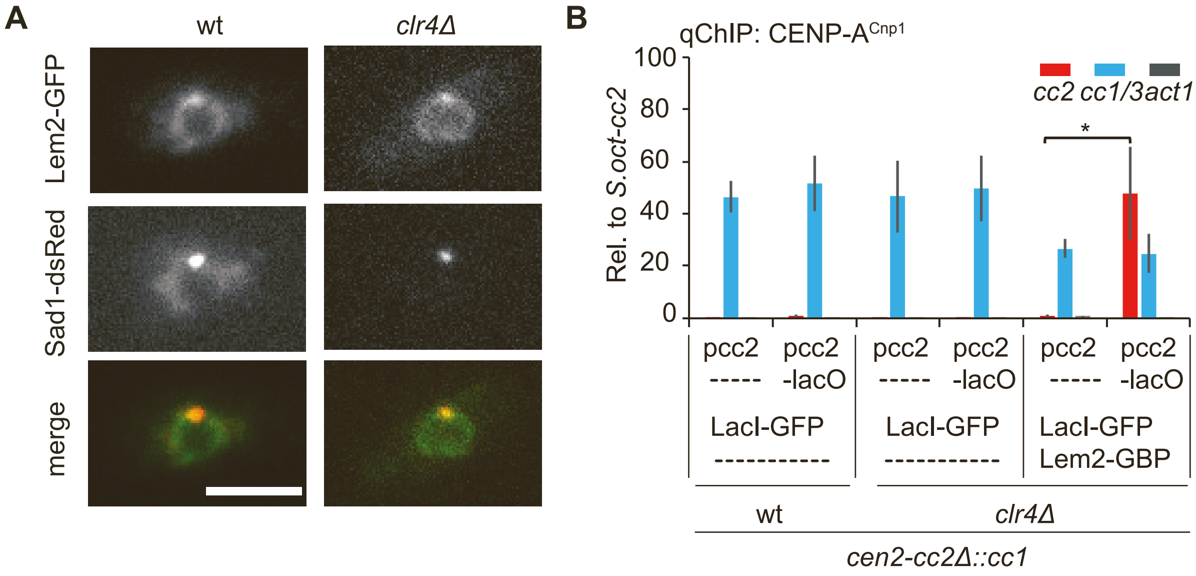

Figure S7. Tethering *cc2* DNA to Lem2 allows CENP-A^{Cnp1} incorporation independently of heterochromatin. Related to Figure 5.

(A) Representative images of live wild-type (wt) or *clr4*Δ cells expressing Lem2-GFP and Sad1-dsRed. Images were scaled as in Figure 2. Scale bar, 5 µm. (B) qChIP analyses for CENP-A^{Cnp1} levels at *cc2*, *cc1/3* and *act1* in wt or *clr4*Δ strains carrying *cen2-cc2*Δ*::cc1* and expressing LacI-GFP or both LacI-GFP and Lem2-GBP-mCherry transformed with pcc2 or pcc2-lacO. %IP levels in *S. pombe* were normalized to %IP of central core from spiked-in *S. octosporus* chromatin. Data are mean ± SD (n=3). *, $p<0.05$ (Unpaired t-test).

**Strain number**	**Genotype**	**abbreviated as**
A7374	*h+ ade6-704-HYGMX6 his3-D1 leu1-32 ura4-DSE/D18? arg3? cc2D6kb:cc1*	*wt cc2Δ::cc1*
B6596	*h+ clr4Δ ade6-704-HYGMX6 his3-D1 leu1-32 ura4-DSE/D18? arg3? cc2D6kb:cc1*	*clr4Δ*
B3665	*h+ lys1(=cen1):lacO his7+:lacIGFP, Sad1-dsRed-LEU2+*	*lys1:lacO*
B3672	*h? Sad1-dsRed-LEU2+ his7+::lacI-GFP ade3[::kanR-ura4+-lacOp] ade6-704-hygMX6 ura4-DSE/D18 leu1-32? his3-D1? arg3-D4? Lys1-131?#1*	*ade3:lacO*
A1741	*h- ade6-704-HYGMX6 his3-D1 leu1-32 ura4-DSE*	*wt cen2*
B2950	*h- lys1Δ500::cc2-KANMX6 ade6-704-HYGMX6 his3-D1 leu1-32 ura4-DSE/D18? arg3? cc2D6kb:cc1#1*	*lys1:cc2 #1* or *lys1:cc2* in wt
B2951	*h- lys1Δ500::cc2-KANMX6 ade6-704-HYGMX6 his3-D1 leu1-32 ura4-DSE/D18? arg3? cc2D6kb:cc1#2*	*lys1:cc2 #2*
B2953	*h- ade3Δ500::cc2-KANMX6 ade6-704-HYGMX6 his3-D1 leu1-32 ura4-DSE/D18? arg3? cc2D6kb:cc1#1*	*ade3:cc2 #1*
B2954	*h- ade3Δ500::cc2-KANMX6 ade6-704-HYGMX6 his3-D1 leu1-32 ura4-DSE/D18? arg3? cc2D6kb:cc1#2*	*ade3:cc2 #2*
A7373	*h- ade6-704-HYGMX6 his3-D1 leu1-32 ura4-DSE/D18? arg3? cc2D6kb:cc1*	*wt cen1* or *wt*
A9792	*h- leu1 ura4 ∆cen1::pADH1-loxP-KanR::ura4+ cd60 (neo1R)*	*cen1Δ neo1R*
B3269	*h? lys1Δ500::cc2-KANMX6 ade6-704-HYGMX6 cc2D6kb:cc1 cen1∆::pADH1-loxP-KanR::ura4+ cd60 (neo1R) his3-D1/His+? leu1-32 ura4?/ura4-DSE/D18? arg3?*	*lys1:cc2* in *neo1R*
B3260	*h- ChrI 5,435,010-5,435,237Δ226bp::cc2-KANMX6 cen1∆::pADH1-loxP-KanR::ura4+ cd60 (neo1R) ade6-704-HYGMX6 cc2D6kb:cc1 his3-D1 leu1-32 ura4-DSE/D18? arg3?*	*itg6:cc2* in *neo1R*
B3265	*h- ChrI5,447,816-5,448,235Δ400::cc2-KANMX6 cen1∆::pADH1-loxP-KanR::ura4+ cd60 (neo1R) ade6-704-HYGMX6 cc2D6kb:cc1 his3-D1 leu1-32 ura4-DSE/D18? arg3?*	*itg7:cc2* in *neo1R*
B3565	*h- ChrI5,501,647-5,502,134Δ528::cc2-KANMX6 cen1∆::pADH1-loxP-KanR::ura4+ cd60 (neo1R) ade6-704-HYGMX6 cc2D6kb:cc1 his3-D1 leu1-32 ura4-DSE/D18? arg3?*	*itg8:cc2* in *neo1R*
B3561	*h- ChrI5,501,647-5,502,134Δ528::cc2-KANMX6 ade6-704-HYGMX6 his3-D1 leu1-32 ura4-DSE/D18? arg3? cc2D6kb:cc1*	*itg8:cc2* in *wt cen1*
B4295	*h+ Lem2-GFP-Nat, Sad1-dsRed-LEU2+ ade6-210 ura4-D18*	Lem2-GFP

B3437	*h+ his7+:lacI-GFP cc2D6kb:cc1 ura4-DSE/D18? leu1-32 ade6-DNN arg3? his3+*	LacI-GFP
B4246	*h+ Lem2-GBP-mcherry-Hyg Sad1-dsRed-LEU2+ cc2D6kb:cc1 ade6-210 his3-D1? leu1-32? ura4-DSE/D18? arg3?*	Lem2-GBP
B4216	*h+ Lem2-GBP-mcherry-Hyg his7+:lacI-GFP cc2D6kb:cc1 ura4-DSE/D18? leu1-32 ade6-DNN arg3? his3+*	LacI-GFP<br>Lem2-GBP
B4339	*h- Lem2-GFP-Nat Sad1-dsRed-LEU2+ Csi1::Kan cc2Δ6kb:cc1 ade6-210 ura4-D18 his3-D1? leu1-32*	Lem2-GFP<br>*csi1Δ*
B4866	*h? Lem2-GBP-mcherry-Hyg, his7+:lacI-GFP csi1Δ::KanMX cc2D6kb:cc1 ade6-DNN his3-D1/ his3+? leu1-32 ura4-DSE/D18? arg3?*	LacI-GFP<br>Lem2-GBP<br>*csi1Δ*
B4865	*h? Lem2-GBP-mcherry-Hyg csi1Δ::KanMX cc2D6kb:cc1 ade6-DNN his3-D1/his3+? leu1-32 ura4-DSE/D18? arg3?*	Lem2-GBP<br>*csi1Δ*
B4906	*h? Sad1-dsRed-LEU2+ his7+::lacI-GFP Csi1::Kan cc2Δ6kb:cc1 ade6-704-hygMX6 ura4-DSE/D18 leu1-32 his3-D1? arg3-D4?*	LacI-GFP<br>*csi1Δ*
B3633	*h+ csi1Δ::KanMX cc2Δ6kb:cc1 ade6-704-HYGMX6 his3-D1 leu1-32 ura4-DSE/D18? arg3?*	*csi1Δ*
B3367	*h- sdh1Δ3344::cc2-KANMX6 ade6-704-HYGMX6 his3-D1 leu1-32 ura4-DSE/D18? arg3? cc2D6kb:cc1*	*sdh1:cc2*
B3639	*h- SPBC21B10.09-10.08cΔ650::cc2-KANMX6 ade6-704-HYGMX6 his3-D1 leu1-32 ura4-DSE/D18? arg3? cc2D6kb:cc1*	*itg10:cc2*
B3211	*h- SPAC15E1.02cΔ158bp::cc2-KANMX6 ade6-704-HYGMX6 his3-D1 leu1-32 ura4-DSE/D18? arg3? cc2D6kb:cc1*	*bud6:cc2*
B3297	*h- vps29Δ154::cc2-KANMX6 ade6-704-HYGMX6 his3-D1 leu1-32 ura4-DSE/D18? arg3? cc2D6kb:cc1*	*vps29:cc2*
A7255	*h+ clr4::NAT ade6-210 his3-D1 leu1-32 ura4-D18 arg3-D4 cc2D6kb:cc1*	*clr4Δ*<br>*cc2Δ::cc1*
B3257	*h+ clr4::NAT lys1Δ500::cc2-KANMX6 ade6-210 his3-D1 leu1-32 ura4-D18 arg3-D4 cc2D6kb:cc1*	*lys1:cc2* in<br>*clr4Δ*
8946	*h- clr4:NAT ade6-210 leu1-32 ura4-D18 arg3-D his3D*	*clr4Δ*<br>*cen2 cc2*
B5560	*h? cc2Δ6kb:cc1 Alp4-GBP-mcherry-Hyg his7+:lacI-GFP leu1-32 ura4-D18/DS-E ade6-704::hphMX/ade6-DNN? his3? arg3?*	LacI-GFP<br>Alp4-GBP
B5564	*h? cc2Δ6kb:cc1 Alp4-GBP-mcherry-Hyg leu1-32 ura4-D18/DS-E ade6-704::hphMX/ade6-DNN? his3? arg3?*	Alp4-GBP
B5572	*h? cc2Δ6kb:cc1 Alp6-GBP-mcherry-Hyg his7+:lacI-GFP leu1-32 ura4-D18/DS-E ade6-704::hphMX/ade6-DNN? his3? arg3?*	LacI-GFP<br>Alp6-GBP
B4349	*h- lem2-GBP-mcherry-Hyg, his7+:lacI-GFP, clr4::NAT, cc2D6kb:cc1 ura4-DSE/D18? his3+/his3-D1? arg3? leu1-32 ade6-DNN/ade6?*	LacI-GFP<br>Lem2-GBP<br>*clr4Δ*
B4351	*h? his7+:lacI-GFP clr4::NAT cc2D6kb:cc1 ura4-DSE/D18? his3+/his3-D1? arg3? leu1-32 ade6-DNN/ade6?*	LacI-GFP<br>*clr4Δ*

B4343	*h- lem2-GFP-Nat Sad1-dsRed-LEU2+ clr4::hph+ ade6-210 ura4-D18 his3-D1? leu1-32*	Lem2-GFP *clr4Δ*
1646	*h- ade6-210 arg3-D4 his3-D1 leu1-32 ura4-D18*	wt
B3797	*h+ leu1+adh21-TetROFF-2xFLAG-Clr4-cdd*	TetR-Clr4
B3416	*h- ars1(MluI)::pREP41XCnp1-leu2Sc ade6-704-HYGMX6 leu1-32 arg3-D4? his3-D1 ura4-DSE/D18? cc2D6kb:cc1*	*cen2 cc2*, hi CENP-A^{Cnp1}
B6527	*h? ars1(MluI)::pREP41XCnp1-leu2Sc lys1Δ500::cc2-KANMX6, cc2D6kb:cc1 ade6-704-HYGMX6 leu1-32 arg3-D4? his3-D1 ura4-DSE/D18?*	*lys1:cc2*, hi CENP-A^{Cnp1}
B3539	*h? ade3Δ500::cc2-KANMX6 ars1(MluI)::pREP41XCnp1-leu2Sc ade6-704-HYGMX6? leu1-32 arg3-D4? his3-D1 ura4-DSE/D18? cc2D6kb:cc1*	*ade3:cc2*, hi CENP-A^{Cnp1}

Table S1. All strains used in this study. Related to STAR Methods.

Name	Plasmid features							Notes	Reference
	marker 1	marker 2	marker 3	cc2	*K"*	*lacO or tetO*	Misc.		
pMC52			*kan*					Used for ectopic *cc2* insertion and *cc2* FISH probe	This study
pMC2 (pcc2)	*ura4*	*sup3e*	*kan*	8.6 kb				Used for tethering assay	This study
pMC12 (pcc2-lacO)	*ura4*	*sup3e*	*kan*	8.6 kb		2.8 kb; ~90 *lacO* sites		Used for tethering assay	This study
pHcc2	*ura4*	*sup3e*		8.6 kb	5.6 kb			Used for mini-chromosome establishment assay	This study
pMC183 (pHet)			*nat*		2 kb			Used to check centromeric heterochromatin nuclear localization	This study
pMC1	*ura4*	*sup3e*	*kan*					Used for plasmid backbone FISH probe	This study
pLSB-Kan			*kan*				Cas9	Used to make *clr4*Δ mutant	From Torres-Garcia.[S8]
clr4-pLSB-Kan			*kan*				Cas9 & *clr4* sgRNA	Used to make *clr4*Δ mutant	This study
pFA6a-GBP-mCherry-hygMX6			*hyg*				GBP & mCherr y	Used to make Lem2/Alp4/Alp6-GBP-mCherry-Hgy fusion protein	Gift from Julia Promisel Cooper.[S9]
pFA6a-GFP-NatMX6			*nat*				GFP	Used to make Lem2-GFP fusion protein	This study
pMC171 (ptetO)			*nat*			4xtetO within 2 kb of random-ized AT-rich DNA		Used for synthetic heterochromatin assembly	This study

Table S2. All plasmids used in this study, Related to STAR Methods

Supplemental references

S1. Allshire, R.C., and Ekwall, K. (2015). Epigenetic Regulation of Chromatin States in *Schizosaccharomyces pombe*. Cold Spring Harb. Perspect. Biol. *7*, a018770.

S2. Catania, S., Pidoux, A.L., and Allshire, R.C. (2015). Sequence features and transcriptional stalling within centromere DNA promote establishment of CENP-A chromatin. PLoS Genet. *11*, e1004986.

S3. Folco, H.D., Pidoux, A.L., Urano, T., and Allshire, R.C. (2008). Heterochromatin and RNAi are required to establish CENP-A chromatin at centromeres. Science *319*, 94-97.

S4. Kagansky, A., Folco, H.D., Almeida, R., Pidoux, A.L., Boukaba, A., Simmer, F., Urano, T., Hamilton, G.L., and Allshire, R.C. (2009). Synthetic heterochromatin bypasses RNAi and centromeric repeats to establish functional centromeres. Science *324*, 1716-1719.

S5. Bernard, P., Maure, J.F., Partridge, J.F., Genier, S., Javerzat, J.P., and Allshire, R.C. (2001). Requirement of heterochromatin for cohesion at centromeres. Science *294*, 2539-2542.

S6. Nonaka, N., Kitajima, T., Yokobayashi, S., Xiao, G., Yamamoto, M., Grewal, S.I., and Watanabe, Y. (2002). Recruitment of cohesin to heterochromatic regions by Swi6/HP1 in fission yeast. Nat. Cell Biol. *4*, 89-93.

S7. Bestul, A.J., Yu, Z., Unruh, J.R., and Jaspersen, S.L. (2017). Molecular model of fission yeast centrosome assembly determined by superresolution imaging. J. Cell Biol. *216*, 2409-2424.

S8. Torres-Garcia, S., Di Pompeo, L., Eivers, L., Gaborieau, B., White, S.A., Pidoux, A.L., Kanigowska, P., Yaseen, I., Cai, Y., and Allshire, R.C. (2020). SpEDIT: A fast and efficient CRISPR/Cas9 method for fission yeast. Wellcome Open Res. *5*, 274.

S9. Fernández-Álvarez, A., Bez, C., O'Toole, Eileen T., Morphew, M., and Cooper, Julia P. (2016). Mitotic Nuclear Envelope Breakdown and Spindle Nucleation Are Controlled by Interphase Contacts between Centromeres and the Nuclear Envelope. Dev. Cell *39*, 544-559.
